# *Prunus Hexokinase 3* genes alter primary C-metabolism and promote drought and salt stress tolerance in Arabidopsis transgenic plants

**DOI:** 10.1038/s41598-021-86535-1

**Published:** 2021-03-29

**Authors:** Jorge Pérez-Díaz, Willian Batista-Silva, Rubén Almada, David B. Medeiros, Stéphanie Arrivault, Francisco Correa, Adriana Bastías, Pamela Rojas, María Francisca Beltrán, María Francisca Pozo, Wagner L. Araújo, Boris Sagredo

**Affiliations:** 1 Instituto de Investigaciones Agropecuarias CRI Rayentué, Av. Salamanca s/n, Sector Los Choapinos, Rengo, Chile; 2 Max Planck Partner Group at the Departamento de Biologia Vegetal, Universidade Federal de Viçosa, Viçosa, MG 36570-900 Brazil; 3 Centro de Estudios Avanzados en Fruticultura, CEAF, Camino Las Parcelas 882, Sector Los Choapinos, Rengo, Chile; 4 Max Planck Institute of Molecular Plant Physiology, Am Mühlenberg 1, 14476 Potsdam-Golm, Germany; 5 Universidad Autónoma de Chile, Av. Pedro de Valdivia 425, Providencia, Santiago, Chile

**Keywords:** Transgenic plants, Abiotic

## Abstract

Hexokinases (HXKs) and fructokinases (FRKs) are the only two families of enzymes in plants that have been identified as able to phosphorylate Glucose (Glc) and Fructose (Fru). Glc can only be phosphorylated in plants by HXKs, while Fru can be phosphorylated by either HXKs or FRKs. The various subcellular localizations of HXKs in plants indicate that they are involved in diverse functions, including anther dehiscence and pollen germination, stomatal closure in response to sugar levels, stomatal aperture and reducing transpiration. Its association with modulating programmed cell death, and responses to oxidative stress and pathogen infection (abiotic and biotic stresses) also have been reported. To extend our understanding about the function of HXK-like genes in the response of Prunus rootstocks to abiotic stress, we performed a detailed bioinformatic and functional analysis of hexokinase 3-like genes (*HXK3*s) from two Prunus rootstock genotypes, ‘M.2624’ (*Prunus cerasifera* Ehrh × *P. munsoniana* W.Wight & Hedrick) and ‘M.F12/1’ (*P. avium* L.), which are tolerant and sensitive to hypoxia stress, respectively. A previous large-scale transcriptome sequencing of roots of these rootstocks, showed that this *HXK3-like* gene that was highly induced in the tolerant genotype under hypoxia conditions. In silico analysis of gene promoters from M.2624 and M.F12/1 genotypes revealed regulatory elements that could explain differential transcriptional profiles of *HXK3* genes. Subcellular localization was determinates by both bioinformatic prediction and expression of their protein fused to the green fluorescent protein (GFP) in protoplasts and transgenic plants of Arabidopsis. Both approaches showed that they are expressed in plastids. Metabolomics analysis of Arabidopsis plants ectopically expressing *Prunus HXK3* genes revealed that content of several metabolites including phosphorylated sugars (G6P), starch and some metabolites associated with the TCA cycle were affected. These transgenic Arabidopsis plants showed improved tolerance to salt and drought stress under growth chamber conditions. Our results suggest that *Prunus HXK3* is a potential candidate for enhancing tolerance to salt and drought stresses in stone fruit trees and other plants.

## Introduction

The primary products of photosynthesis are sugars like sucrose and the hexoses, glucose (Glc) and fructose (Fru), which form the basis of most organic matter^1^. Plants require rigorous sugar sensing and signaling mechanisms to regulate many essential processes, such as photosynthesis, carbon metabolism, growth, senescence and defense responses to biotic and abiotic stresses^2^. Sugars affect practically all plant cell processes in plant cells by providing skeletons for organic compounds and storing energy for chemical reactions^3,4^. Before they are used in metabolic processes, glucose and fructose are phosphorylated. The role of hexose-phosphorylating enzymes, such as hexokinases (HXKs) and fructokinases (FRKs) has attracted the attention of many researchers^1^. Glc in plants can only be phosphorylated by HXKs, while Fru can be phosphorylated by either HXKs or FRKs. Catalyzing irreversible reactions, these enzymes are important in regulating plant metabolism and sugar signaling^5^. The HXK family in *Arabidopsis thaliana* is represented by six members, three of which phosphorylate hexoses, while the other three lack this catalytic activity and they are known as HXK-like (HKL) proteins^6^. *AtHXK1,* encodes an enzyme with the dual function of mediating sugar sensing and catalyzing hexose-phosphorylation^7^. *HXK* genes from several plant species have been isolated^1^. HXKs are commonly classified into two major groups, Types A and B, based on their N-terminal amino acid sequences^8^. Type A HXKs, like AtHXK3 in *A. thaliana*, OsHXK4 in *O. sativa*, and LeHXK4 in *L. esculentum,* are located in plastid stroma and have a chloroplast transit peptide with approximately 30 amino acids^1^. Type B HXKs possess a common hydrophobic membrane anchor domain and are associated with mitochondria, such as Arabidopsis AtHXK1 and AtHXK2, tomato SlHXK1, 2 and 3, *Nicotiana benthamiana* NbHXK1, rice OsHXK2, 3, 5, 6 and 9, among others^1^. A new HXK type (Type C) has recently been reported, the rice OsHXK1, 7, and 8, which are located in the cytoplasm and nucleus, since they lack the plastidic transit peptide and the membrane anchor domain^9^. Type D HXKs are encoded by three genes, with membrane anchors and are associated with the mitochondria, but their sequences differ from those of the type B HXK^1^. The type D HXK of *Physcomitrella patens,* PpHXK1, is also located in the chloroplast envelope^10^. The multiple subcellular localizations of HXKs in plants indicate that they play diverse functions. The rice *OsHXK10* is involved in anther dehiscence and pollen germination^11^. HXKs also mediate stomatal closure in response to sugar levels^12^. For instance, the expression of *AtHXK1* in citrus guard cells controls stomatal aperture and reduces transpiration^13^. *AtHXK1* controls PIP aquaporin expression to reduce hydraulic conductance in response to increasing sugar levels during the photoperiod, perhaps as an additional means of preserving plant water content^14^. It has been reported that these proteins are also involved in modulating programmed cell death in plants^15^, and responses to oxidative stress and pathogen infection (abiotic and biotic stresses)^16^.

Abiotic stresses such drought, salinity, hypoxia and extreme temperatures also affect stone fruit tree growth and orchard yields. Stone fruit trees belong to the *Prunus* genus, a member of the Rosaceae family, and their fruits constitute a rich source of vitamins, minerals, fiber and antioxidant compounds for healthy diets. Chile is an important actor regarding global fruit supply mainly due to its position in the southern hemisphere and its ability to produce stone fruits (e.g. sweet cherries, plums and peaches) in seasons when production in the northern hemisphere is negligible^17^. However, the Chilean leadership in the stone fruit markets as well as their role as food supplier could be under “checkmate” since the predicted global climate change present challenges to the future agricultural productivity. Several studies suggest that most of the cultivated perennial crops, including stone fruit trees are predicted to exhibit severe fruit yield and quality reductions under future climate change scenarios^18–20^. As a rule, stone fruit trees are grafted on rootstocks, which either belong to the same or other species or inter-specific hybrids of the *Prunus* genus. The influences of the rootstock system on scion are profound because they determine fruit quality and also they play a pivotal role in water and nutrient uptake, plant-soil environment interactions and the tree tolerance to several abiotic stresses^21^. However, few studies have tackled the physiological, biochemical and molecular adjustments taking place in *Prunus* rootstock roots under abiotic stresses such as drought^22^ or hipoxia^23–25^. In order to gain insights into the molecular basis of stone fruit rootstock responses to low-oxygen environments, we performed a large-scale transcriptome sequencing of roots from two different stone fruit rootstocks with contrasting responses to hypoxia (Mariana 2624, *Prunus cerasifera* × *Prunus munsoniana* and Mazzard F12/1, *Prunus avium* L., which are tolerant and sensitive to this stress, respectively) and revealed several hypoxia-responsive genes^23^. Outstandingly, a hexokinase *3*-like gene (*HXK3*) was highly induced in the tolerant genotype suggesting a role in the rootstock response(s) to oxygen deprivation^23^. To extend our understanding about the function of *HXK*-like genes in the response of *Prunus* rootstocks to abiotic stress, we performed a detailed bioinformatic and functional analysis of *HXK3* genes from two rootstock genotypes (‘M.2624’ and ‘M.F12/1’). In silico analysis of gene promoters from M.2624 and M.F12/1 genotypes revealed regulatory elements that could explain differential transcriptional profiles of *HXK3* genes. *Prunus* HXKs were localized in plastids. Metabolomics analysis of Arabidopsis plants ectopically expressing *Prunus HXK3* genes revealed that the amounts of several metabolites including phosphorylated sugars (G6P), starch and some metabolites associated with the TCA cycle were affected. These transgenic Arabidopsis plants showed improved tolerance to salt and drought stress. Our results suggest that *Prunus HXK3* is a potential candidate for enhancing tolerance to salt and drought stresses in stone fruit trees and other plants.

## Results

### *Prunus HXK3* is induced by root hypoxia

The expression of the *HXK3* gene was measured by RT-qPCR in roots from ‘M.2624’ and ‘M.F12/1’ plants under root hypoxia caused by flooding as previously described by Arismendi et al.^23^ (Supplementary Fig. S1). The plants showed the same phenotypic changes as described previously, e.g. ‘M.F12/1’ was highly sensitive whereas ‘M.2624’ was highly tolerant. In ‘M.2624’ the *HXK3* gene was highly induced after only 3 h of root hypoxia, reaching a maximum at 12 h when a 120-fold induction under flooding conditions compared to control was observed and maintaining elevated expression for up to 7 days. In contrast, maximum *HXK3* expression in ‘M.F12/1’ occurred after 3 days of root hypoxia reaching a tenfold induction under flooding conditions compared to control. However, the expression of *HXK3* under root hypoxia was considerably lower in the sensitive cultivar (Supplementary Fig. S1).

### Analysis of *Prunus HXK3* promoters reveals the presence of elements related to abiotic stress

**Figure 1:**
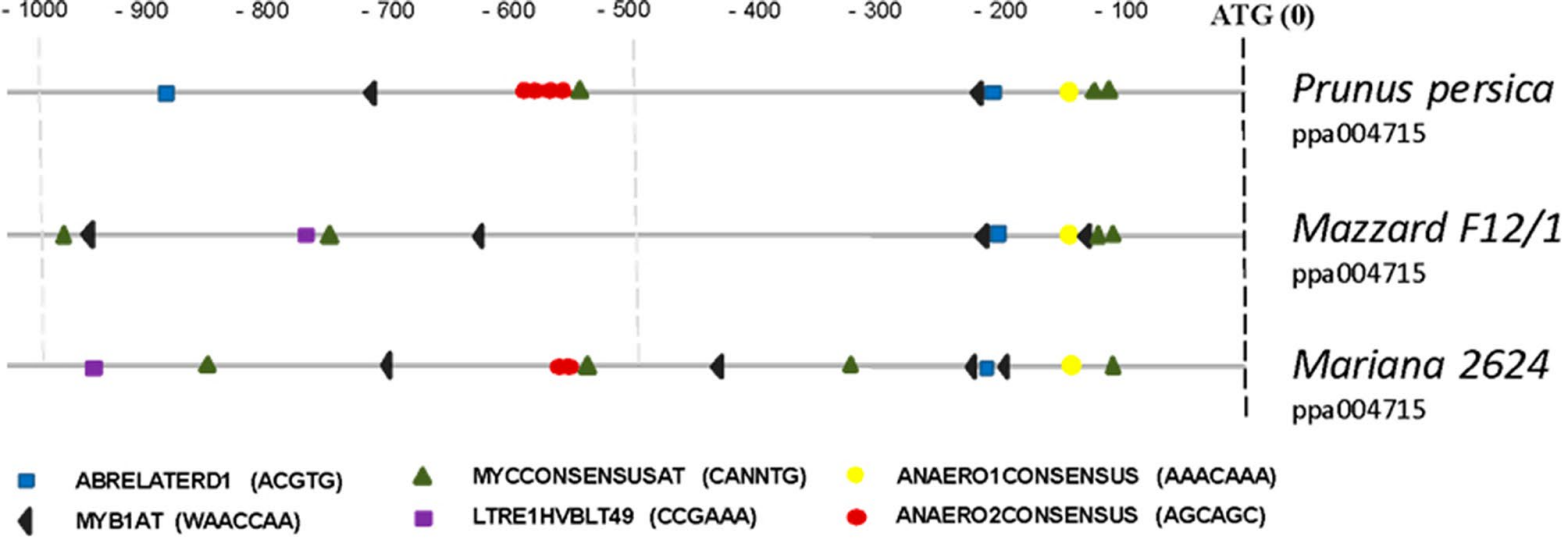
Representation of putative regulatory elements in *Prunus HXK3* promoters. In silico analysis was performed using the PLACE (http://www.dna.affrc.go.jp/PLACE) and PlantCARE databases (http://bioinformatics.psb.ugent.be/webtools/plantcare/html).

In order to give lights about the differential expression of *HXK3* genes in the *Prunus* rootstocks under study, we isolated, cloned and sequenced the ca. 1000 bp gene promoter region of *HXK3* from ‘M.F12/1’ and ‘M.2624’ genomes. Furthermore, the DNA sequences were also compared with the HXK3 gene promoter sequences from other *Prunus* species whose genomes are already available (Supplementary Fig. S2). The ca. 992 bp upstream regions of the transcription starting sites of the *Prunus HXK3* genes were analyzed in silico using the PLACE and PlantCare databases. The analysis revealed differences in the type, number and position of *cis*-elements among the three promoter regions (Fig. 1). At least six of the motifs found in the promoter are potentially involved in gene responses to hormones and abiotic stresses. The ABRELATERD1 motif (ACGTG), which responds to ABA, dehydration and etiolation, was found in all promoters within the first − 200 bp proximal to the transcription starting site. However, there was an additional element in *P. persica* at − 900 bp. LTR1HVBLT49 (CCGAAA), a low-temperature-responsive element was present in rootstocks ‘M.2624’ and ‘M.F12/1’, but not in *P. persica*. MYB1AT (WAACCA), associated with responses to ABA and dehydration, was present in all the analyzed promoters, but varies from 2 (*P. persica*) to 4 copies (‘M.F12/1’ and ‘M.2624’). Interestingly, three copies of this motif were found within the first − 500 bp of ATG, in the hypoxia tolerant rootstock (‘M.2624’). Motif MYCCONSENSUSAT (CANNTG), which responds to ABA, low temperature and dehydration, was found in the three sequences that were analyzed. It varies from 3 copies in *P. persica* to 4 copies in ‘M.F12/1’ and ‘M.2624’. Finally, the putative promoter regions also harbor two important elements or motifs: ANAERO1CONSENSUS (AAACAAA) and ANAERO2CONSENSUS (AGCAGC), which are found in promoters of anaerobic genes involved in the fermentative pathway. ANAERO1CONSENSUS is found in all the analyzed promoter regions (~ 150 bp from the transcription starting site), but ANAERO2CONSENSUS is only present in the promoters of ‘M.2624’, *P. persica*, *P. mume*, *P. dulcis* and *P. armeniaca* (Supplementary Fig. S2). Interestingly, this motif is absent in the ‘M.F12/1’ rootstock (hypoxia sensitive), and the other *P. avium* genotypes (Supplementary Fig. S2) which strongly suggests an important role in differential expression of these genes to root hypoxia.

### Cloning and sequence analysis of *Prunus HXK3*

**Figure 2:**
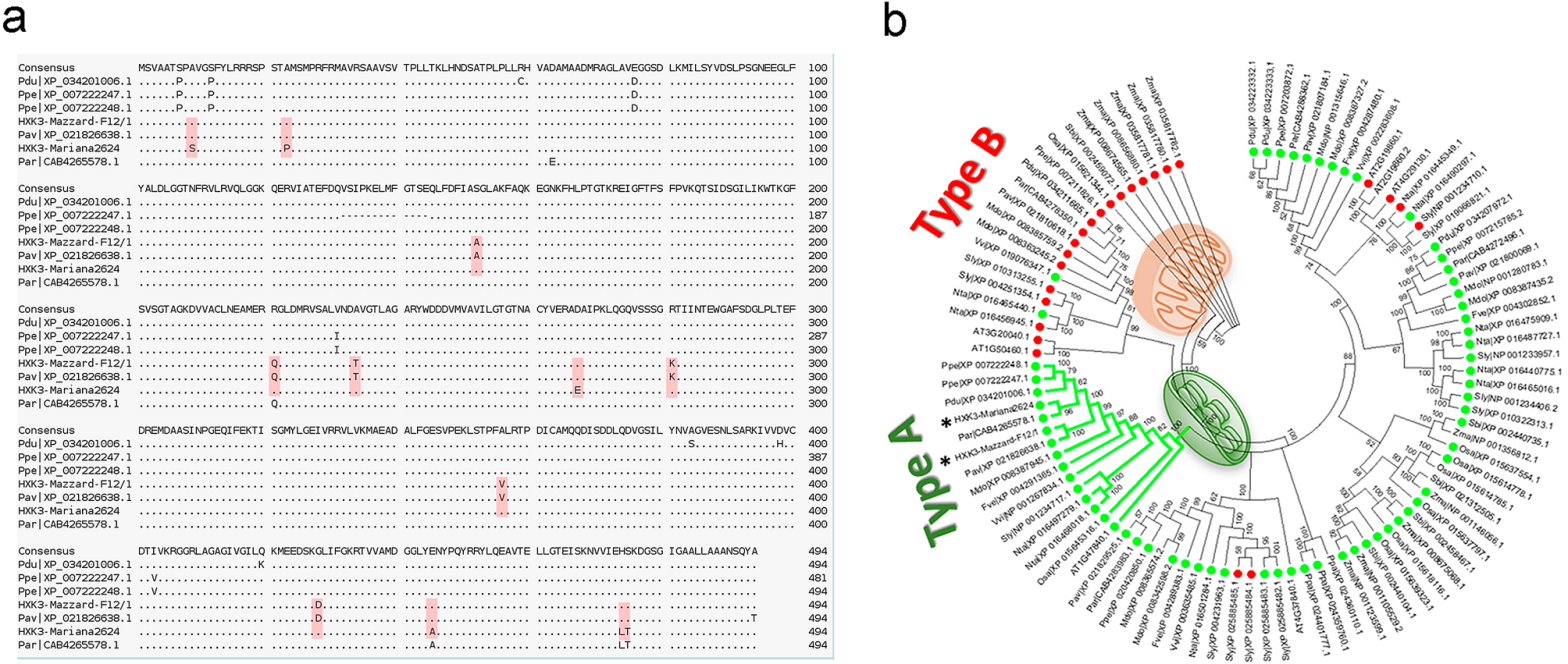
Identification of two putative *Prunus* HXK3 enzymes. (**a**) Comparison of predicted amino acid sequences of ‘M.2624’ HXK3 and ‘M.F12/1’ HXK3 with homologs from Prunus species. Differences at the amino acids level between proteins are enclosed in red squares. (**b**) Phylogenetic relationship among HXK proteins. The phylogram was generated with the MEGA 6.0 program from the multiple alignments of the deduced amino acid sequences from Prunus HXK3 and other angiosperm HXK proteins. Bootstrap values from 1000 replicates were used to assess the robustness of the tree. Green (chloroplast/plastid) and red (mitochondria) dots indicate the putative subcellular localization of HXK proteins (Plant-mSubP, http://bioinfo.usu.edu/Plant-mSubP/). The types of plant HXK are indicated. The positions of ‘M.2624’ and ‘F.12/1’ HXKs are indicated by asterisks. Gene identification are: *AT, Arabidopsis thalian*a; *Fve, Fragaria vesca* subsp. *vesca*; *Mdo, Malus domestica*; *Nta, Nicotiana tabacum*; *Osa, Oryza sativa Japonica* Group; *Ppa, Physcomitrella patens*; *Par, Prunus armeniaca*; *Pav, Prunus avium*; *Pdu, Prunus dulcis*; *Ppe, Prunus persica*; *Sly, Solanum lycopersicum*; *Sbi, Sorghum bicolor*; *Vvi, Vitis vinifera*; *Zma, Zea mays*.

Two previously uncharacterized HXK3 cDNAs, *P. cerasifera* × *P. munsoniana* HXK3 (Pc × Pm HXK3, GenBank accession no. 2400786) and *P. avium* HXK3 (Pa HXK3, GenBank accession no. XM_021970946.1), were isolated from ‘M.2624’ and ‘M.F12/1’ rootstocks, respectively. Both *HXK3* genes show an open reading frame of 1485 bp and are predicted to encode a protein of 494 amino acid residues (Fig. 2a). When the *Pc* × *Pm* HXK3 and *Pa* HXK3 amino acid sequences were compared with sequences from the GenBank database, significant similarities with other HXK3 proteins of both woody and herbaceous plants were found. Also, when compared with other *Prunus* species a high degree of conservation in protein sequences was observed and they only differed in 12 residues (Fig. 2a, red squares). The *P. persica* HXK3 shared 97.7% identity with *Pc* × *Pm* HXK3 and *Pa* HXK3 proteins, and there was 97.57% identity between ‘M.F12/1’ and ‘M.2624’ HXK3. The phylogenetic tree (Fig. 2b) shows that the two HXK3s isolated from the rootstocks are grouped into the same cluster as other plastidial HXKs such as AtHXK3 (AT1G47840.1), SlHXK4 (Sly_NP_001234717.1), NtHXK2 (Nta_XP_016468018.1), OsHXK4 (Osa_XP_015645316.1) and VvHXK2 (Vvi_NP_001267834.1). The structure of Arabidopsis AtHXK1 was used as a template for modeling the deduced HXK3 proteins of the two rootstocks (‘M.2624’ and ‘M.F12/1’) and the reported *P. persica* HXK3. No significant differences were observed comparing their 3D protein structures (Supplementary Fig. S3).

### *Prunus* HXK3 proteins are associated with the chloroplast/plastid

**Figure 3:**
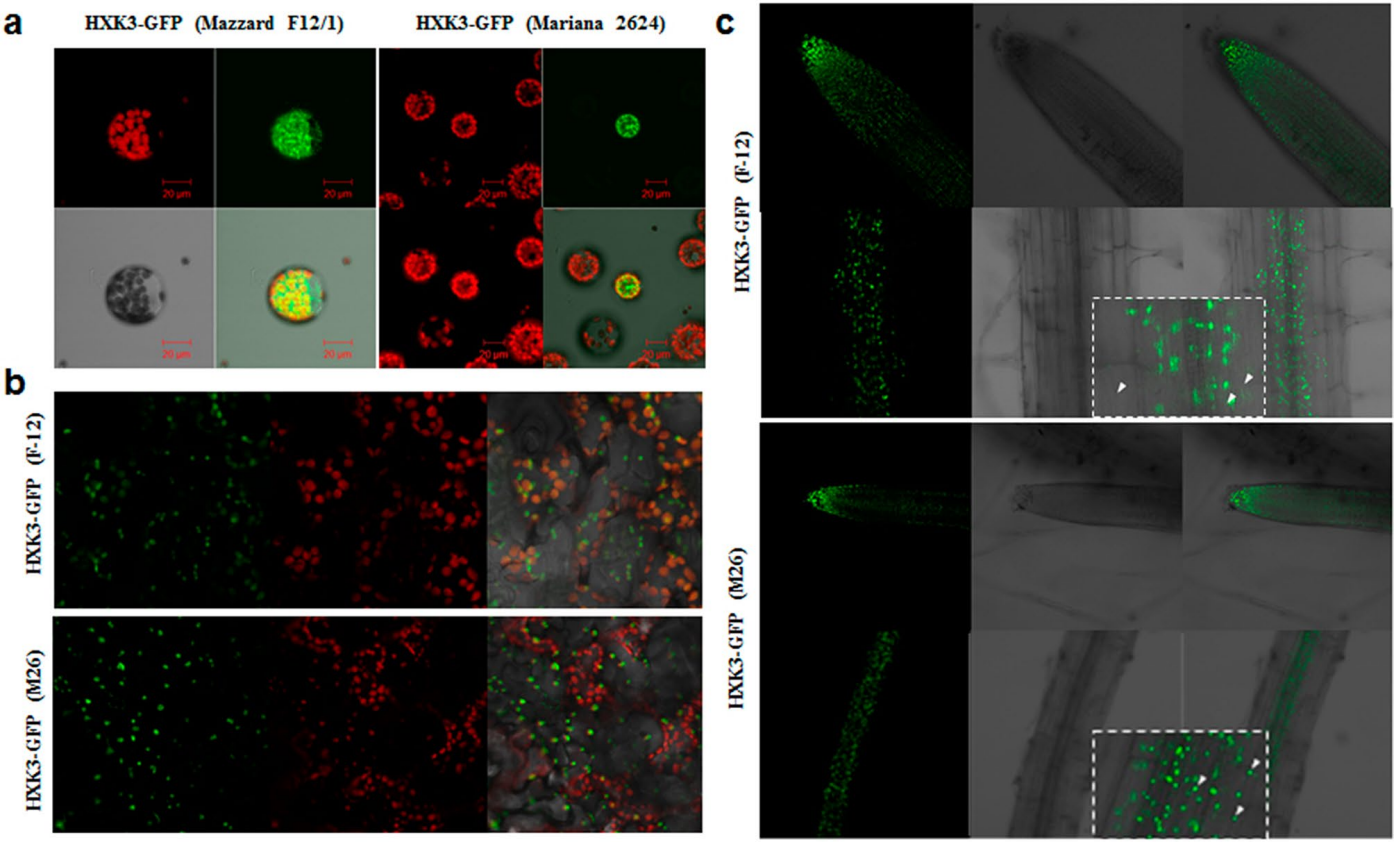
Subcellular localization of *Prunus* HXK3-GFP proteins. (**a**) Transient expression in *Arabidopsis thaliana* protoplasts. (**b**) Stable expression in Arabidopsis leaves. (**c**) Stable expression in Arabidopsis roots. Cells rich in chloroplast as epidermal pavement and mesophyll cells were detected by chlorophyll fluorescence (red) and HXK3-GFP was detected by green fluorescence. After transformation, the samples were incubated for 24 h at 22 °C (Arabidopsis protoplasts). The samples were visualized by confocal microscope. The experiments were done in triplicate, resulting with the same fluorescence patterns.

Subcellular localization of *Prunus* HXKs was bioinformatically predicted with the TargetP-2.0 server, which showed a higher score (0.974 and 0.972) for chloroplast localization (Type A) (Supplementary Table S1). The experimental subcellular localization of the *Prunus* HXK3 proteins by means of transient and stable expression of this protein fused to the green fluorescent protein (GFP) in Arabidopsis protoplasts and transgenic plants showed only plastidial localization (Fig. 3). It has been reported that AtHXK3 is localized in plastids, so chlorophyll fluorescence was used as the control. *Prunus* HXK3 expression in Arabidopsis protoplasts (Fig. 3a) and leaf cells (Fig. 3b) is strongly associated with chloroplasts, while proteins in the roots of both rootstocks are strongly associated with structures that likely are plastids (Fig. 3c).

### Ectopic expression of *Prunus HXK3* genes in Arabidopsis enhances tolerance to salt and drought stress

**Figure 4:**
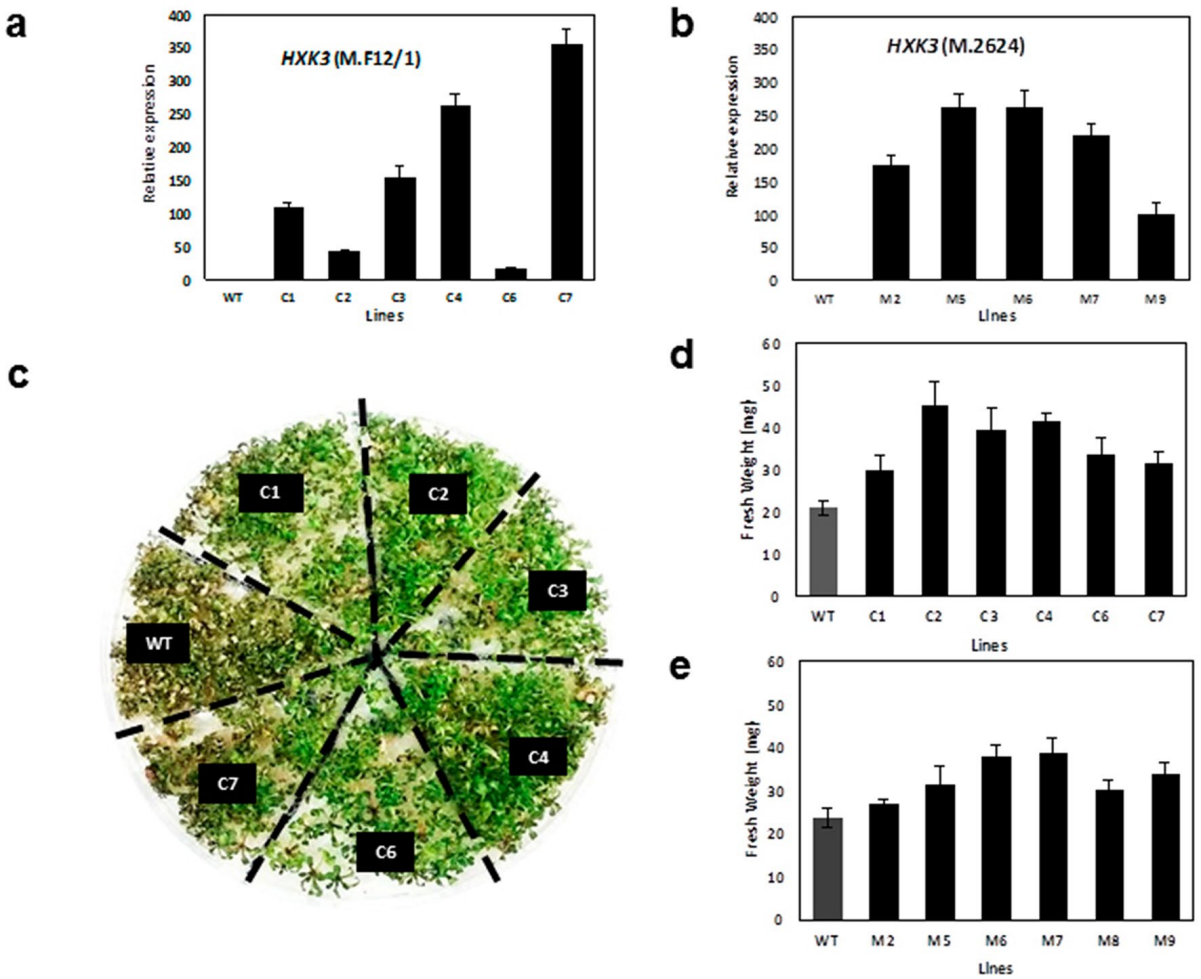
*Prunus HXK3* expression affects salt tolerance in *Arabidopsis thaliana.* Fresh weight of WT and transgenic lines grown after 2 weeks with 50 mM NaCl. (**a**) Relative expression of *Prunus HXK3* (from ‘M.F12/1’) in transgenic lines of *A. thaliana*. (**b**) Relative expression of *Prunus HXK3* (from ‘M.2624’) in transgenic lines of *A. thaliana*. (**c**) Images show representative plants at the end of experiment. (**d**) Fresh weight of WT and transgenic lines (expressing *HXK3* from ‘M.F12/1’) grown after 2 weeks with 50 mM NaCl. (**e**) Fresh weight of WT and transgenic lines (expressing HXK3 from ‘M.2624’) grown after 2 weeks with 50 mM NaC (mean ± se, n = 24).

To determine whether *HXK3* genes provide abiotic stress tolerance, Arabidopsis plants ectopically expressing the *Prunus HXK3* were generated and their performance analyzed under abiotic stress growing conditions (e.g. hypoxia, drought and salinity stress). Although HXK3 proteins were almost identical in sequence and structure, some amino acids differences we evidenced and therefore, we generated T3 homozygous Arabidopsis transgenic plants for the *HKX3-like* genes from ‘M.2624’ (six lines) and ‘M.F12/1’ (five lines) rootstocks*,* respectively (Fig. 4a,b). Compared to the wild-type (WT), the transgenic lines did not show any evident morphological differences in vegetative or reproductive organs during their development under optimal conditions (16 h photoperiod and 23 °C). The *Prunus HKX3* transgenic lines were subjected to hypoxia treatment by waterlogging. After three weeks the transgenic plants did not show evident phenotype differences from the WT under our experimental conditions (data not shown). This result suggests that our experimental conditions were not severe enough to differentiate between genotypes. The effects of *Prunus HXK3* expression on other abiotic stress conditions such as salinity was studied in an in vitro assay using a MS medium containing 50 mM NaCl. After 2 weeks under salt stress, the WT plants clearly had slower growth than the transgenic lines, as well as higher chlorophyll loss and seedling death (Fig. 4c). Fresh weight measurements showed that both groups of transgenic lines had significantly higher biomass (50% more in some lines) than the WT (Fig. 4d,e).

**Figure 5:**
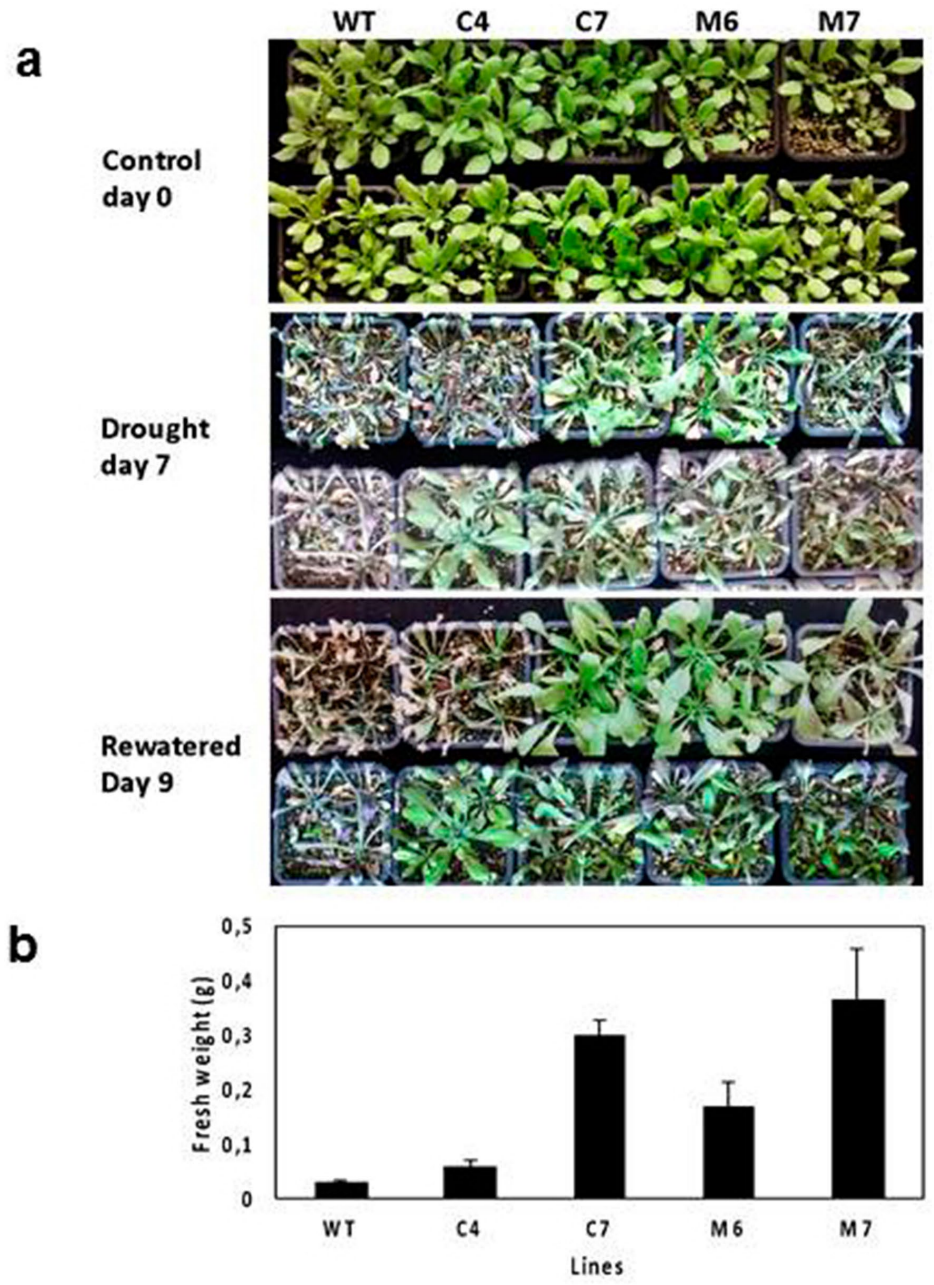
*Prunus HXK3* expression affects drought tolerance in *Arabidopsis thaliana*. (**a**) Image of representative 4-week-old wild-type and transgenic Arabidopsis plants before drought treatment (upper photo), after withholding water for 7 days, and one day after recovery (lower photo). (**b**) Fresh weight of wild-type and transgenic lines grown after recovery (mean ± se, n = 24).

We selected two lines of each group (‘M.F12/1’ and ‘M.2624’) based on their levels of expression (Fig. 4a,b) to determine the performance of *HXK3* transgenic lines under drought stress by water deprivation. These lines and the WT were tested during the vegetative stage. Four-week-old plants in pots were subjected to water deprivation for seven days, at which point all plants showed signs of severe leaf dehydration (Fig. 5a; middle panel). However, the WT plants were more severely damaged than the transgenic plants. After rewatering, almost all transgenic lines recovered to a full or partial green stage. In contrast, WT plants were drastically affected, with most dying and only few plants showed a partial recovery. Whole plant biomass (fresh weight) of transgenic lines were significantly higher than that of the WT plants after re-watering (day nine; Fig. 5b). Overall, these experiments indicate that the *Prunus HXK3* expression enhances drought and salt stress tolerance in Arabidopsis.

### Ectopic expression of *Prunus HXK3* in Arabidopsis affects primary metabolism

**Figure 6:**
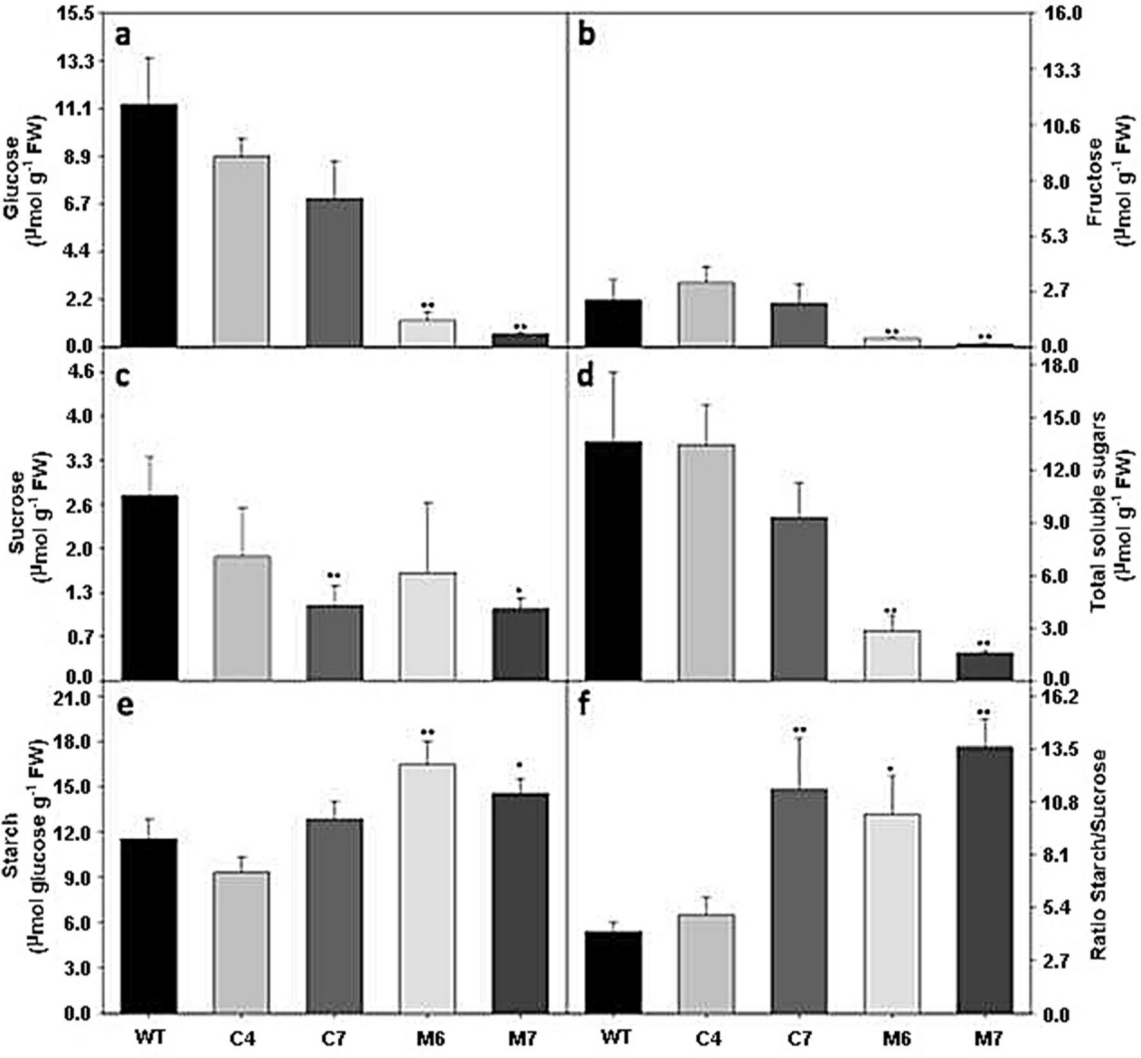
Carbohydrates content in *HXK3* transgenic lines of *Arabidopsis thaliana* plant growing under optimal condition. (**a**) Glucose, (**b**) Fructose, (**c**) Sucrose, (**d**) Total soluble sugar, (**e**) Starch and (**f**) ratio Starch/Sucrose content in *A. thaliana* leaves. Values are presented as means ± SE (n = 5). Asterisks indicate that the values from lines were determined to be significantly different (**P < 0.01 and *P < 0.05) from the wild-type.

HXK is a glycolytic enzyme and hence, contributes to breakdown of carbohydrates to fuel respiration and provides carbon intermediates to numerous anabolic pathways^26^. To determine the effect(s) of the ectopic expression of *Prunus HXK3* on primary metabolism, the leaves from Arabidopsis transgenic plants growing under optimal environmental conditions (16 h photoperiod and 22 °C) were analyzed by HPLC-GC–MS. Starch, soluble sugars (Glc, Fru and sucrose), total free amino acids, organic acids (malate and fumarate) and nitrate were measured. The plants expressing *Prunus HXK3* genes presented significantly lower levels of soluble sugars, with higher intensity in lines M6 and M7, where reducing sugars like Glc and Fru were significantly lower compared to their respective WT (Fig. 6a,b). Sucrose levels changed only slightly in all transgenic lines (Fig. 6c). Overall, starch accumulation increased at midday in plants with higher *HXK3 gene* expression (Fig. 6e), resulting in an increased starch/sugar ratio (Fig. 6f). Notably, organic acid (OA) metabolism seems to be highly affected by *HXK3* expression in *A. thaliana*. Malate and fumarate increased significantly in all transgenic lines (Supplementary Fig. S4). Total amino acid content decreased in all transgenic lines (Supplementary Fig. S5).

**Figure 7:**
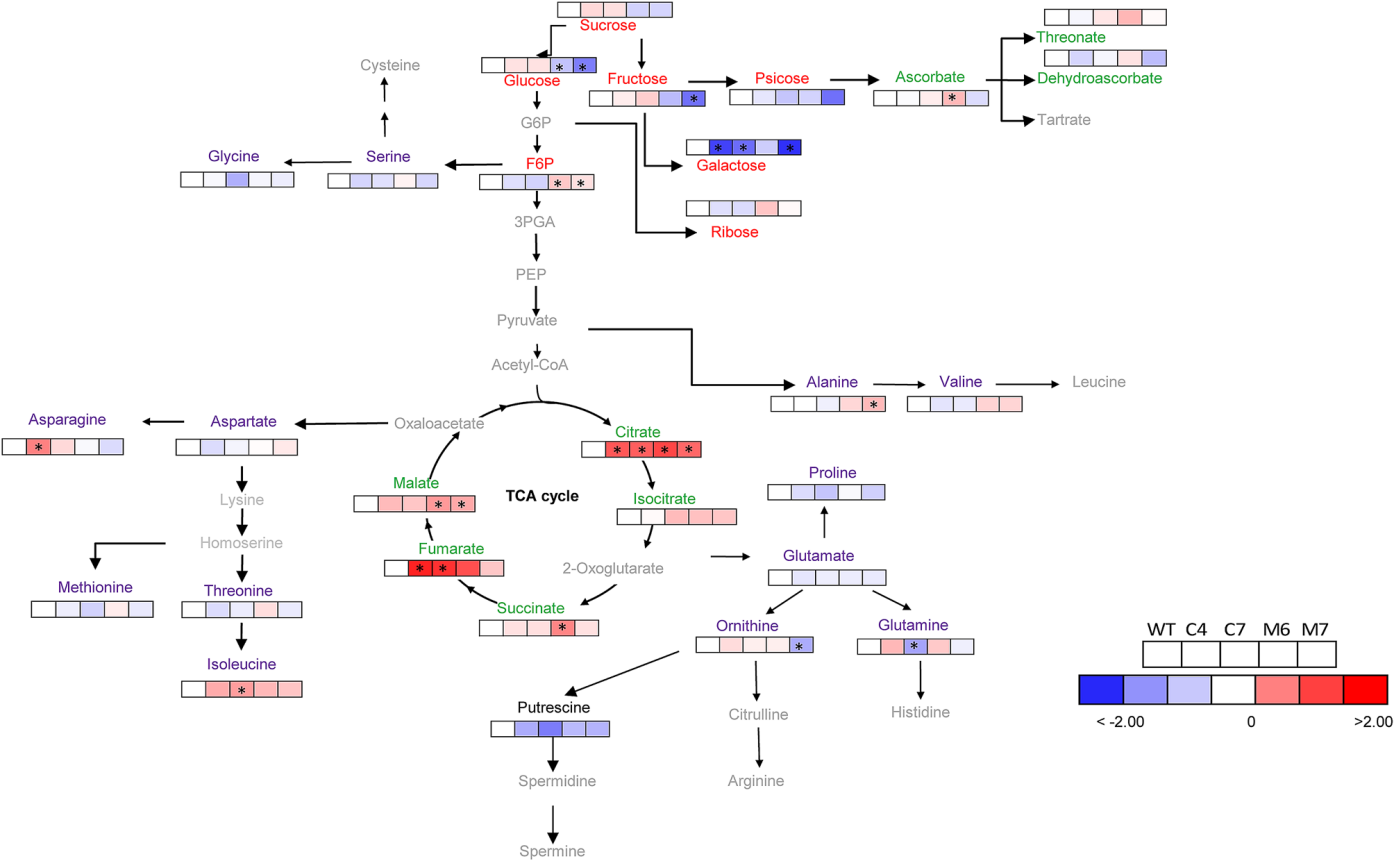
Representative metabolites pathway changes in *HXK3* transgenic lines of *Arabidopsis thaliana* plant growing under optimal condition. Amino acids, organic acids, sugars, and sugar alcohols were determined by GC/MS as described in M&M section. The full data sets from these metabolic profiling studies are additionally available in Supplementary Table S2. Data is normalized with its respective wild-type (Columbia-0) (to allow statistical assessment, individual plants from this set were normalized in the same way). The color code of the heat map is given at the log2 following the scale above the diagram. Different colors represent levels of metabolite fold change where red is increasing and blue is decreasing. Values are presented as means ± SE (n = 5). Asterisks indicate that the values from transgenic lines were determined by Student’s t test to be significantly different (*P* < 0.05) from the wild type.

Gas chromatography-mass spectrometry (GC–MS)^27^ revealed that only 18 of the 44 annotated compounds related to primary metabolism showed significant alteration in comparison to their respective WT (Fig. 7, Supplementary Table S2). A total of 14 amino acids were identified in all overexpressed *HXK3* lines under optimal growing conditions, with major changes in alanine, asparagine, glutamine, isoleucine and ornithine (Fig. 7, Supplementary Table S2). Organic acid levels varied among genotypes with significant changes in ascorbate, citrate, fumarate, malate and succinate (Fig. 7, Supplementary Table S2). Under optimal growing conditions, the ectopic expression of *HXK3* genes in *A. thaliana* has an important effect on the TCA cycle. Finally, 15 different sugars compounds, including alcohols and non-alcohols, were identified (Fig. 7 and Supplementary Table S2). However, as noted above, the main alterations induced by *Prunus HXK3* expression were observed only in reducing sugars, such as Glc, Fru and phosphate sugar (fructose-6-phosphate). The M6 and M7 lines significantly increased the levels of allose and galactose. To determine if the expression of *Prunus HXK3* genes in Arabidopsis is associated with the increase of phosphorylated metabolites and glycolytic intermediates, analysis of some of them was performed (Supplementary Fig. S6). The increase of glucose-6-phosphate (G6P) in all transgenic lines (Supplementary Fig. S6a) indicates that these Prunus hexokinases are functionally active.

## Discussion

Plants need sugar, sugar-sensing and signaling mechanisms to regulate important functions like photosynthesis, carbon metabolism, growth, senescence and response to biotic and abiotic stresses^2^. In this sense, hexose-phosphorylating enzymes, such as HXKs, play an essential role in controlling these processes. Previously, we reported that five out the seven *HXK-*like genes annotated in the *Prunus persica* genome were differentially expressed in roots of flooded plants from two *Prunus* rootstocks (‘M.2624’ and ‘M.F12/1’) ^23^. So far, the *HXK3* (ppa004715) was the most strongly up-regulated *HXK*-like gene in roots of the tolerant genotype but not in the sensitive genotype^23^ and thus *HXK3* emerged as a candidate gene for further research. In this research, we deeply analyzed the effect(s) of root hypoxia on *HXK3* expression (1, 3, 6, 12 and 24 h; 3 and 7 days of stress) and the function of *Prunus HXK3*-like genes through the metabolic and physiological study of transgenic plants that ectopically express the genes under study. The root hypoxia “boosted” the *HXK3* gene expression very early in ‘M.2624’ but not in ‘M.F12/1’ suggesting a role in the early response of roots to oxygen deprivation. The early response of the *HXK3* gene expression in the tolerant genotype suggests the existence of an early signal sensing mechanism(s) in roots, an adaptive mechanism of hypoxia stress that is clearly not present in the sensitive genotype. In ‘M.2624’, the *HXK3* expression showed a steady increase until 24 h of root hypoxia but also their transcription was relatively higher than in ‘M.F12/1’ inclusive after 7 days of flooding suggesting a role for this gene in adaptive response to long-term hypoxia.

When plant roots are exposed to hypoxic/anoxic conditions, aerobic respiration is inhibited, thus yielding low energy since oxidative phosphorylation is blocked, and ATP is generated by cytosolic glycolysis^28^. HXK enzymes catalyze irreversible reactions in plants providing the initial substrates to generate energy through the glycolytic pathway^5^. Therefore, the higher expression of ‘M.2624’ *HXK3* reported in this work could be important “to fuel” the glycolysis during the first week of waterlogging and to provide energy in the hypoxia-tolerant genotype. Previous studies carried out by our group^29^, showed a similar expression pattern for Class 1 non symbiotic hemoglobin-like (nsHb) gene in the hypoxia-tolerant rootstock ‘M.2624’. The non-symbiotic hemoglobin/nitric oxide cycle has been proposed as an alternative to alcohol dehydrogenase (ADH) in oxidizing NADH^30^. We also observed a decrease in the carbon dioxide assimilation rates (A) and in total chlorophyll concentration due to waterlogging treatment which could contribute to reduce the photosynthesis and decrease the photoassimilates available in root hypoxia stressed plants^23,31^. These results suggest that *HXK3,* together a nsHb/NO cycle, could be operating in roots of hypoxia-tolerant *Prunus* species under oxygen deprivation and thus contribute to maintaining an adequate energy and redox balance in plant cells, at least during the first week of waterlogging. This could explain the energy complement in ‘M.2624’ that allows it to maintain root relative growth rate under oxygen deficiency^25^. The decrease in ‘M.2624’ *HKX3* expression after 24 h of root hypoxia could avoid the sugar starvation until morpho-physiological adaptive mechanisms (the increase in root porosity, the development of lenticels on stems and the generation of adventitious roots with aerenchyma)^25,31^ emerge allowing the diffusion of oxygen to tissues with high demand.

In order to gain detailed insights in *HXK3* regulation, we isolated and sequenced two novel *HXK3* gene promoters from M2624 and Mazzard F12-1 genomes and we compared their sequences with several *HXK3* promoter sequences from other species of the *Prunus* genus (Supplementary Fig. S2). The conservation of *cis*-acting elements such as “response to anaerobiosis”, “response to ABA”, “low temperatures” and “dehydration” in the gene promoters analyzed suggest a role of *HXK3*-like gene(s) in the response(s) of *Prunus* species to hypoxia but also to different abiotic stresses. In the roots of *Arabidopsis thaliana*, *AtHXK2* can be induced by cold stress and salt stress^32^. In apples, *MdHXK1* gene expression was also found to be induced by low temperature and salt stress^33^. Furthermore, in the promoter region of *MdHXK1* gene from ‘Gala’ apple (*Malus* × *domestica* Borkh.) were identified many stress resistance-related cis-acting elements, and the expression level was also induced by NaCl, low temperature (− 10 °C) and 100 µmol/L ABA^33^. This information suggests a key role of the HXK gene family in the adaptive response(s) of plants to abiotic stresses. Outstandingly, the *HXK3* promoter from ‘M.F12/1’ (*P. avium*) genotype has an apparent deletion since it lacks the ANAERO2CONSENSUS (AGCAGC) element, which is a *cis*-acting element present in anaerobic genes involved in the fermentative pathway^34^ (Fig. 1). This *cis*-acting element is also absent in the gene promoter of *HXK3*s from sweet cherry genotypes (sweet cherry varieties) whose genome information is available. This could explain the lower expression of the *HXK3* gene in ‘M.F12/1’ than in ‘M.2624’. It is tempting to hypothesize that the higher sensitivity of *P. avium* (cherry plants in general; see Supplementary Fig. S2) to root hypoxia generated by flooding could be consequence, in part, of the lower expression of *HXK3* gene(s) and this could have basis in the absent of hypoxia-related *cis*-acting element in their gene promoter. Whether differences in the gene promoter architecture of *HXK3* in *Prunus* species have contributed to the tolerance/sensitivity to root hypoxia during the evolution of this group of perennial woody plants is still a question that deserves attention. Furthermore, gene expression experiments of *HXK3* in *Prunus* plants grown under abiotic stresses other than hypoxia should be performed to reinforce its role in the response(s) of Prunus rootstock to other environmental stresses.

We identified novel members of the HXK gene family in the *Prunus* species. The ‘M.2624’ *HXK3* and ‘M.F12/1’ *HXK3* are the first hexokinase-like genes which have been isolated and characterized from *Prunus* species used as stone fruit rootstocks. The deduced amino acid sequences of the HXK3 proteins from ‘M.2624’ and ‘M.F12/1’ rootstocks seems to be well conserved within the *Prunus* genus since a higher amino acid identity and similar tridimensional structures were observed (around 97.77% of amino acid identity between stone fruit HXK3 proteins was found). Furthermore, ‘M.2624’ and ‘M.F12/1’ HXK3s have the characteristic features of Type A HXKs^8^ because they present a chloroplast transit peptide of about 30 amino acids. The putative HXK3 proteins from ‘M.2624’ and ‘M.F12/1’ rootstocks were clustered with AtHXK3, SlHXK4, NtHXK2, OsHXK4 and VvHXK2 (Fig. 2b), a group of proteins localized in the plastid stroma^6,26,35,36^. The same occurs with OsHXK4, which is located in the chloroplast stroma^37^. Based on transient expression experiments in leaf protoplasts and stable expression in Arabidopsis plants, we show that the *Prunus* HXK3 proteins seem to play their function(s) in chloroplast/plastid.

The high homology at both regulatory and protein coding regions, but ‘M.F12/1’ lacks the cis-regulatory element ANAERO2CONSENSUS (AGCAGC), indicate that both HXK3 analyzed are homologous genes, which are expressed in root of Prunus rootstock, both ‘M.2624’ and ‘M.F12/1’. Our experiments of ectopic expression of these genes in Arabidopsis that showed similar pattern of localization and metabolic profile strongly suggest they play similar function(s) in root plastids. However ‘M.F12/1’ does not present the same response to hypoxia stress probably because of the absence of the ANAERO2CONSENSUS cis-regulatory element.

The constitutive overexpression of *AtHXK1* in Arabidopsis and tomato inhibits growth and photosynthesis^12,38,39^. In our study, we also used a constitutive promoter but did not observe any effects on phenotypes during plant development. This is consistent with alterations in metabolite profiles (Fig. 7). We show that expression of *Prunus HXK3* in Arabidopsis plants perturb carbon and energy metabolism promoting the accumulation of starch and OAs at midday under optimal growing conditions. The analysis of phosphorylated metabolites in Arabidopsis leaves (Supplementary Fig. S6) revealed that all transgenic plant lines presented high levels of G6P, indicating that both HXK3 from *Prunus* are able to phosphorylate glucose. Interesting, the four transgenic lines showed similar increment of content of G6P, suggesting that there is a tight control of maximum of G6P produced, which is independent from the amount of *HXK3* transcript or availability of substrates. Moreover, analysis of other phosphorylated metabolites (G1P, UDPG, ADPG, Pyruvate, phosphoenolpyruvate and 3-phosphoglycerate) that not presented a clear pattern of modification, suggest that accumulation of OA levels and starch are not a direct consequence of increment on enzymatic HXK3 activity, but maybe they are products of changes in sugar signaling. (Fig. 7; Supplementary Figs. S4, S6). Although the activity of chloroplastic HXK has been suggested to be a key component for providing G6P to starch and fatty acid synthesis by some authors^35,36^.

The components of TCA (e.g. fumaric acid) and starch can be metabolized to produce energy and/or carbon skeleton for production of other compounds^40^. Starch is emerging as a key molecule in mediating plant responses to abiotic stresses (e.g. drought, high salinity and extreme temperatures). Under environmental stress the photosynthesis could be limited and the remobilization of starch by plants could be a way to obtain energy, sugars and derived metabolites to help mitigate the stress and to increase plant fitness^41^. Several studies have demonstrated that leaf starch is degraded and soluble sugars (e.g. glucose and fructose) unequivocally accumulate in response to stress both in herbaceous (e.g. *Hordeum vulgare*) and woody angiosperms (e.g. litchi tree, *Litchi chinensis*)^42^. Also, degradation of starch in response to stress often has been correlated with improved tolerance in the moss *Physcomitrella patens* and in legumes (broad bean and soybean)^43^. The HXK enzymes catalyze irreversible reactions in plants providing the initial substrates to generate energy through the glycolytic pathway^5^. The tabacco NtHXK2, a Type-A HXKs expressed in starch-containing tissues, would play a role in the phosphorylation of hexoses released from starch breakdown in plastids^35,36^. Previous research have proposed that glycolysis does have the potential to produce ATP at a faster rate when reserves are adequate, and it does not lead to the potential production of highly reactive species, which may be favourable for cell proliferation and survival under challenging environments (reviewed in Ref.^44^).

Taking into account our results and the information mentioned it is tempting to speculate that the over-accumulated starch in Arabidopsis plants that ectopically express *Prunus HXK3* could be degraded to hexoses under drought and salt stresses and these sugars could be quickly phosphorylated by HXK3. The G6P could be employed by plant cells for producing energy through glycolysis and for synthesizing compatible solutes to improve plant survival. The OAs also could contribute to the energy and carbón skeleton supply to mitigate the abiotic stresses. Unfortunately, the hypoxia stress caused by our waterlogging experiment was not severe enough to discriminate at naked eye between transgenic and WT plants. Therefore, we cannot discard or propose a role of C-metabolism changes induced by HXK3 in the tolerance/sensitivity of Arabidopsis plants to oxygen deprivation. Furthermore, we do not discard other rol(es) of *Prunus HXK3* in the regulation of other physiological processes like stomatal closure and photosynthesis in leaves and mineral uptake in roots^5,45^ as have been proposed in other plant species^46^. The availability of transgenic Arabidopsis plants with different *Prunus* hexokinase activity provides a means to study the potential role of these stone fruit tree enzymes in plant development and cell signalling networks. Finally, *Prunus HXK3* emerges as a potential candidate gene for enhancing tolerance to abiotic stresses in stone fruit trees and other crops via genetic breeding.

## Material and methods

### Plant material

Clonally propagated and virus-free rootstocks, Mariana 2624 (*P. cerasifera* × *P. munsoniana)* and Mazzard F12/1 (*P. avium*), which are respectively tolerant and sensitive to hypoxia conditions^23,47–49^, were acquired from a commercial nursery (Agromillora Sur, S.A., Curicó, Chile). The plants were transplanted to 2-L plastic pots with a mixture of vermiculite, perlite and sand (1:1:1v/v) as a substrate. The plants were maintained in the field under a shade net (Raschel sunshading net with 50% light transmittance) at the Instituto de Investigaciones Agropecuarias—Rayentué (Rengo, Chile) during the growing season until the hypoxia experiment. Plants were watered with tap water three times a week and fertilized every 2 weeks with 1 g of N:P:K per pot (25:10:10) (Ultrasol™, Soquimich, Chile). Wild-type (Columbia-0) and transgenic *Arabidopsis thaliana* plants were grown in a greenhouse in a mixture of vermiculite, perlite and peat moss (1:1:1), with a 16 h light/8 h dark photoperiod. Collected samples were frozen with liquid nitrogen and stored at − 80 °C until they were used for RNA isolation.

### Hypoxia treatment with *Prunus* rootstocks

The hypoxia treatment was applied as described by Arismendi et al.^23^. With 24 plants per genotype, roots from three randomly selected plants were collected for RT-qPCR analysis at 0, 1, 3, 6, 12 and 24 h, and then at 3 and 7 days. Samples collected at 0 h represented the control without flooding. Plant roots were gently washed with tap water, then excised from the plants and immediately frozen in liquid nitrogen. They were stored at − 80 °C until RNA extraction.

### In silico analysis of *Prunus* HXK3 promoters

The rootstock genomes were partially sequenced and assembled de novo in our laboratory^50^. We analyzed the promoter regions of *HXK3* for both rootstocks, comparing them to *P. persica, P. avium, P. mume, P. dulcis* and *P. armeniaca* already available at database. The *cis* regulatory elements in promoter regions were searched for using the signal scan search provided by the databases of PLACE (http://www.dna.affrc.go.jp/htdocs/PLACE) and PlantCare (http://www.bioinformatics.psb.ugent.be/webtools/plantcare/html).

### Cloning of Hexokinases genes

Using the sequence information of the hexokinase induced under the hypoxia treatment^23^ (*HXK3*, ppa004715m), available in the phythozome database for *P. persica* v2.1 (https://phytozome.jgi.doe.gov/pz/portal.html) a set of primers were designed containing *XbaI* (PpHXK3-F: 5′-ATCTAGAATGTCAGTTGCTGCCACG-3′) and *SacI* (PpHXK3-R: 5′-TGAGCTCTTATGCATACTGGGAATT-3′) restriction site. cDNAs obtained from roots under hypoxia treatment from ‘M.F12/1’ (*P. avium*) and ‘M.2624’ (*P. cerasifera* × *P. munsoniana*) were used separately to isolate the coding regions of the *HXK3* genes from these genotypes. The coding region of both *HXK3* genes were amplified by PCR using a Platinum^®^ High Fidelity Taq DNA Polymerase (Invitrogen, Waltham, MA, USA), with a set of primers as described above. The PCR products were cloned into the pGEM-T vector (Promega, Madison, WI, USA) and then sequenced (Macrogen, Korea; www.macrogen.com).

### Search for* Prunus HXK* genes and phylogenetic analysis

The nucleotide and deduced amino acid sequences of the rootstock (‘M.2624’ and ‘M.F12/1’) *HXK3* genes were obtained by cloning and sequencing. The amino acid sequence of *P. persica* HXK3 was obtained from the phythozome database (Prupe.1G366000.1-ppa004715m). The deduced amino acid sequence of rootstock HXK3 and other plant HXKs were used as a template for multialignment using BioEdit v7.0 Sequence Alignment Editor Software^51^. The phylogenetic tree was built from the resulting alignment using MEGA software (version 5; http://www.megasoftware.net) and the neighbor-joining method, with bootstrap analysis of 1000 replicates. The UniProt accession numbers of plant hexokinase proteins used for the phylogenetic tree are available as supplementary material (Supplementary Table S3).

### Protein modeling

The hexokinase models and their three-dimensional (3D) structures were constructed according to Xiang^52^. A PSI-Blast of the sequences was performed against the Protein Data Bank (PDB) database to select a template with an identity greater than 30% and with the broadest coverage. The structure of hexokinase 1 of *A. thaliana* (AtHXK1), with the PDB code 4QS8, was used as a template. The MODELLER V9.20 program^53^ was used to construct the hexokinase models. The three-dimensional structures were optimized geometrically by energy minimization. Energy minimization and thermodynamic balancing were performed with NAMD 2.9 software^54^.

### Genetic construction and plant transformation

The coding region of *HXK3* cloned into the pGEM-T vector was subcloned into the pBI121 binary vector to replace the *β-glucoronidase* (*GUS*) gene under the control of the CaMV 35S promoter. Plasmids were introduced into the *Agrobacterium tumefaciens* strain GV3101. *A. thaliana* Columbia-0 was transformed by the floral-dip method^55^. Transgenic Arabidopsis seedlings were selected in MS medium containing 50 mg L^−1^ kanamycin and 250 mg L^−1^ cefotaxime. The kanamycin-resistant plantlets were transferred to a mixture of soil (1:1:1 of vermiculite, perlite and peat moss) and later moved to a greenhouse under controlled conditions. The presence of the transgene was confirmed in transgenic lines by PCR from gDNA using specific primers for *PpHXK3*. Genomic DNA was isolated using the Wizard^®^ Genomic DNA Purification Kit (Promega, Madison, WI, USA). T1 plants were used for molecular and phenotypic analysis and then to generate T3 plants.

### Subcellular localization of the HXK3-GFP fusion protein

The HXK3 coding region was PCR-amplified using a *Pfu* DNA polymerase (Thermo Fisher Scientific Inc.), with the following primer pairs: HXK3GFP-F (5′-CACCATGTCAGTTGCTGCCACG-3′) and HXK3GFP-R (5′-TGCATACTGGGAATTTGCAGCAG-3′). The gene was cloned into the entry vector pENTR™/SD/D-TOPO^®^ (Thermo Fisher Scientific Inc.) to generate pENTR-HXK3 proteins, which was verified by sequencing (Macrogen, Korea; www.macrogen.com). The pENTR-HXK3 protein was recombined by LR Clonase^®^ reaction (Thermo Fisher Scientific Inc.) into the destination vector pGWB505^56^, under the control of the 35S promoter. Protoplasts obtained from 5-week-old *A. thaliana* Col-0 plants by the tape-Arabidopsis sandwich method^57^ were transformed with the plasmid. The transformation was as described in Wu et al.^57^, with 15 min of incubation in PEG solution. The transformed Arabidopsis protoplasts were visualized 24 h after transformation. The plasmid was used to transform *A. tumefaciens* for stable transformation (strain GV3101) and *A. thaliana* was transformed by the floral-dip method^55^. Leaves and roots of homozygous T3 Arabidopsis transgenic plants were collected and visualized. Samples from transient and stable transformation were visualized using a confocal laser scanning microscope (Zeiss LSM510, Germany). Two filter settings were used, one for GFP fluorescence, excitation with 488 nm, and the other for chlorophyll autofluorescence, excitation at 683–747 nm. The images were captured with a confocal microscope and processed with ImageJ. Overlap coefficients were calculated using Zeiss ZEN 2012 software^58^. The complete amino acid sequences (*A. thaliana* AtHXK3; *P. persica* HXK3 and deduced amino acid sequences of the rootstocks ‘M.2624’ and ‘M.F12/1’) were compared using the subcellular prediction localization program TargetP-2.0 (http://www.cbs.dtu.dk/services/TargetP/)^59^.

### RNA isolation and cDNA synthesis

Three biological replicates were used per treatment for gene expression analysis. Total RNA was extracted from root samples of control and flooded Prunus rootstock plants at 0, 1, 3, 6, 12 and 24 h and then at 3 and 7 days of hypoxia, according to Chang et al.^60^. Arabidopsis RNA was obtained with the SV Total RNA Isolation System (Promega, Madison, WI, USA). RNA integrity was determined by visualization with formaldehyde agarose gel electrophoresis, and the concentration and purity (OD_260/280_ ratio > 1.95) were analyzed with an Infinite^®^ 200 PRO NanoQuant (Tecan Group Ltd., Switzerland). All RNA samples were treated with RNase-free DNase I (Ambion, TX, USA) before being reverse transcribed in a 20 μl reaction using the Thermoscript RT-PCR System™ (Invitrogen, Inc., Carlsbad, CA) according to the manufacturer’s instructions.

### Gene expression analyses

Transcript levels of studied genes were measured by RT-qPCR using a Mx3000P QPCR System (Agilent Technologies, Santa Clara, CA). All reactions were conducted using Brilliant SYBR Green Master Mix (Stratagene Inc., Santa Clara, CA), in accordance with the manufacturer’s instructions. All the reactions were carried out in triplicate (technical replicates) using 2 μL Master Mix, 0.5 μL 250 nM of every primer, 1 μL of diluted cDNA and nuclease-free water to a final volume of 20 μL. Controls were included in all runs. Fluorescence was measured at the end of the amplification cycles. Following amplification, a melting curve analysis with continual fluorescence data acquisition was conducted during the 65–95 °C melt. The set of primers for *Prunus* PHXK3 was taken from Arismendi et al.^23^ (qHXK3-F: 5′-GTGGCAATGGATGGAGGGTTGTAT-3′ and qHXK3-R: 5′-CTGCCCTACGACCCATTCTCATTA-3′). The expression of this gene was normalized against the *P. persica* two-gene elongation translation factor (PpaTEF2, GenBank database accession number TC3544) because of its consistent transcript level^61^. The primers to detect *Prunus HXK3* in transgenic Arabidopsis were: qHXK3B-F, 5′-TTTCTGTCACTCCGCTGTTG-3′, and qHXK3B-R: 5′-CGCCTAGTTGTACCCTCAGC-3′. The expression of the transgene was normalized against the AtFbox-Fw, 5′-TTTCGGCTGAGAGGTTCGAGT-3′ and AtFbox-Rw, 5′-GATTCCAAGACGTAAAGC AGATCAA-3′^62^.

### Abiotic stresses in *A. thaliana* expressing *Prunus HXK3* genes

In order to imitate the experimental condition used in the root hypoxia experiment with *Prunus* species, we waterlogged 4-week-old Arabidopsis plants growing in square pots (with a height of 6.5 cm) in a tank to a water depth of 6.5 cm (from the bottom of the pots) and maintained for 3 weeks at 22 °C in a growth chamber. In this way, only the roots were waterlogged. Salt stress in Arabidopsis was applied in an in vitro system. WT and transgenic seedlings were grown in Murashige and Skoog medium (MS) for ten days and then transferred to an MS medium containing 50 mM of NaCl. Seedlings were grown in this medium for two weeks and then collected to determine their biomass. The Arabidopsis drought tolerance test involved 4-week-old plants initially grown under a normal watering regime. Plants were subjected to a drought regime for approximately 7 days until WT plants exhibited the lethal effects of dehydration. Watering was then resumed and plants were allowed to recover for one day. Plants were photographed and collected to determine fresh weight.

### Determination of metabolite levels and biochemical assays

Leaf samples were collected at midday, frozen in liquid nitrogen and stored at − 80 °C until further analysis. Samples were extracted rapidly by grinding tissue in liquid nitrogen and immediately adding extraction buffer. Briefly, the extraction employed 700 µL of methanol (for HPLC > 99.9%), with shaking at 800 rpm at 70 °C for 15 min. 60 µL of ribitol (0.2 mg mL^−1^) was added with the extractor reagent as an internal standard for further GC–MS analyses. After heating, the samples were centrifuged, the supernatant was separated from the pellet, and 375 µL of chloroform (for HPLC > 99.9%) and 750 µL of mili-Q water were added to the supernatant. Samples were centrifuged again and the aqueous phase was separated for future analysis. Citrate, malate and fumarate levels were determined as described by Nunes-Nesi et al.^63^, as well as quantification of starch, glucose, fructose, sucrose^64^, free amino acids^65^ and nitrates were quantified, the latter according to Fritz et al.^66^. The extracted samples were derivatized as described by Roessner et al.^67^ and the metabolite profiles of both tissues were determined by gas chromatography-mass spectrometry (GC–MS), as described by Lisec et al.^27^. Chromatograms were evaluated with TagFinder software^68^, using the reference library available in the Golm Metabolome Database^69^. Metabolites such as ADP-glucose (ADPG), UDP-glucose (UDPG), glucose 1-phosphate (G1P) and glucose 6-phosphate (G6P) were extracted and quantified by LC–MS/MS using a reverse phase^70^. Samples were spiked with stable isotope-labelled standards to correct for ion suppression and other matrix effects^71^. 3PGA, phosphoenolpyruvate (PEP), and pyruvate were measured enzymatically in fresh trichloroacetic acid extracts^72,73^.

## Supplementary Information

Supplementary Information 1.

Supplementary Information 2.

Supplementary Information 3.
