# Supplementary material for: Prunus Hexokinase 3 genes alter primary C-metabolism and promote drought and salt stress tolerance in Arabidopsis transgenic plants
Source: Sci Rep. 2021 Mar 29;11:7098. doi: 10.1038/s41598-021-86535-1 (PMC8007757; doi:10.1038/s41598-021-86535-1)
Supplement: Supplementary file 1 — Supplementary Information 1. [file 41598_2021_86535_MOESM1_ESM.pdf]

Title:

Prunus Hexokinase 3 genes alter primary C-metabolism and promote drought and salt stress tolerance in *Arabidopsis transgenic* plants

Jorge Pérez-Díaz[1], Willian Batista-Silva[2], Rubén Almada[3], David B. Medeiros[2], Stéphanie Arrivault[4], Francisco Correa[1], Adriana Bastías[5], Pamela Rojas[1], María Francisca Beltrán[1], María Francisca Pozo[1], Wagner L. Araújo[2] and Boris Sagredo[1].

[1]Instituto de Investigaciones Agropecuarias CRI Rayentué, Av. Salamanca s/n, Sector Los Choapinos, Rengo, Chile
[2]Max Planck Partner Group at the Departamento de Biologia Vegetal, Universidade Federal de Viçosa, 36570-900, Viçosa-MG, Brazil
[3]Centro de Estudios Avanzados en Fruticultura, CEAF, Camino Las Parcelas 882, Sector Los Choapinos, Rengo, Chile
[4]Max Planck Institute of Molecular Plant Physiology, Am Mühlenberg 1, D-14476 Potsdam-Golm, Germany
[5]Universidad Autónoma de Chile. Av. Pedro de Valdivia 425, Providencia, Santiago, Chile

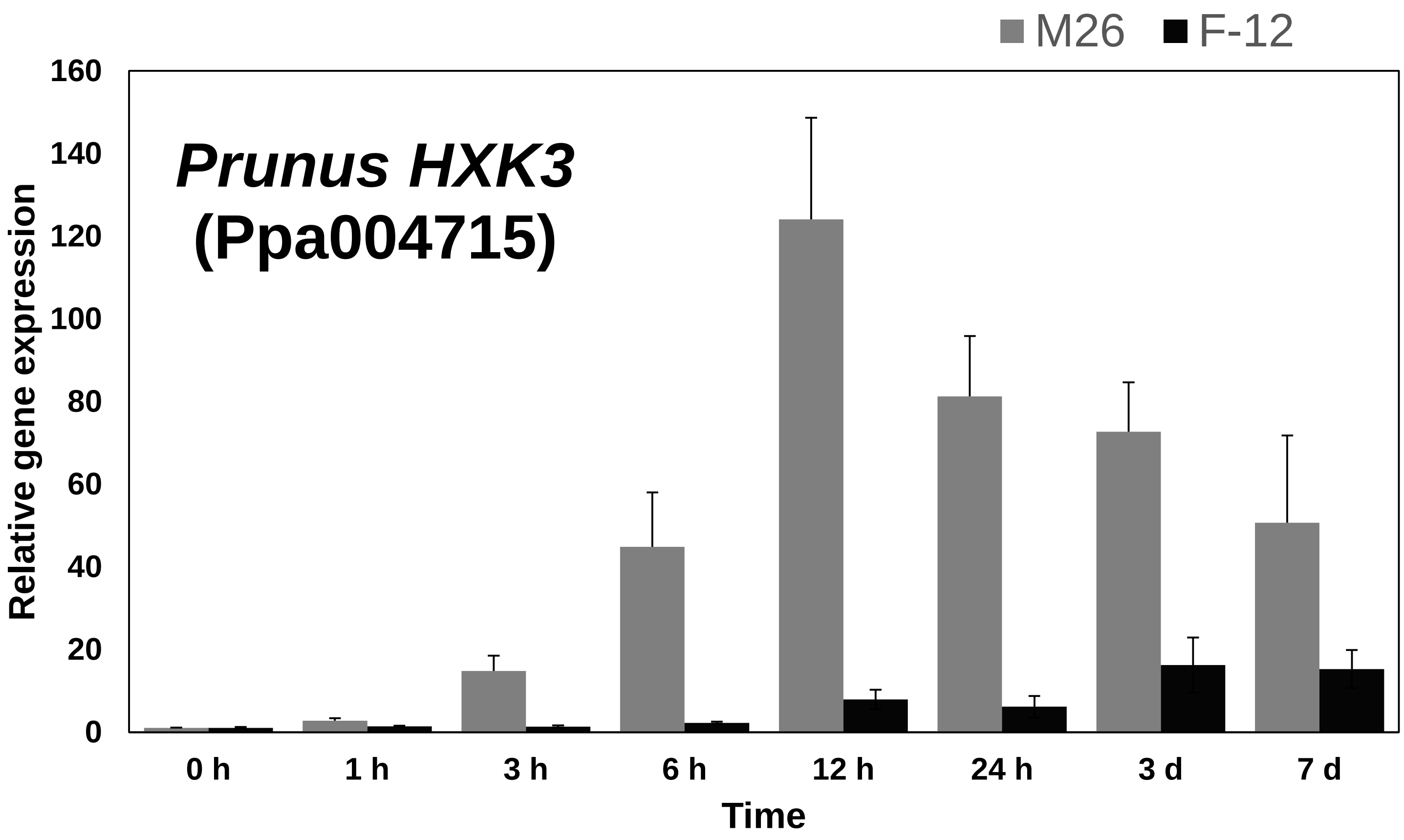

Figure S1. Expression patterns of the *HXK3* genes in *Prunus* plants subjected to hypoxia. Gene expression was examined by qRT-PCR using total RNA from rootstock roots of Mariana 2624 and Mazzard F12/1 after treatment with hypoxia for 0, 1, 3, 6, 12, 24, 72 and 168 h. They are expressed as mean relative expression values +/− standard error (n=3), normalized with *TEF2* as a constitutively expressed gene.

>Prunus_persica|Prupe.1G366000 *HXK3* promoter

AAAAAGTAAAATTAAAAATAAAATCAAATAATAGGTCATACTTTTATTAAACATAACTATCCATATTATTTTTTCTTAATTCAGAAAAAAAAAATCAATTAGCACATA
CGTGAGTATGTTCCAAGGGGCTTGTGGTACAGAAAGAGCAATGGTATGTATGGCATACCGTTTCTATCAAGTTCCCAAAGTAATCAATACAGAAAATGCCAATCCAAC
TATGGTAGGGTAGGATGGCCACGACTTAAAAAGTTGATGGAAGTCTCAGAATTTCGAAACCAAAATCATTAAAAAGGATTAAGGATAAGAAGTATCCATCTTCATCAA
TCATTATCACATTAACAACAACAAAACATCTTAATAACCTAGCCCAGCCCTGAAGCCAGCTTAATTTATCATCGTATTTTACAAAGATTGCTGCTGCTGCTGCTGGCG
CTCCAAGCATATGAATATTTTTACTTGTTTTTATTTGGAAACTATTATGGATATACTTTAAAACGACTGGAAAAACAACAATTATGGATATACTTTAAAACGACTGGA
AAAACAACAACATAGTTCGAGCTACAACACCACCAAAAGCACAAATCTTCGCGGTTGTGTCCCATCTACAAACCCCCATCGTATTGTCCGCCCAATCCCAACCTTTGT
CCAAACCCTCAGCTTTGTACCCAATCAAAAAACAATAAAATAGCAAAAATAACAAAACAACGGGAAGGTAACGAACAGTTTAATTCCTGTTAGCTTGTTAATTGTCTT
TCATGTCAGCTACCCAAAACCACGTCGTCTTGCTTTCTTTCTGAGCATTTTCGACTTTTTATTATAAAATATTAATAAAATTACACAACCTAAACAAATCACAGTTGG
GAACCTTGCCAGTTGGTTGCCAATCGACAGAAATAAAGACCAACAATAGTACCACTATGCTCCCTCTCTGCGCCTTGTTCACTATTTTCCCTCTTCTTTCTTTTTCAT
AGATCTTGCCTTAATACAAC**ATG**

>M.F12/1|denovo *HXK3* promoter

GGTGGGTGGCAGCAGTTGGTCACTTCTCTTCTATTTTATTTGGTTTCGTCTCGTGGACTGGGGCAAAAGAGAGGGACTCTTCGCCAATATCCAACACCAACCAATTCC
AATTACAATTTTTCATAATGTTGGGTCTTCTTCTGCACTCTTGCTTTTGGTCAAATCCAACCCAGACTCAAATCCAGCCCACAAACTGGCCTCAAACTTGGGCTGGAC
CGAAACTCACAAAATCGGGCTATAATGCACATGACCCGTGGGCCTACCAACTACACATCTATCCATGTAGAGAGAGAGAGAGAGAGAGAGAGAGAGGGGTAGAATAGG
ATGGCCACAACTTAAAAAGTTGATGAAAGTCTCAGAATTTCGAAACCAGAATCATTAAAAGGGATTAAGGATAAGAATTATCCACCTTCATCAATCATTATCACATTA
ACAACAAAATAACATCTTAACAACCTAGCCCAGCCCTGAACCCAGCTTAATTTACCATCGTATTTTAAAACGAATGGAAATACAACAACATGTTGCAATCAATTCGAG
CTATAACAACACCAAAAGCACAGATCTTCGCTCTTGTGTCCCATCTACAAACCCGCATCGGATTGTCCGCCCAATCCCAACCTTTGTCCAAACACTCAGCTTTGTACG
CAATCAAAAAACAATAAAAAAGCAAAAATACCAAAACAACGGGAAGGTAACGATGACTAATGAACGGGCGCATAAAAAAATGATGAGTTTAATTCCAGTTAGCTTGTT
AACTGTCTGTCATTTCAGCTACCCAAAACCACGTCGTCTTGCTTTCTTTCTTAGCATTTTCGACTTTTTATTATAAAATTACAGAACCTAAACAAAAAACCACAGTTG
GGAACCTTGCCAGTTGGTTGCCATCGACAGAAATAAAGACCAACAATAGTACCGCTATGCTCCCTCTCTGTGTCTTGTTCACTATTTTCCCTCTTCTTTCTTTTTCAT
AGATCTTGCCTTACTACAAC**ATG**

>M.2624|denovo *HKX3* promoter

CTTATGGTCAAATCTAACCCAGACTCAAATCCAGCCCACAAACTTGGGCTGGGCCGAAACTCACAAAATCAGACTCGTGGGCCTTCCAACTACACATCCATCCGTAGG
GAAAGAGAGAGGGAGAGAGCAACCACAGCACATGCGATGGGACAGAAAGAGCAATGGTATGTATGGCATGCCGTTTCTATCAAGTTCCCAAAGTAATCAATACAGAAA
ATGCCAATCCAACTATGGTAGGATAGGATGGCCACAACTTAAAAAGTTGATGAAAGTCTCAGAATTTAAAACCAGAATCATTAAAAAGGATTAAGGATAAGAATTATC
CATCTTCATCATCAATCATTACCACATTAACAACAAAATAACATCTTAATGACCTAGCCCAGCCCTATAGATGCGATGATTGTATAAAAAGGAATTGCCGCTGCTGCT
GGCGCTCCAAGCATATGAATATTATTACTTGTTTTTATTTGGTAACTATTATGGATATACTTTAAAACGAATAGAAAAACAACAACGACATGTTGCAATCAGTTCGAG
CTATAACAACACCAAAAGCAAAACCACAGATCTTCTCTCTTGTGTCCCATCGTATTGTCCGCCCAATCCAACCTTTGTCCAAACCCTCAGCTTTGTACCCAGTCGAAA
AACAATAAAATACCAAAACAACTGGCACTGGTAACAATGACTAATCAACGGGCGCATCAAAAACCGATGACTAACGAACAGTTTAATTCCAGTTAGCTTGTTAACTGT
CTGTCGTGTCAGCTACCCAAAACCACGTCGTCTTGGTTTCTTTCTGAGCATTTTCGACTTTTTATTATAAAATATTAATAAAATTGCAGAACCTAAACAAATCATAGT
TGGGAACCTTGCCAATTGGTTGCCATCCACAGAAATAAAGACCAACAATAGTACCACTATGCTCCCTCTCTGTCTTGTTCACTATTTTCCCTCTTCTTTCTTTTTAAT
AGATCTCGCCTTACTACAAA**ATG**

Figure S2. Nucleotide sequences of *Prunus HXK3* promoters. 992 bp upstream regions of the transcription starting sites of the *HXK3* genes.

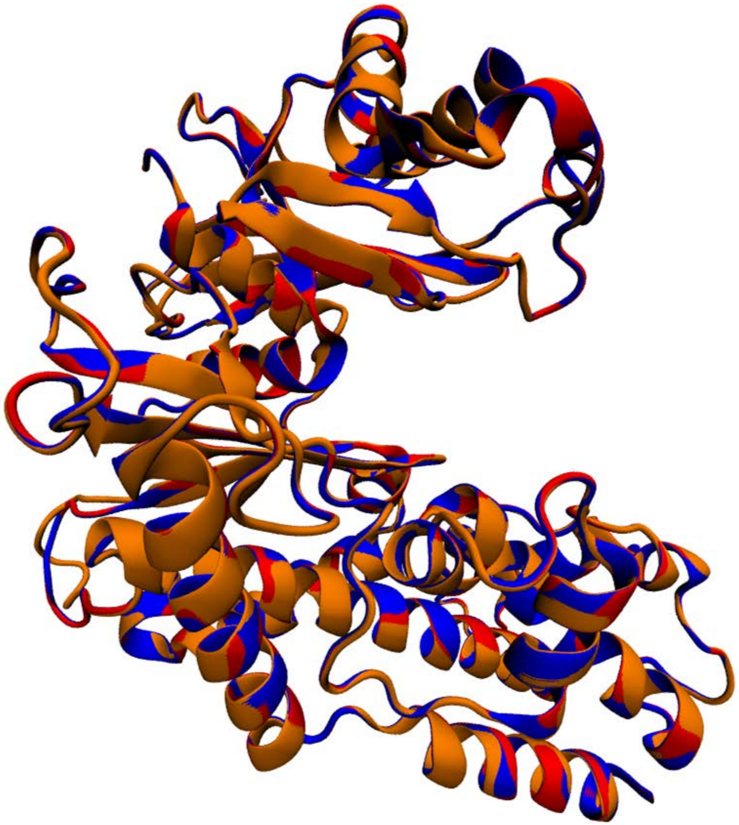

Figure S3. Protein modeling of HXK3 proteins. Comparison through a Richardson diagram of the structural models of the HXK3 proteins of the Mariana 2624 (red), Mazzard F12/1 (orange) and *Prunus persica* (blue).

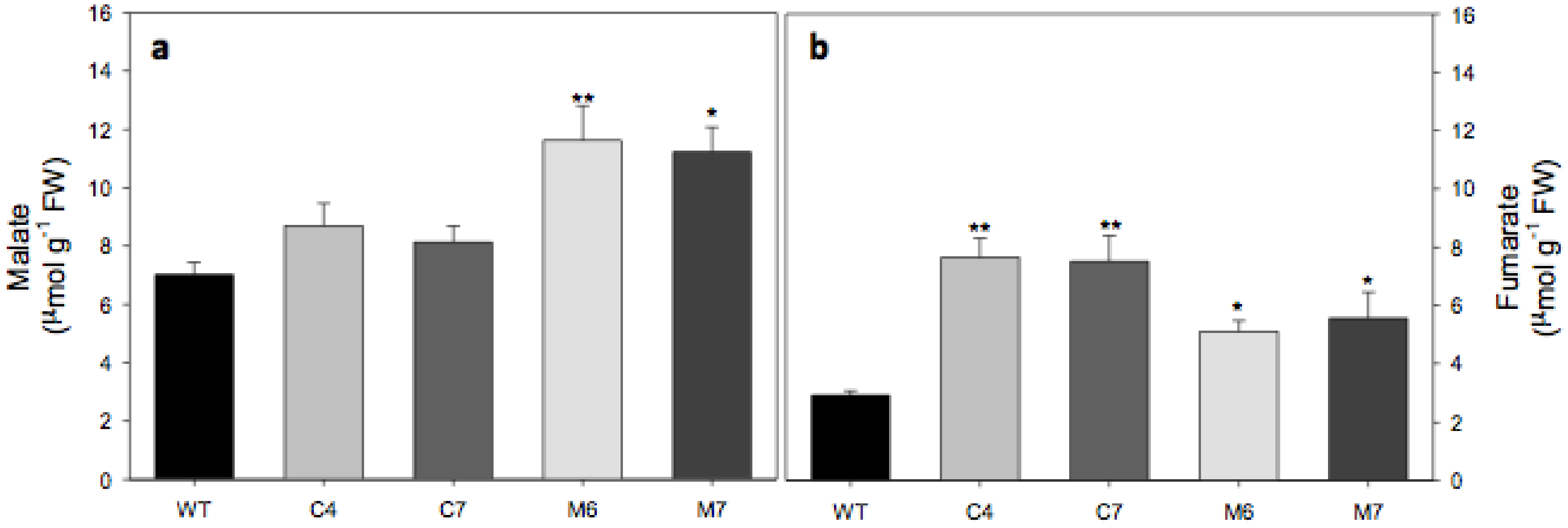

Figure S4. Organic acids content in *HXK3* transgenic lines of *Arabidopsis thaliana* plant growing under optimal condition. a Malate and **b** Fumarate content in *A. thaliana* leaves. Values are presented as means ± SE (n = 5). Asterisks indicate that the values from lines were determined by Student's t test to be significantly different (** (P<0.01) and * (P<0.05)) from the wild-type .

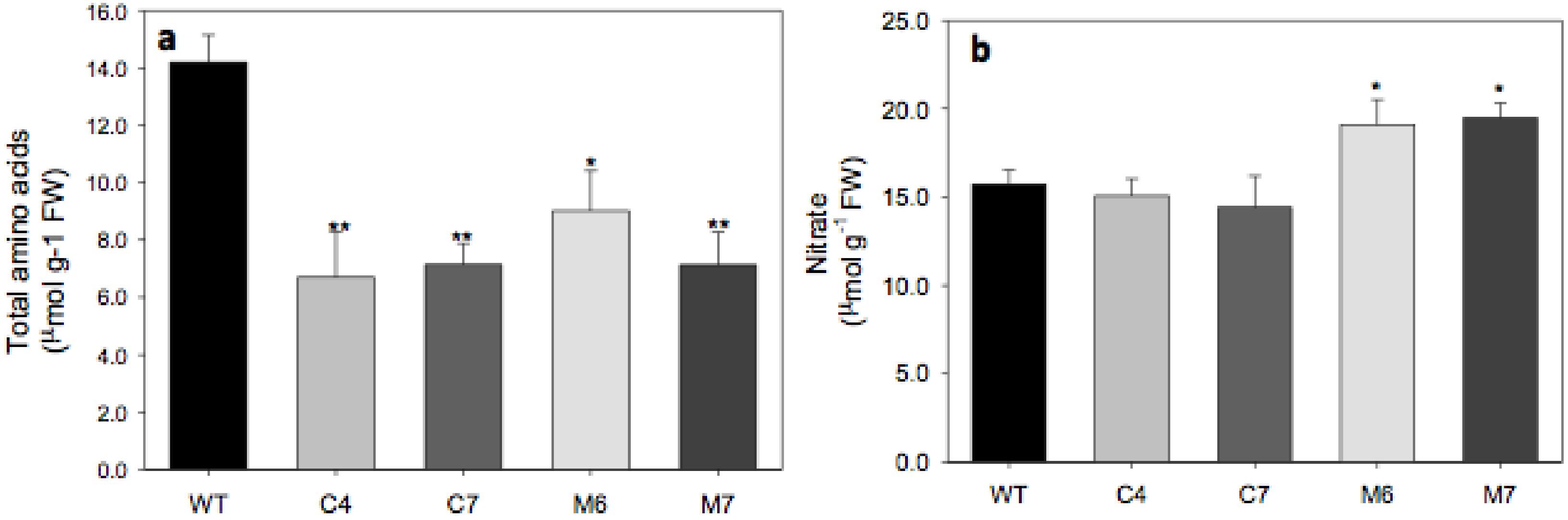

Figure S5. Nitrogen compounds in *HXK3* transgenic lines of *Arabidopsis thaliana* plant growing under optimal condition. a Total amino acids and **b** nitrate content in *A. thaliana* leaves. Values are presented as means ± SE (n = 5). Asterisks indicate that the values from lines were determined by Student's t test to be significantly different (** ($P<0.01$) and * ($P<0.05$)) from the wild-type.

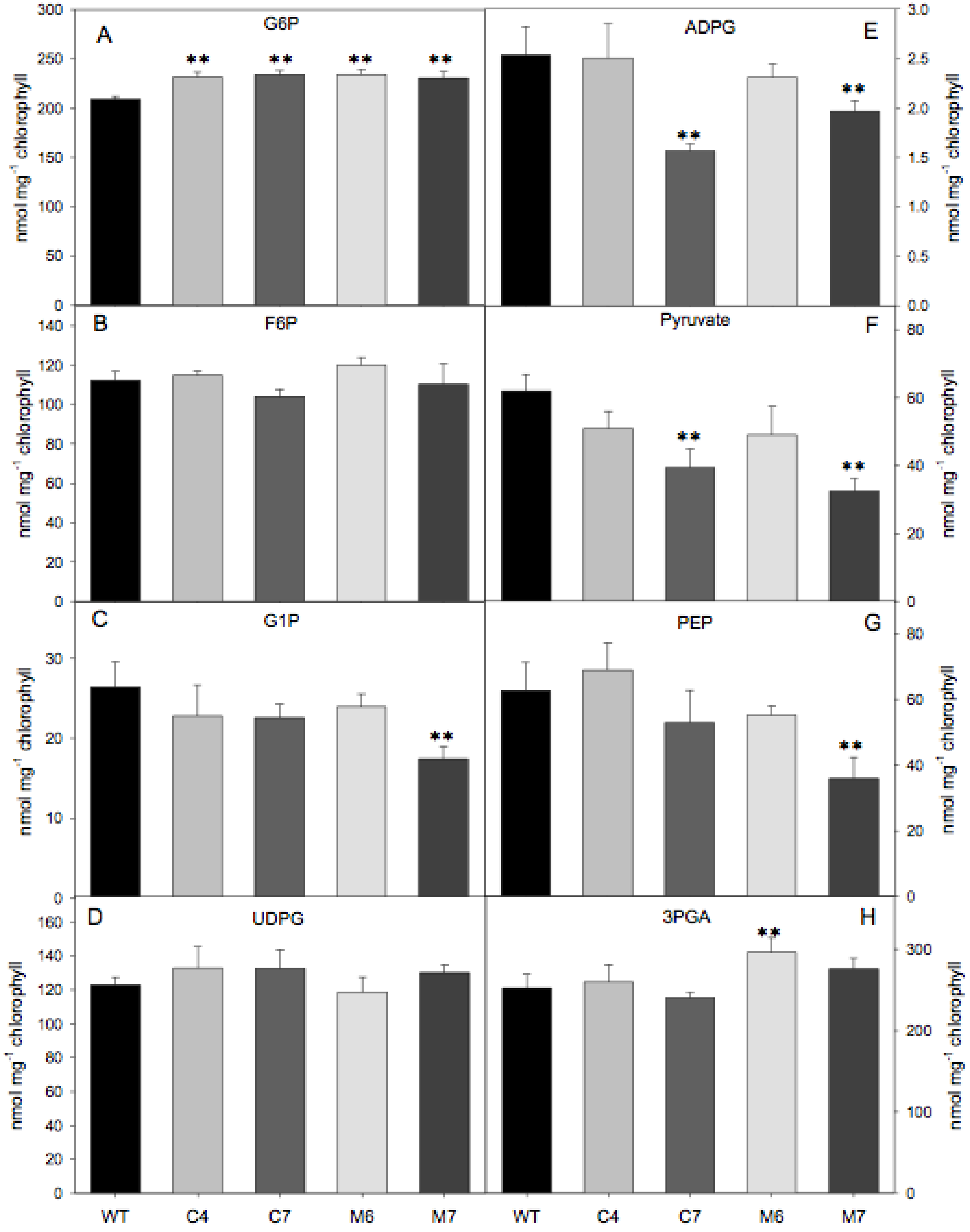

Figure S6. Phosphorylated metabolites and glycolytic intermediates content in *HXK3* transgenic lines of *Arabidopsis thaliana* plant growing under optimal condition. a Glucose-6-phosphate, **b** Fructose-6-phosphate, **c** Glucose-1-phosphate, **d** UDP-glucose, **e** ADP-glucose, **f** Pyruvate, **g** Phosphoenolpyruvate and **h** 3-phosphoglycerate content in *A. thaliana* leaves. Values are presented as means ± SE (n = 5). Asterisks indicate that the values from lines were determined by Student's t test to be significantly different (** (P<0.01) and * (P<0.05)) from the wild-type
