# Supplementary material for: Prunus Hexokinase 3 genes alter primary C-metabolism and promote drought and salt stress tolerance in Arabidopsis transgenic plants
Source: Sci Rep. 2021 Mar 29;11:7098. doi: 10.1038/s41598-021-86535-1 (PMC8007757; doi:10.1038/s41598-021-86535-1)
Supplement: Supplementary file 2 — Supplementary Information 2. [file 41598_2021_86535_MOESM2_ESM.pdf]

Title:
Prunus Hexokinase 3 genes alter primary C-metabolism and promote drought and salt stress tolerance in *Arabidopsis* transgenic plants

Jorge Pérez-Díaz[1], Willian Batista-Silva[2], Rubén Almada[3], David B. Medeiros[2], Stéphanie Arrivault[4], Francisco Correa[1], Adriana Bastías[5], Pamela Rojas[1], María Francisca Beltrán[1], María Francisca Pozo[1], Wagner L. Araújo[2] and Boris Sagredo[1].

[1]Instituto de Investigaciones Agropecuarias CRI Rayentué, Av. Salamanca s/n, Sector Los Choapinos, Rengo, Chile
[2]Max Planck Partner Group at the Departamento de Biologia Vegetal, Universidade Federal de Viçosa, 36570-900, Viçosa-MG, Brazil
[3]Centro de Estudios Avanzados en Fruticultura, CEAF, Camino Las Parcelas 882, Sector Los Choapinos, Rengo, Chile
[4]Max Planck Institute of Molecular Plant Physiology, Am Mühlenberg 1, D-14476 Potsdam-Golm, Germany
[5]Universidad Autónoma de Chile. Av. Pedro de Valdivia 425, Providencia, Santiago, Chile

Name	Len	cTP	mTP	SP	other	Loc
AtHXK3 (*Arabidopsis thaliana*)	493	**0.992**	0.0006	0.0	0.0016	C
HXK3 (*P. persica*)	494	**0.973**	0.0015	0.0	0.0238	C
HXK3 M.F12/1 (*P. avium*)	494	**0.974**	0.0065	0.0	0.0181	C
HXK3 M.2624 (*P. cerasifera x P. munsoniana*)	494	**0.972**	0.0017	0.0	0.0238	C

Table S1. Subcellular localization prediction of the HXKs proteins in *A. thaliana*, *P. persica*, and *Prunus* rootstock genotypes. The highest TargetP scores are written in bold font. cTP: chloroplast transit peptide score; mTP: mitochondrial targeting peptide score; SP: Signal peptide.

Metabolites	WT	C4	C7	M6	M7
Alanine	1.00 ± 0.14	0.76 ± 0.37	0.67 ± 0.21	1.14 ± 0.46	**1.83 ± 0.11**
Asparagine	1.00 ± 0.35	**1.99 ± 0.17**	0.98 ± 0.43	0.97 ± 0.71	0.80 ± 0.38
Aspartate	1.00 ± 0.18	0.80 ± 0.19	0.93 ± 0.14	1.01 ± 0.28	1.13 ± 0.24
Glutamate	1.00 ± 0.15	0.86 ± 0.37	0.91 ± 0.24	1.12 ± 0.42	0.88 ± 0.24
Glutamine	1.00 ± 0.13	1.46 ± 0.61	**0.56 ± 0.09**	1.34 ± 0.16	0.91 ± 0.29
Glycine	1.00 ± 0.23	0.96 ± 0.99	0.60 ± 0.25	0.94 ± 0.82	0.90 ± 0.46
Isoleucinie	1.00 ± 0.12	1.22 ± 0.12	**1.56 ± 0.07**	1.15 ± 0.11	1.24 ± 0.22
Methionine	1.00 ± 0.13	0.91 ± 0.13	0.75 ± 0.18	1.10 ± 0.50	0.87 ± 0.17
Ornithine	1.00 ± 0.15	1.21 ± 0.28	1.09 ± 0.12	1.09 ± 0.28	**0.59 ± 0.02**
Proline	1.00 ± 0.14	0.80 ± 0.26	0.69 ± 0.07	0.97 ± 0.72	0.74 ± 0.27
Pyroglutamate	1.00 ± 0.24	0.96 ± 0.93	0.68 ± 0.35	0.88 ± 0.49	0.97 ± 0.27
Serine	1.00 ± 0.23	0.77 ± 0.22	0.83 ± 0.25	1.07 ± 0.22	0.78 ± 0.29
Threonine	1.00 ± 0.19	0.80 ± 0.10	0.89 ± 0.14	1.20 ± 0.34	0.88 ± 0.28
Valine	1.00 ± 0.17	0.85 ± 0.20	0.87 ± 0.21	1.27 ± 0.24	0.80 ± 0.23
Ascorbate	1.00 ± 0.10	0.98 ± 0.20	1.10 ± 0.34	**1.46 ± 0.18**	0.80 ± 0.34
Citrate	1.00 ± 0.23	**2.48 ± 0.20**	**2.40 ± 0.28**	**2.74 ± 0.23**	**2.28 ± 0.38**
Dehydroascorbate	1.00 ± 0.32	0.77 ± 0.20	0.95 ± 0.29	1.16 ± 0.25	0.66 ± 0.23
Fumarate	1.00 ± 0.11	**1.82 ± 0.18**	**1.76 ± 0.02**	1.35 ± 0.24	1.18 ± 0.21
Glycerate	1.00 ± 0.14	0.64 ± 0.26	0.70 ± 0.08	0.92 ± 0.34	0.76 ± 0.27
Isocitrate	1.00 ± 0.23	1.03 ± 0.80	0.69 ± 0.24	1.40 ± 0.57	0.73 ± 0.12
Lactate	1.00 ± 0.17	0.81 ± 0.04	0.74 ± 0.12	1.37 ± 0.22	1.33 ± 0.46
Malate	1.00 ± 0.09	1.44 ± 0.51	1.36 ± 0.50	**1.74 ± 0.15**	**1.62 ± 0.15**
Succinate	1.00 ± 0.06	1.17 ± 0.23	1.19 ± 0.44	**1.94 ± 0.15**	1.17 ± 0.27
Threonate	1.00 ± 0.10	0.95 ± 0.11	1.15 ± 0.32	1.48 ± 0.55	1.02 ± 0.30
Allose	1.00 ± 0.07	1.06 ± 0.17	1.07 ± 0.25	1.25 ± 0.20	**1.35 ± 0.18**
Fructose	1.00 ± 0.31	1.85 ± 0.38	1.69 ± 0.23	0.65 ± 0.51	**0.40 ± 0.14**
Fructose-6-phosphate	1.00 ± 0.14	1.10 ± 0.14	1.31 ± 0.28	**1.61 ± 0.23**	**1.59 ± 0.10**
Galactinol	1.00 ± 0.09	3.32 ± 1.48	2.66 ± 1.23	5.23 ± 4.95	1.35 ± 1.16
Galactose	1.00 ± 0.21	**0.30 ± 0.39**	**0.39 ± 0.04**	0.73 ± 0.15	**0.27 ± 0.11**
Glucose	1.00 ± 0.39	0.30 ± 0.04	0.39 ± 0.15	**0.43 ± 0.12**	**0.27 ± 0.16**
Lyxose	1.00 ± 0.16	0.78 ± 0.20	0.85 ± 0.22	1.07 ± 0.40	0.53 ± 0.08
Mannose	1.00 ± 0.07	0.96 ± 0.08	1.06 ± 0.25	1.24 ± 0.20	3.45 ± 4.15
Psicose	1.00 ± 0.31	0.85 ± 0.38	0.69 ± 0.23	1.32 ± 0.51	0.40 ± 0.34
Ribose	1.00 ± 0.06	0.81 ± 0.12	0.80 ± 0.10	1.36 ± 0.25	1.03 ± 0.09
Ribulose-5-phosphate	1.00 ± 0.24	0.82 ± 0.17	0.87 ± 0.21	1.25 ± 0.39	0.86 ± 0.14
Sorbitol	1.00 ± 0.25	1.02 ± 0.45	0.89 ± 0.41	1.39 ± 0.35	0.42 ± 0.31
Sucrose	1.00 ± 0.16	0.98 ± 0.28	0.85 ± 0.21	0.73 ± 0.29	0.76 ± 0.34
Talose	1.00 ± 0.24	1.01 ± 0.43	0.88 ± 0.39	1.39 ± 0.22	0.43 ± 0.31
Xylulose	1.00 ± 0.11	0.77 ± 0.18	0.82 ± 0.21	1.16 ± 0.31	1.04 ± 0.21
Glycerol	1.00 ± 0.13	0.64 ± 0.03	**0.51 ± 0.17**	1.03 ± 0.23	0.67 ± 0.17
Guanidine	1.00 ± 0.17	1.02 ± 0.57	0.74 ± 0.25	1.66 ± 0.38	0.77 ± 0.23
Putrescine	1.00 ± 0.17	**0.59 ± 0.10**	**0.43 ± 0.12**	**0.65 ± 0.20**	**0.61 ± 0.14**

Table S2. Relative metabolite content in leaves (4-week-old plants) of WT and *HXK3* transgenic lines. Data are normalized with respect to the mean relative amount calculated for WT (to allow statistical assessment, individual plants from this set were normalized in the same way). Data are presented as means ± SE (n = 5). Values set in bold in *HXK3* transgenic lines plants were determined by the Student's t test to be significantly different (P < 0.05) from WT.
