# Supplementary material for: Prunus Hexokinase 3 genes alter primary C-metabolism and promote drought and salt stress tolerance in Arabidopsis transgenic plants
Source: Sci Rep. 2021 Mar 29;11:7098. doi: 10.1038/s41598-021-86535-1 (PMC8007757; doi:10.1038/s41598-021-86535-1)
Supplement: Supplementary file 3 — Supplementary Information 3. [file 41598_2021_86535_MOESM3_ESM.pdf]

Title:

Prunus Hexokinase 3 genes alter primary C-metabolism and promote drought and salt stress tolerance in Arabidopsis transgenic plants

Jorge Pérez-Díaz[1], Willian Batista-Silva[2], Rubén Almada[3], David B. Medeiros[2], Stéphanie Arrivault[4], Francisco Correa[1], Adriana Bastías[5], Pamela Rojas[1], María Francisca Beltrán[1], María Francisca Pozo[1], Wagner L. Araújo[2] and Boris Sagredo[1].

[1]Instituto de Investigaciones Agropecuarias CRI Rayentué, Av. Salamanca s/n, Sector Los Choapinos, Rengo, Chile

[2]Max Planck Partner Group at the Departamento de Biologia Vegetal, Universidade Federal de Viçosa, 36570-900, Viçosa-MG, Brazil

[3]Centro de Estudios Avanzados en Fruticultura, CEAF, Camino Las Parcelas 882, Sector Los Choapinos, Rengo, Chile

[4]Max Planck Institute of Molecular Plant Physiology, Am Mühlenberg 1, D-14476 Potsdam-Golm, Germany

[5]Universidad Autónoma de Chile. Av. Pedro de Valdivia 425, Providencia, Santiago, Chile

>AT2G19860.1
MGKVAVATTVVCSVAVCAAAALIVRRRMKSAGKWARVIEILKAFEEDCATPIAKLRQVAD
AMTVEMHAGLASEGGSKLKMLISYVDNLPSGDETGFFYALDLGGTNFRVMRVLLGGKHDR
VVKREFKEESIPPHLMTGKSHELFDFIVDVLAKFVATEGEDFHLPPGRQRELGFTFSFPV
KQLSLSSGTLINWTKGFSIDDTVDKDVVGELVKAMERVGLDMLVAALVNDTIGTLAGGRY
TNPDVVVAVILGTGTNAAYVERAHAIPKWHGLLPKSGEMVINMEWGNFRSSHLPLTEYDH
SLDVDSLNPGEQILEKIISGMYLGEILRRVLLKMAEEAAFFGDIVPPKLKIPFIIRTPNM
SAMHSDTSPDLKVVGSKLKDILEVQTSSLKMRKVVISLCNIIASRGARLSAAGIYGILKK
IGRDATKDGEAQKSVIAMDGGLFEHYTQFSESMKSSLKELLGDEVSESVEVILSNDGSGV
GAALLAASHSQYLELEDDSETS*
>AT2G19860.2
MRVLLGGKHDRVVKREFKEESIPPHLMTGKSHELFDFIVDVLAKFVATEGEDFHLPPGRQ
RELGFTFSFPVKQLSLSSGTLINWTKGFSIDDTVDKDVVGELVKAMERVGLDMLVAALVN
DTIGTLAGGRYTNPDVVVAVILGTGTNAAYVERAHAIPKWHGLLPKSGEMVINMEWGNFR
SSHLPLTEYDHSLDVDSLNPGEQILEKIISGMYLGEILRRVLLKMAEEAAFFGDIVPPKL
KIPFIIRTPNMSAMHSDTSPDLKVVGSKLKDILEVQTSSLKMRKVVISLCNIIASRGARL
SAAGIYGILKKIGRDATKDGEAQKSVIAMDGGLFEHYTQFSESMKSSLKELLGDEVSESV
EVILSNDGSGVGAALLAASHSQYLELEDDSETS*
>AT4G29130.1
MGKVAVGATVVCTAAVCAVAVLVVRRRMQSSGKWGRVLAILKAFEEDCATPISKLRQVAD
AMTVEMHAGLASDGGSKLKMLISYVDNLPSGDEKGLFYALDLGGTNFRVMRVLLGGKQER
VVKQEFEEVSIPPHLMTGGSDELFNFIAEALAKFVATECEDFHLPEGRQRELGFTFSFPV
KQTSLSSGSLIKWTKGFSIEEAVGQDVVGALNKALERVGLDMRIAALVNDTVGTLAGGRY
YNPDVVAAVILGTGTNAAYVERATAIPKWHGLLPKSGEMVINMEWGNFRSSHLPLTEFDH
TLDFESLNPGEQILEKIISGMYLGEILRRVLLKMAEDAAFFGDTVPSKLRIPFIIRTPHM
SAMHNDTSPDLKIVGSKIKDILEVPTTSLKMRKVVISLCNIIATRGARLSAAGIYGILKK
LGRDTTKDEEVQKSVIAMDGGLFEHYTQFSECMESSLKELLGDEASGSVEVTHSNDGSGI
GAALLAASHSLYLEDS*
>AT4G37840.1
MTRKEVVLAVTAATITAVAAGVLMGRWIRRKERRLKHTQRILRKFARECATPVSKLWAVA
DALVADMTASLTAECCGSLNMLVSFTGSLPSGDEKGVHYGVNLRGKELLLLRGTLGGNEE
PISDVQKHEIPIPDDVLNGSFKELCDFISLELVKFLAMNPGGEAEEVKNLGFTLTRSVEQ
IGSHSISSIHRKSLANDDDEKVLKDLVNDMNESLETHGLKIRMNTALVDNTIGELAGGRY
YHKDTVAAVSLGMGTNAAYIEQAQEISRWKSAIREPQEIVVSTEWGDFRSCHLPITEFDA
SLDAESLNPGHRIFEKMVSGRYLGEIVRRVLLKMSEESALFGDTLPPKLTIPYILWSPDM
AAMHQDISEERETVNKKLKEVFGIMDSTLAAREVVVEVCDVVAERAARLAGAGIVGMIKK
LGRLEKKMSIVIVEGGLYDHYRVFRNYLHSSVWEMLGDELSDHVVIEHSHGGSAAGALFL
AACGDGHQDSESK*
>AT1G50460.1
MGKVAVAFAAVAVVAACSVAAVMVGRRMKSRRKWRTVVEILKELEDDCDTPVGRLRQVVD
AMAVEMHAGLASEGGSKLKMLLTFVDDLPTGREKGTYYALHLGGTYFRILRVLLGDQRSY
LDVQDVERHPIPSHLMNSTSEVLFNFLAFSLERFIEKEENGSDSQGVRRELAFTFSFPVK
HTSISSGVLIKWTKGFEISEMVGQDIAECLQGALNRRGLDMHVAALVNDTVGALSLGYYH
DPDTVVAVVFGTGSNACYLERTDAIIKCQGLLTTSGSMVVNMEWGNFWSSHLPRTSYDID
LDAESSNANDMGFEKMISGMYLGDIVRRVILRMSEDSDIFGPISPVLSEPYVLRTNSVSA
IHEDDTPELQEVARILKDIGVSDVPLKVRKLVVKICDVVTRRAGRLAAAGIAGILKKIGR
DGSGGITSGRSRSEIQMQKRTVVAVEGGLYMNYTMFREYMEEALVEILGEEVSQYVVVKA
MEDGSSIGSALLVASLQS*
>AT1G47840.1
MSLMFSSPVVTPALGSFTFSSRPRSNYIVMSAVRSNSASTCPILTKFQKDCATPTPYLRN
VANAIADDMRDGLAVEGGGDLEMILTFVDALPSGNEEGLFYALDLGGTNFRVRSVQLGGK
KERVLATESEQISISQKLMIGTSEELFGFIASKLANFVAKEKPGRFLLEEGRKRELGFTF
SFPVKQTSIDSGTLSKWTKGFKVSGMEGKNVVACLNEAMEAHGLDMRVSALVNDGVGTLA
GARYWDEDVMVGVILGTGTNACYVEQKHAIPKLRSKSSSGTTIINTEWGGFSKILPQTIF

DLEMDETSLNPGEHLYEKMISGMYLGEIVRRVLLHMCETSDLFGHFAPAKLSTPLALRTE
HLCKMQEDNTDDLRDVGSILYDFLDVEANMNARRRVVEVCDTVVKRGGRLAGAGIVAILE
KIEKDTKRMGSGKRTVVAMDGALYEKYPQYRQYMQDALVELLGHKLASHVAIKHTKDVSG
LGAALLAATNSIY*
>AT3G20040.1
MGKVLVMLTAAAAVVACSVATVMVRRRMKGRRKWRRVVGLLKDLEEACETPLGRLRQMVD
AIAVEMQAGLVSEGGSKLKMLLTFVDDLPNGSETGTYYALHLGGSYFRIIKVHLGGQRSS
LEVQDVERHSIPTSLMNSTSEVLFDFLASSLQRFIEKEGNDFSLSQPLKRELAFTFSFPV
KQTSISSGVLIKWTKGFAISEMAGEDIAECLQGALNKRGLDIRVAALVNDTVGALSFGHF
HDPDTIAAVVFGTGSNACYLERTDAIIKCQNPRTTSGSMVVNMEWGNFWSSRLPRTSYDL
ELDAESMNSNDMGFEKMIGGMYLGDIVRRVILRMSQESDIFGPISSILSTPFVLRTNSVS
AMHEDDTSELQEVARILKDLGVSEVPMKVRKLVVKICDVVTRRAARLAAAGIAGILKKVG
RDGSGGGRRSDKQIMRRTVVAVEGGLYLNYRMFREYMDEALRDILGEDVAQHVVVKAMED
GSSIGSALLLASSQSVQTIPSV*
>Fve|XP_004287480.1
MGKVAVGAAVVCAAVTCAAAALLVRRRLKSSRRWARAMAILKEFEEKCDTPIGKLRQVAD
AMTVEMHAGLASEGGSKLKMLISYVDNLPTGDEKGLFYALDLGGTNFRVLRVQLGGKEKR
VIKQEFDEVSIPPHLMTGTSEGLFDFIAEALAKFVATEGEGFHPAPGRLRELGFTFSFPV
WQTSIASGTLIKWTKGFSIEDAVEQDVVAELTKSVEKIGLDMRVTALVNDTIGTLAGGRY
HNPHVIAAVILGTGTNAAYVERAHAIPKWHGLLPKSGEMVINMEWGNFRSSHLPHTEYDQ
ALDAESLNPGEQIFEKIISGMYLGDIVRRVLLKMAEEAAFFGDTVPHKLKIPFVLRTPDM
SAMHHDSSPDLRVVGSKLKDILEISNTSLKVRKAVVALCDIVATRGARLSAAGILGVLKK
LGRDTVKDGEKHKSVVALDGGLFEHYTEFRVCMESTLDELLGDDVAEHISVEHSNDGSGI
GAALLAASHSHYLGVEDS
>Fve|XP_004289383.1
MRKEVVAAAALTAAAAITAIAALVRHRKRRKEQQWKETQKILRKFARDCATPVPKLWQVA
NAFVADMRSSLIASNGTNTSLNMLVSYVASLPSGDEEGMYYGVNLRRTDFLLLCARLRGK
NDPISDFHREEIHIPTNLLDGTGSTKELFGFIAVELGKFVEAHPNSEMKDRPAKNNKLGF
IISCPVDQAVATSGTAITWKSFAADSTLGKKLVHDFNRALEEHGVKLSVYAMVDDTVGTL
AGGRYYNRESVAAITLGMGTDAAYVEPADAALQWHGPSPKLGEMVISTQWGEFSTPHLPI
TIFDTCLDAESSNPGFRRFEKVVSGMYLGEVVRRVLLKMAKETALFGQCVVPSKLMTPYQ
LSSPDMAAMHQDTSDNREVVGEKLKEVFGITKSTPEVREVVAEVCDIVAERGARLAGAGI
LGIIKKLGRIENKRSIVTVEGGLYEHYRVFRNYLNSGVWEMLGDNHSDNVVIENSHGGSG
TGALYLAASQMKDIDQSQMQDSDPDVEAKPEPEPEPEF
>Fve|XP_004291365.1
MSVTAATSPSVGSFHLCRRSTRPAGLPRFRMTVRSTAVSVTPLVTKLRKECATPLPLLRH
VADAMAADMRAGLAGDGGSDLKMILSYVDSLPTGNEKGLFYALDLGGTNFRVLRVQLGGK
EERVIATEFDQVSIPKDLMFGTSEELFDFIASGLAKFAENEGNKFHLPAGTKREIGFTFS
FPVKQTSVDSGILIKWTKGFSVSGTAGRDVVACLNEAMERLGLDMRVSALVNDTVGTLAG
ARYWDDDVMVAVILGTGTNACYVERVDAVPKLQNHASSSGSTIINTEWGAFSNGLPLTEF
DRDMDAASINPGEQIFEKTISGMYLGEIVRRVLLKMAEADALFGDPILEKLSTPFALRTP
DVCAMQQDTSDDLQDVGSILYNVAGVESNLSARKIVVEVCDTIVKRGGRLAGAGIVGILQ
KMEEDSKGVIFGKRTVVAMDGGLYENYPQYRTYLSEAVTELLGDDIAKNVVIEHSKDGSG
IGAALLAAANSKYL
>Fve|XP_004302852.1
MGKKAVMIVCGAAVVTVAAAVLVRQRMRCCGEWSKATAIVKELEEKCGTPIAKLRQVADA
MTVEMHAGLASEGGSKLKMIISYVDNLPTGNEKGLFYALDLGGTNFRVLRVQLGGKGRGL
VNQEFTEVSIPEGLMVGTTETLFDFIATELAKFVGKEGPEYQLPPGRQRELGFTFSFPVQ
QTSINSGTLIKWTKGFSIDDTVGRDVVAELSKAIEKQGLDMRVSALVNDTVGTLAGGIYN
NNDVVAAVILGTGTNAAYVERAQAIPKWHGLLPKSGDMVINMEWGNFRSAHLPLTDYDHS
LDTESLNPGDQIFEKIISGMYLGEIVRRALYRIAEEASIFGDIVPPKLKVPFILRTPDMS
AMHHDTSYDLKVVRDILKNVLEISNTSLKVRKLIVELCNTVATRGARLAAAGILGVLKKL
GRDVFRDGEKQKKTVIALDGGLFEHYTEYSKCMENTLRELLGEEVGASIVIEHSNDGSGI
GAALIAASHSQYLQDDES

>Mdo|NP_001280783.1
MGKKAVIIVTATAVCAAAAVLVVRHRMRSSGRWARASAIIKELEEKCGTPTEKLRQVADA
MAVEMHAGLASEGGSKLKVIISYVDNLPTGNEKGLFYALDLGGTNFRVLRVRLGGKGRGI
VSQEFIEVSIPENLMVGTSDALFDYIAAELAKFVAKEGQDHQPPPGRQRELGFTFSFPVL
QSSINSGTLLKWTKGFSIDDAVGQDVVAALSKAIERTGLDMCVSALVNDTIGTLAGGRYI
NQDVVAAVILGTGTNAAYVERAQAIPKWHGLLPKSGEMVINMEWGNFKSAHLPLTEYDHS
LDTESLNPGDQIFEKIISGMYLGEIVRRVLCRIAEEASLFGDTIPPKLKVPFNLRTPDMS
AMHQDASSDLRVVREKLKNVLEISNTSLKVRKVIVELCNIVATRGARLAAAGVLGVLKKL
GRDAVKDGENKKTVVALDGGLYEHYTEYSKCMENTLRELLGEEVAETVVIEHSNDGSGIG
AALLAASHSQYPGIDES
>Mdo|NP_001315646.1
MGKVAVGAAVVCAAAVCAAAALIVRHRMKSSGRWARAMAILRELEDKCGTPIGKLRQVAD
AMTVEMHAGLASEGGSKLKMLISYVDNLPTGDENGLFYALDLGGTNFRVLRVRLGGNEKR
VVKQEFDEVSIPPHLMTGTSEGLFDFIAEALAKFVATEGEGLHPAPSRQRELGFTFSFPV
WQTSIASGTLIRWTKGFNIEDAVEQDVVGELTKSMEKIGLDMRVTALVNDTIGTLAGGRY
HNQDVIAAVILGTGTNAAYVERAHAIPKWHGLLPKSGEMVINMEWGNFRSSHLPLTEYDQ
ALDAESLNPGEQIFEKIISGMYLGEIVRRVLLKMAEEADFFGDVVPPKLKIPFILRTPEM
SAIHHDSSPDLKVVGSKLKDILEISNTSLKMRKVAVAVCDIVATRAARLSAAGMLGVLKK
LGRDTVKDGEKQKSMIAMDGGLYEHYTEFRTSMECTLKELLGDEVAEHISVEHSNDGSGI
GAALLAASHSQYLEVEES
>Mdo|XP_008342598.2
MRKKVAVVASVIVTTTVVAASVLLRQWKRKKQRQWRETQKILRKFARDCATPVPKLWQVS
NALVSDMKACLASGGTITTLNMLVSYVAALPTGDEEGFYYGVNLRGTNFLILCARLGGKN
NPISDLNREEIPIPSNLMAATAKELFDFIAVELGRFVSEHPNTSAKEMSKLGCIVSCPVE
QSVVSDGTAIKWKSFSADSKVGKKLVSDFNKALEEHGVKLRVYAMVDDTVGNLAGGRYYN
RESVAAVTLAMGTDAAYVEPANAALQWHGPSPKLGDMVISTQWGDFSSPHLPITIFDTCL
DAESPNPGCRRFEKLISGMYLGEIVRRVLLKMAQETALFGDTVPSKLMTRYQLSSPDMAA
MHQDTSEDHEIVGEKLKKVFGITSTSPRAREVVSEVCDIVAERGARLAGAGILGIIKKLG
RIENKKSVVTVEGGIYEHYRVFRNYLNSSVWEMLGNDLSDNVVIEHSHGGSGTGALFLAA
SQMEMQNDDPNPDHDPDTDPGNDDDEPDIDDPHPGNDDHDRGNDVRDPNPDT
>Mdo|XP_008363245.2
MGKVAVGLAVGVAVAACAVATMVVGRRIRSRRKWGRVFGVLKDLELSCETTVGRLRQVVD
AMAVEMHAGLASDGGSKLKMLLTFVDKLPDGSEKGTYYALDLGGTNFRVLRVKLGGTRSS
TLEHDVHQQDIPQNLMTGTSEVLFDFIASSVKEFVERETDDSKLSLDRRRELGFMFSFPV
KQTSVSSGTLLKWTKGFAIEDMIGKEVAGCLEKAMTKKGLNMSVAALVNDSVGTLSLGHY
HDTDTVAAVIIGTGTNACYLERTDAIIKSQGLLTASGGMVVNMEWGNFWSSHLPRTVYDT
ELDADSPNPNDQGFEKLISGMYLGDVVRRVIVRMSQESEIFEPISSKLYMPFILRTPLIA
AMHEDDSPDLEEVGRILRENLEIPDVPLKVRKLIVKVCDVVTRRAARLAAAGIVGILKKI
GRDGSGGITGGRSRSDHRMRRTVVAMEGGLYTEYTMFREYLHEALTEILGEDIAKHVVLI
KVTEDGSATGAALLAASHSSYPVDNVRLP
>Mdo|XP_008365574.2
MKKEVVVVATMTAAATVVVAAVLLRQWKRKKQRQWRETQKILRKFARDCSTPVPKLWQVS
NALVSDMKACLASSGTITTLNMLVSYVSPLPTGDEKGFYYGVNLRGTNFLILCARLGGKN
NPISDLHKEEIPIPSNLMAATSKELFDFIAVELGKFVSEHPNTLAEELSKLGCIVSCPVE
QSVVSDGTAIKWKSFSADSKVGKKLVSDFNKALEEHGVKLRVYAMVDDTVGNLAGGRYYN
RESVAAVTLAMGTDAAYVEPANAALQWHGPSPKLGDMVISTQWGGFSSPHLPITIFDTCL
DAESPNPGCRRFEKLISGMYLGEIVRRVLLKMAQETAVFGDTVPLKLMTPYQLSSPDMAA
MHQDTSEDHKIVGEKLKKVFGITSTSPKAREVVSEVCDIVAERGARLSGAGILGIIKKLG
RIENKKSVVTVEGGLYDHYRVFRNYLNSSVWEMLGNDLSDNVVIKHSHGGSGTGALFLAA
SQMQMQNDDPDPDPEPGNDDDDEEPDIDDAHPDNDDHDHEHGNDVRDPNLN
>Mdo|XP_008385759.2
MGKVAVGLAVGVAVAACAVATVVVGRRIRCRRKWSRVVGVLKDLEHSCETTVGRLRQVVD
AMAVEMHAGLASDGGSKLKMLLTFVDKLPNGTEKGTYYALDLGGTNFRVLRVKLGGTRSS
TLEHDVYRQAIPQDLMTGTSEDLFDFIASSLKEFVERERDVSKLSLDRRRELGFTFSFPV

KQISVSSGTLLKWTKGFAIEDMVGREIAGCLEKAMTRKGLNMRVAALVNDSVGTLSLGHY
HDTDTVAAVIIGTGTNACYLERIDAIIKSQGLLTASGSMVVNMEWGNFWSSHLPRTVYDI
ELDADSPNPNDQGFEKLISGMYLGDVVRRVIVRMSQESDIFEPISSKLSMPFILRTPLIA
AMHEDDTPDLEEVGSILRDNLEIPDVPLKVRKLIVKVCDVVTRRAARLAAAGIVGILKKI
GRDGSGGITGGRSRSDHRMRRTVVAMEGGLYTGYTMFREYLHEALTEILGEDIAQHVVLK
VTEDGSAMGAALLAAAYSSYPLDSVQLQ
>Mdo|XP_008387327.2
MGKVAVGAAVVCAAAVCAAAALVVRHRMKSSGRWARAMAILKEFEDKCGTPIGKLRQVAD
AMTVEMHAGLASEGGSKLKMLISYVDNLPTGDEKGLFYALDLGGTNFRVLRVQLGGKDKH
VVKQESEEVSIPPHLMTGTSEGLFDFIAETIAKFVATEGEGLHPAPGRQRELGFTFSFPV
RQTSIASGTLIKWTKGFSIEDAVEQDVVGELTKSVEKIGLDLRVTALVNDTIGTLAGGRY
HNQDVIAAVILGTGTNAAYVERAHAIPKWHGLLPKSGEMVINMEWGNFRSSHLPLTEYDQ
ALDAESLNPGEQIFEKIISGMYLGDIVRRVLLKMAEEADFFGDVVPPKLKIPFVLRTPVM
SAMHHDSSPDLKVVGNKLKDILEISNTSLKMRRVVVALCDIVSTRGARLSAAGILGVLKK
LGRDTVKDGEKQKSVVALDGGLYEHYTEFRTSMEGTLKELLGDEVAEHISIELSSDGSGI
GAALLAASHSQYLEVEES
>Mdo|XP_008387435.2
MGKKAVIIVTATAVCAAAAVLVVRHRMRSSGRWARASAIIKELEEKCGTPTGKLRQVADA
MAVEMHAGLASEGGSKLKMIISYVDNLPTGNEKGLFYALDLGGTNFRVLRVQLGGKGRGI
ISQEFTEVSIPENLMVGTSDALFDYIAAELAKFVAKEGQDYQLPPGRQRELGFTFSFPVL
QSSINSGTLIKWTKGFSIDDAVGQDVVAELSKAIERTGLDMRVSALVNDTVGTLAGGRYV
NQDVVVAVILGTGTNAAYVERAHAIPKWHGLLPKSGEMVVNMEWGNFKSAHLPLTEYDHS
LDTESLNRGDQIFEKIISGMYLGEIVRRVLCRIAEEASLFGDTVPPKLKVPFILRTPDMS
AMHHDASSDLRVVREKLKNVLEISNTSLKVRKVIVELCNIVATRGARLAAAGVLGVLKKL
GRDAVKDGENQKTVVALDGGLYEHYTEYSKCMENTLRELLGEEVAETIVIEHSNDGSGIG
AALLAASHSQYLGIDES
>Mdo|XP_008387945.1
MSAATTSPAVRSFYLRRRSPTVAMPRCRMAVRSAAVSLSPLLTKLHNDSATPLPLLRHVA
DSMASDMRAGLARDGGSDLKMILSYVDSLPSGNEEGLFYALDLGGTNFRVLRVQLGGKDE
RVIATEFDQVSIPPELMCGTSEELFDFIASGLAKFAQKEGNKFHLPTGTKREIGFTFSFP
VKQTSIDSGILIKWTKGFSVSGTAGRDVVACLNEAMERKGLDMRVSALVNDTVGTLAGAR
YWDDDVMVAVILGTGTNACYVERVDAIPKLQGQVSSSGRTIINTEWGAFSNGLPLTEFDR
DMDAASINPGEQIFEKTISGMYLGEIVRRVLVKMAEADALFGESVLEKLSTPFLLRTPYL
CAMQQDASDDLQAVGSILYNVAGVESSLSARKIVVDVCDTIVKRGGRLAGAGIVGILQKM
EEDSKDLIFGKRTVVAMDGGLYENYPQYRRYLQEAVAELLGTEISKNVVIEHSKDGSGIG
AALLAAANSQYI
>Nta|XP_016440775.1
MKKATVGAVVVGAAVTVAVGALIVRHRMRKSSKWARARAILKEFEEKCGTPDAKLKQVAD
AMTVEMHAGLASEGGSKLKMLISYVDNLPTGDEEGVFYALDLGGTNFRVLRVQLGGKDGG
IVHQEFTEASIPPNLMVGTSEALFDYIAAELAKFVAEEEEKFHQPPGKQRELGFTFSFPI
MQTSINSGTLIRWTKGFSIDDTVGQDVVAELTKAMQRKGVDMRVSALVNDTVGTLAGGRF
SNKDVSIAVILGTGTNAAYVERAQAIPKWHGPLPKSGEMVINMEWGNFRSSHLPLTEYDH
AMDTDSLNPGEQIFEKICSGMYLGEILRRVLLRMAEEAAIFGDEVPPKLKNQFILRTPEM
SAMHHDTSSDLRVVGDKLKDILEISNTSLKTRRLVVELCNIVATRGARLAAAGVLGIVKK
MGRDTPRESGQEKIVVAMDGGLYEHYTEYRKCLENTLVELLGEEMATSIVFEHANDGSGI
GAALLAASNSLYVEDKS
>Nta|XP_016445349.1
MGKVVVGAAVVCTAAVCAAAVLLMRHKMKNSGKWAKAMDILKEFEEKCETPIGKLRQVAD
AMTVEMHAGLASEGGSKLKMLISYVDNLPTGDETGLFYALDLGGTNFRVMRVQLGGKEKR
IVKQEVKEVSIPKNVMAGSSSDALFDFIATALVKFVATEGDDFHLPPGRQRELGFTFSFP
VKQLSIASGTLIKWTKGFSIDDAVGQDVVRELTKAMERVGLDVRVAALINDTVGTLAGGR
YNNPDVIAAVILGTGTNAAYVERANTIPKWHGLLPKSGDMVINMEWGNFRSSHLPVTEYD
QSLDAESLNPGEQIYEKMISGMYLGEILRRVLCRMAEEASFFDDYVPPKLKTPFILRTQD
MSAMHHDKSADLKVVGDKLKDILEVPNSTWKMRKIIVELCDIITSRGARLSAAGIVGILK

KLGRDTLKDGEKQRSVIAVDGALFEHYTKFRNCMEGTIKELLGDAAENIVVKLSNDGSGV
GAALLAASHSQYLGLKES
>Nta|XP_016456945.1
MGRLGVGIAAGFAVAACIVAAAMVGKRVKRRRKWKKMVKVLEELEESCGTPVGRLKQVVD
AMAVEMHAGLASEGGSKLKMLLTYVDKLPNGREKGTYYALHLGGTNFRVLRVHLGDQRSA
ILGQDIARQPIPQHLMTSTSEDLLDFVASSLKNFIEKEDDGLEQPSPRRRELGFTFSFPV
KQTSVSSGILIKWTKGFAIEDMIGRDVSECLQQVIARKGQDVRVAALINDTVGTLALGHY
NDEDTVAAVVIGTGTNACYLERADAIIKCQGLLTTSGSMVVNMEWGNFWSSHLPRTSYDV
DLDVASPNPNDQGFEKMISGLYLGDIVRRVLLRMSQESDDFGPASSKLAVPFALRTPLMA
AMHEDDSPDLSEVAKILGEVLELPDVPVKVRKLVVKVCDVITRRAARLAAAGIVGILKKI
GRDGSGGIAGGKFRSDRPSRMRRTVVAIEGGLYTSYTLFREYLNEAMKEILGEEISSNVI
LRVMEDGSGIGAALLAAANSASEVDRVQLQ
>Nta|XP_016465016.1
MKKATVGAVVVGAAATVAVAALIVRHRMRKSSKWARARAILKEFEEKCGTPDAKLKQVAD
AMTVEMHAGLASEGGSKLKMLISYVDNLPTGDEEGVFYALDLGGTNFRVLRVQLGGKDGG
IVHQEFAEASIPPNLMVGTSEALFDYIAAELAKFVAEEEERFHQPPGKQRELGFTFSFPI
MQTSINSGTLIRWTKGFSIDDTVGQDVVAELTKAMQRKGIDMRVSALVNDTVGTLAGGRF
SNKDVCIAVILGTGTNAAYVERAQAIPKWHGPLPKSGEMVINMEWGNFRSSHLPLTEYDH
AMDTDSLNPGEQIFEKICSGMYLGEILRRVLLRMAEEAGIFGDEVPPKLKNQFILRTPEM
SAMHHDTSSDLRVVGDKLKDILEISNTSLKTRRLVVELCNIVATRGARLAAAGVLGIVKK
MGRDTPRESGQEKIVVAMDGGLYEHYTEYRKCLENTLVELLGEEMATSIVFEHANDGSGI
GAALLAASNSLYVEDKS
>Nta|XP_016465440.1
MGRLAVGISAGFAVAACIVAAAMVGKRVKRRRKWKKMVKVLEELEESCGTPVGRLKQVVD
AMAVEMHAGLASEGGSKLKMLLTYVDKLPNGREKGTYYALHLGGTNFRVLRVHLGGQRSA
ILGQDIERQPIPQHLMTSTSEDLLDFVASSLKDFIEKEGNGLEQPSPRRRELGFTFSFPV
KQTSASSGILIKWTKGFSIEDMIGRDVSECLQQAIARKGQDVRVAALINDTVGTLALGHY
NDEDTVAAVVIGTGTNACYLERADAIIKCQGLLTTSGGMVVNMEWGNFWSSHLPRTSYDI
DLDVASPNPNDQGFEKMISGLYLGDIVRRVLLRMSQESDDFGPASSKLAVPFALRTPLMA
AMHEDDSPDLSEVARILGEVLELPDVPVKVRKLVVKVCDVITRRAARLAAAGIVGILKKI
GRDGSGGIAGGKFRSDRPSRMRRTVVAIEGGLYTSYTLFREYLNEAMKEILGEEISSNVI
LRVMEDGSGIGAALLAAANSASEVDRVQLH
>Nta|XP_016468018.1
MSVTVSSPAGRSFHISRSPYKKISKPRVIIAAVRSGVSLAVAPILTKLQKDCATPLPVLR
HVADAMAVDMRAGLAVDGGSDLKMILSYIDTLPTGNEKGLFYALDLGGTNFRVLRVQLGG
KEERVIATEFEQVSIPQELMFATSEELFDFIASELGKFSQSEGGKFEMQQGRTREIGFTF
SFPVKQTSVKSGILIKWTKGFAVSGTAGKDVVACLNEAMERQGLGMQVSALVNDTVATLA
GARYWDNDVMVAVILGTGTNACYVERVDAIPKLPQRMSNSPETIVNTEWGAFSNGLPLTE
FDREMDAESINPGEQIFEKTISGMYLGEIVRRVLVKMAKVGGLFGGGYVPEKLVTPFVLR
TPDICAMQQDTSRDLEAVESVLYDIAGVKSDLSARKTVVDICDTIANRGGRLAGAGIVGI
LQKMEEDSKGVIFGKRTVVAMDGGLYEHYPQYREYLQEAVTELLGSEISKNVVIEHSKDG
SGIGAALLAAANSKYEHDY
>Nta|XP_016475909.1
MKKATVGAAVIGAATVCAVAALIVNHRMRKSSKWARAMAILREFEEKCGTPDAKLKQVAD
AMTVEMHAGLASEGGSKLKMLITYVDNLPTGDEAGVFYALDLGGTNFRVLRVQLGGKDGG
IVHQEFAEASIPPNLMVGTSEALFDYIAAELAKFVNEEGEKFQQPPGKQRELGFTFSFPV
MQTSINSGTIMRWTKGFSIDDAVGQDVVGELAKAMKRKGVDMRVSALVNDTVGTLAGGKY
THNDVAVAVILGTGTNAAYVERVQAIPKWHGPVPKSGEMVINMEWGNFRSSHLPLTQYDH
ALDTNSLNPGDQIFEKMTSGMYLGEILRRVLLRVAEEAGIFGDEVPPKLKSPFVLRTPDM
SAMHHDASSDLRVVGDKLKDILEISNTSLKTRRLVIELCNIVATRGARLAAAGVLGILKK
MGRDTPRQGGLEKTVVAMDGGLYEHYTEYRTCLENTLKELLGDELATSIVFEHSNDGSGI
GAALLAASNSMYLEDKS
>Nta|XP_016487727.1
MKKATVGAAVVGAATVCAVAAFIVNHRMRKSSKWARAMAILREFEEKCGTPDAKLKQVAD

AMTVEMHAGLASEGGSKLKMLITYVDNLPTGDEAGVFYALDLGGTNFRVLRVQLGGKDGG
IVHQEFAEASIPPNLMVGTSEALFDYIAAELAKFVAEEEEKFQQPPGKQRELGFTFSFPV
MQTSINSGTIMRWTKGFSIDDAVGQDVVGELTKAMKRKGVDMRVSALVNDTVGTLAGGKY
THNDVAVAVILGTGTNAAYVERVQAIPKWHGPVPKSGEMVINMEWGNFRSSHLPLTQYDH
ALDTNSLNPGDQIFEKMTSGMYLGEILRRVLLRVAEDAGIFGDEVPPKLKSPFVLRTPDM
SAMHHDISSDLRVVGDKLKDILEISNTSLKTRRLVVELCNIVATRGARLAAAGILGILKK
MGRDTPRQGGPEKTVVAMDGGLYEHYTEYRTCLENTLKELLGDELATSIVFEHSNDGSGI
GAALLAASNSMYLEDKS
>Nta|XP_016490297.1
MGKVVVGAAVVCTAAVCAAAVLLMRHKMKNSGKWAKAMDILKEFEEKCETPIGKLRQVAD
AMTVEMHAGLASEGGSKLKMLISYVDNLPTGDETGLFYALDLGGTNFRVMRVQLGGKEKR
IVKQEVKEVSIPKNVMAGSSSDALFDFIATALVKFVATEDDDFRLPPGRQRELGFTFSFP
VKQLSIASGTLIKWTKGFSIDDTVGQDVVRELTKAMERVGLDVRVAALVNDTVGTLAGGR
YNNPDVIAAVILGTGTNAAYVERANTIPKWHGLLPKSGDMVINMEWGNFRSSHLPVTEYD
QSLDTESLNPGEQIYEKMISGMYLGEILRRVLCRMAEEASFFDDYVPPKLKTPFILRTPD
MSAMHHDKSADLKVVGDKLKDILEVPNSTWKMRKIIVELCDIITSRGARLSAAGIVGILK
KLGRDTLKDGEKQRSVIAVDGALFEHYTKFRNCMEGTIKELLGDAAESIVIELSNDGSGI
GAALLAASHSQYLGLEES
>Nta|XP_016497279.1
MSVTVSSPAGRSFHISRSHTKMVSKPRVIMAAVRSGVSLGVVPILTKLQKDCATPLPVLR
HVADAMAVDMRAGLAVDGGSDLKMILSYVDTLPTGNEKGLFYALDLGGTNFRVLRVQLGG
KEERVIATEFEQVSIPQELMFATSEELFDFIASELGKFSQSEGGKFEMQQGRTREIGFTF
SFPVKQTSVKSGILIKWTKGFAVSGTAGKDVVACLNEAMERRGLGMQVSALVNDTVATLA
GARYWDNDVMVAVILGTGTNACYVERVDAIPKLPKRMSNSPVTIVNTEWGAFSNGLPLTE
FDREMDAESINPGEQIFEKTISGMYLGEIVRRVLVKMAKVGGLFGGGYVPEKLVTPFVLR
TPDICAMQQDTSRDLEAVESVLYDIAGVKSDLNARKTVVDICDTIAKRGGRLAGAGIVGI
LQKMEEDSKSVVFGKRTVVAMDGGLYEHYPQYREYIQEAVTELLGSEISKNVVIEHSKDG
SGIGAALLAAANSKYEHDY
>Nta|XP_016501284.1
MRKEVVVLAAVTTVSTVVAAALLVRQWKRRSEQRWRHAQRILRKFARECATPVPKLWQIA
DDLVTEMQAGLTSSESSLQMLPSCLASLPTGDEKGLYYGINLRGTNFIIVQARLGGRNEP
PVSRLAGRNEPISDLYRQEIQIPPNIIEGSSQELFDWITVELGKFISMHSEGLQGGEKNV
GFTVSPTIAEVAASKETAIKWKDSLFGDAEGNKLLNEINLALEKHGVDKQVFALVDDTIG
VLAGGRYYSKESVAAVTLGMGTNAVYVESAKSVEKWPDQAPKPEEIAINIKWGNFSSSHL
PITEFDTSLDAESSYPGSQIFEKLISGTYLGETVRRVLLKMAQESSLFGDRVPPKLAITY
SLRSPDMAAMHQDTSEDYEVIDEKLTEIFGITNSTTMARELVAEVCDVVAERGARLVGAG
IVGIVKKLGRLSNRISIITVEGGVYEHYRVFRNYLHSSVWEMLGNELSDNVIIEHSHGGS
GASSIFIAASQP
>Osa|XP_015614778.1
MRKAAALASAAMAAAAVAVVSTVLHQRQRRAAKRSERAEAVLLRDLQERCAAPVELLRQV
ADAMAAEMRAGLAAEGGSDLQMLVTYVDSLPSGGEKGMFYALDLGGTNFRVLRVQLGGKE
RRIIKQDSEGISIPQHLMSSSSHELFDFVAVALAKFVASEGEDCHLPEGTQRELGFTFSF
PVKQKSLASGTLIKWTKSFAIDEMVGKDVVAELNMAIRSQGLDMKVTALVNDTVGTLAAG
RYVNHDTIAAVILGTGSNAAYIDHADAIPKWHGSLPKSGNMVINMEWGNFKSSHLPLTEF
DQELDAESLNPGKQVYEKSISGMYMGELVRRILLKMAQETRIFGDNIPPKLERPYILRTL
DMLIMHHDTSSDLRTVANKLKEVLGIEYTSFTTRKLVLDVCEAIATRGARLAAAGIYGII
QKLGQHSDSPSTRRSVIAVDGGVYKYYTFFSQCMESTLSDMLGQELAPSVMIKHVNDGSG
VGAALLAASYSQYHQAESADSS
>Osa|XP_015614785.1
MFYALDLGGTNFRVLRVQLGGKERRIIKQDSEGISIPQHLMSSSSHELFDFVAVALAKFV
ASEGEDCHLPEGTQRELGFTFSFPVKQKSLASGTLIKWTKSFAIDEMVGKDVVAELNMAI
RSQGLDMKVTALVNDTVGTLAAGRYVNHDTIAAVILGTGSNAAYIDHADAIPKWHGSLPK
SGNMVINMEWGNFKSSHLPLTEFDQELDAESLNPGKQVYEKSISGMYMGELVRRILLKMA
QETRIFGDNIPPKLERPYILRTLDMLIMHHDTSSDLRTVANKLKEVLGIEYTSFTTRKLV

LDVCEAIATRGARLAAAGIYGIIQKLGQHSDSPSTRRSVIAVDGGVYKYYTFFSQCMEST
LSDMLGQELAPSVMIKHVNDGSGVGAALLAASYSQYHQAESADSS
>Osa|XP_015618116.1
MGKGTVVGTAVVVCAAAAAAVGVAVVVSRRRRSKREAEEERRRRAAAVIEEVEQRFSTPT
ALLRGIADAMVEEMERGLRADPHAPLKMLISYVDNLPTGDEHGLFYALDLGGTNFRVIRV
QLGGREKRVVSQQYEEVAIPPHLMVGTSMELFDFIAAELESFVKTEGEDFHLPEGRQREL
GFTFSFPVHQTSISSGTLIKWTKGFSINGTVGEDVVAELSRAMERQGLDMKVTALVNDTV
GTLAGGRYVDNDVAAAVILGTGTNAAYVEHANAIPKWTGLLPRSGNMVINMEWGNFKSER
LPRSDYDNALDFESLNPGEQIYEKMISGMYLGEIVRRILLKLAHDASLFGDVVPTKLEQR
FILRTPDMSAMHHDTSHDLKHLGAKLKDILGVADTSLEARYITLHVCDLVAERGARLAAA
GIYGILKKLGRDRVPSDGSQKQRTVIALDGGLYEHYKKFRTCLEATLADLLGEEAASSVV
VKLANDGSGIGAALLAASHSQYASVE
>Osa|XP_015621344.1
MGRVGLGVAVGCAAVTCAIAAALVARRASARARWRRAVALLREFEEGCATPPARLRQVVD
AMVVEMHAGLASDGGSKLKMLLTFVDALPSGSEEGVYYSIDLGGTNFRVLRVQVGAGSVI
VNQKVEQQPIPEELTKGTTEGLFNFVALALKNFLEGEDDQDGKMALGFTFSFPVRQISVS
SGSLIRWTKGFSIRDTVGRDVAQCLNEALANCGLNVRVTALVNDTVGTLALGHYYDEDTV
AAVIIGSGTNACYIERTDAIIKCQGLLTNSGGMVVNMEWGNFWSSHLPRTPYDILLDDET
HNRNDQGFEKMISGMYLGEIARLVFHRMAQESDVFGDAADSLSNPFILSTPFLAAIREDD
SPDLSEVRRILREHLKIPDAPLKTRRLVVKVCDIVTRRAARLAAAGIVGILKKLGRDGSG
AASSGRGRGQPRRTVVAIEGGLYQGYPVFREYLDEALVEILGEEVARNVTLRVTEDGSGV
GAALLAAVHSSNRQQQGGPI
>Osa|XP_015637554.1
MVAAAVAAAEQVVAALREECATPAARLDGVAAAMAGEMAAGLAEEGGSKIKMIVSYVDNL
PNGTEEGLFYALDLGGTNFRVLRVQLAGKEKRVVKRESREVSIPPHLMSGNSSELFGFIA
SALAKFVADEGHNAVFNDRQRELGFTFSFPVRQTSIASGTLIKWTKAFSIDDAVGEDVVA
ELQMAMEKQGLDMRVSALINDTVGTLAAGSYYDEDIVVGVILGTGSNAAYLEKANAIPKL
EGELPKSGNMVINTEWGNFSSSCLPITEYDEALDKESLNPGEQIFEKLISGMYLGEIVRR
VLLKISLQSSIFGNLDQTKLKTRFILRTPDISVMHHDGTPDLRIVAEKLADNLKITDTSL
ETRKMVVEICDIVTRRSARLAAAGIVGILRKIGRGVPGDKRKSVIAIDGGLYEHYTEFRQ
CLETTLTELLGEEASKSVAVKLANDGSGLGAALIAAAHSQYLN
>Osa|XP_015637797.1
MRKAAAAAVAAAAAVGVALLVRRQLREAKRWGRADAVLRELEERCAAPPGRLRQVADAMA
VEMHAGLASEGGSKLKMIISYVDALPSGEEKGVFYALDLGGTNFRVLRVQLGGKEGRVIK
QEHDEISIPPHLMTGGSNELFDFIASSLAKFVASEGEDFHLAEGRQRELGFTFSFPVKQT
SIASGTLINWTKGFSIDETVGEDVVTELTKALERQGLDMKVTALINDTIGTLAGGRYDDN
DVIAAVILGTGTNAAYVERANAIPKWHDLLPKSGDMVINMEWGNFRSSHLPLTEFDQALD
AESLNPGEQVYEKLISGMYLGEIVRRVLLKMAEEASLFGDEVPPKLKIPFIIRTPYMSMM
HCDRSPDLRTVGAKLKDILGVQNTSLKTRRLVVDVCDIVAKRAAHLAAAGIHGILKKLGR
DVPNTDKQRTVIAVDGGLYEHYTIFAECVESTLRDMLGEDVSSTIVIKLAKDGSGIGAAL
LAAAHSQYREAEEL
>Osa|XP_015639323.1
MGKAAAVGTAVVVAAAVGVAVVLARRRRRRDLELVEGAAAERKRKVAAVIEDVEHALSTP
TALLRGISDAMVTEMERGLRGDSHAMVKMLITYVDNLPTGNEQGLFYALDLGGTNFRVLR
VQLGGKEKRVVQQQYEEVSIPPHLMVGTSMELFDFIASALSKFVDTEGDDFHLPEGRQRE
LGFTFSFPVSQTSISSGTLIKWTKGFSINDAVGEDVVSELGKAMERQGLDMKIAALVNDT
VGTLAGGRYADNSVVAAIILGTGTNAAYVENANAIPKWTGLLPRSGNMVINTEWGSFKSD
KLPLSEFDKAMDFESLNPGEQIYEKLISGMYLGEIVRRILLKLAHDAALFGDVVPSKLEQ
PFVLRTPDMSAMHHDSSHDLKTVGAKLKDIVGVPDTSLEVRYITSHICDIVAERAARLAA
AGIYGVLKKLGRDKMPKDGSKMPRTVIALDGGLYEHYKKFSSCLESTLTDLLGDDVSSSV
VTKLANDGSGIGAALLAASHSQYAEID
>Osa|XP_015645316.1
MSAAAAIASPIPAAIAVVQQQRRGRSRGGGSGAAAVRCSAVAPTSAIAPILADLRLRCAA
PLPVLRRVADAMASGMRAGLADDGAGELKMIPSHVYSLPTGNETGLFYALDLGGTNFRVL

RVQLGGKDKRIIDTEFEQVSIPREIMHGITEDLFDFIASGLSRFVATEGDKFHLPQGRKR
ELGFTFSFPVNQTSIDSGILIKWTKGFAVSGTAGKDVVACLNAAMERQGLDMRVSALVND
TVGTLAGARYWDDDVMVAVILGTGTNACYIQRTEAIPKLQHLKLETGNTIINTEWGAFSD
GLPLTEFDREMDDESINPGEQIFEKTISGMYLGEIVRRVLVKMAEVSDLFGHSFPKKLAE
PFVLRTPHLCAMQQDTSDNLGEVESILSDVIGVSQASLLARRVTVEVSDCIIRRGGRLAG
AGIVGILEKMENDSRGHIFGRRTVVAMDGGLYEKYPQYRRYMKEAVAELLGPERSNRIAI
EHTKDGSGIGAALLAAANSKYAAAQISTR
>Ppa|XP_024359760.1
MGQSKVLVGVYIACAAAACATAAVAVTQRLKVRAQKCTARKILVEFQEACETPLPRLRQV
VDAMAVEMHAGLVSEGGSKLKMLPTFIDRLPNGSEKGLYYAVDLGGTNFRVLRVQLGGLE
GRVIKQEYEEVAIPPELMLGTSEQLFHFIAKELAGFVAREGEEFRLGDGQSREIGFTFSF
PCKQTAVNSGTLLQWTKGFKVNDAIGQDVVAALQKCIERLGCKMRIAALVNDTVGTLAGG
RYWNNDVMIAVILGTGTNACYVERAESISKWTGELPKSGQMVINMEWGNFWSSHLPRTYV
DELLDSESLHPGEYGFEKMISGMYLGDCVRRVLVRMAQEAGIFGPHVPHTLLESFSLQTP
EMSRMHHDDSSDLKVVAEVLKRLYGIQNTTVGIRKIVVAVCDTTCQRGARLAAAGIVGIL
KKIGRDGSTANGLMRRNDTNGIHDELSVNSTPGSGKTVVAMDGGLYEHYSKFRNYMQEAV
RELLGDASKNVSIELSKDGSGIGAALLAASYAEYVPS
>Ppa|XP_024360110.1
MADAFKARYSSGAQIRTGKIRSAAACAAAAVIVSRRLKVRSQKCTARKILLEFQEACYTP
LARLRQVVDAMAVEMHAGLVSEGGSKLKMLPTYIDRLPDGHERGLYYAVDLGGTNFRVLR
VQLGGLEGRVIKQEYEEVAIPPELMLGTSEQLFDFIAKELVSFVAREGQDFRLHAGQNRE
IGFTFSFPVKQTAVNSGTLLQWTKGFKVNDAVGEDVVAALQRGIERRGYKMRIAALVNDT
VGTLAGGRYWNNDVMIGVILGTGTNACYVERAEAVSKWAGDIPKSGEMVINMEWGNFWSS
HLPRTYVDESLDNESLNPGEYGFEKMISGMYLGDCVRRVLVRMAQEACIFGTPVPHKLLE
AFSLMTPDMSKMHHDDSSDLKVVAEVLKRVYGIQNTTVGIRKIVVAVCDTVCQRGARLAA
AGIVGILKKIGRDGSAANGVIKRNTFEQSDMNGFHDEVPVHYTSGGRTVVAMDGGLYEHY
TKFRNYMQEAVVELLGEGSKNVVIELSKDGSGIGAALLAASHAEYVIS
>Ppa|XP_024401777.1
MGQSKAMAGVYIACAAAACAAAAVVLTQRVKVRSQKYTARKILVEFQEACETPLPRLRQV
VDAMAVEMHAGLVSEGGSKLKMLPTFIDHLPDGNEKGLYYAVDLGGTNFRVLRTQLGGLE
GRVIKQEYEEVAIPPELMLGTSEQLFDFIAKELVSFVAREGEDFRLHEGQSREIGFTFSF
PCKQTAVNSGTLLQWTKGFKVNDAIGQDVVAALQGSIERRGYKMRIAALVNDTVGTLAGG
RYWNNDVMIAVILGTGTNACYVERAESISKWGGELPKSGQMVINMEWGNFWSSHLPRTYV
DELLDNESLNPGEYGFEKMISGMYLGDCVRRVLVRMAQQAGIFGPHVPHKLLEAFTLQTP
DMSKMHHDDSSDLKMVAEVLKTVYGIHNTTLGIRKIVLAVCDIVCQRGARLAAAGIVGIL
KKIGRDGSTANGFIRRNDVNGIHDELTVNSIGGSGKTVVAMDGGLYEHYSKFRNYMQQAV
RELLGDAAKNVFIELSKDGSGIGAAILAASHAEYVPT
>Par|CAB4265578.1
MSVAATSPAVGSFYLRRRSPSTAMSMPRFRMAVRSAAVSVTPLLTKLHNDSATPLPLLRH
VAEAMAADMRAGLAVEGGSDLKMILSYVDSLPSGNEEGLFYALDLGGTNFRVLRVQLGGK
QERVIATEFDQVSIPKELMFGTSEQLFDFIASGLAKFAQKEGNKFHLPTGTKREIGFTFS
FPVKQTSIDSGILIKWTKGFSVSGTAGKDVVACLNEAMERQGLDMRVSALVNDAVGTLAG
ARYWDDDVMVAVILGTGTNACYVERADAIPKLQGQVSSSGRTIINTEWGAFSDGLPLTEF
DREMDAASINPGEQIFEKTISGMYLGEIVRRVLVKMAEADALFGESVPEKLSTPFALRTP
DICAMQQDISDDLQDVGSILYNVAGVESNLSARKIVVDVCDTIVKRGGRLAGAGIVGILQ
KMEEDSKGLIFGKRTVVAMDGGLYANYPQYRRYLQEAVTELLGTEISKNVVIELTKDGSG
IGAALLAAANSQYA
>Par|CAB4272496.1
MGKKAVVIITAAAVCAAAAALVVRHHMRSSGRWARATAIIKELEEKCGTPIGKLRQVADA
MAVEMHAGLASEGGSKLKMIISYVDNLPTGTEKGLFYALDLGGTNFRVLRVQLGGKGRGI
ISQEFTEVSIPEHLMVGTSDALFDYIAAELAKFVAKEGQDYQLPPGRQRELGFTFSFPVL
QSSINSGTLIKWTKGFSIDDAVGQDVVAELSKAIEKQGLDMRVSALVNDTVGTLAGGRYV
NGDVVAAVILGTGTNAAYVERAHAIPKWHGLLPKSGDMVINMEWGSFKSAHLPLTEYDHS
LDTESLNPGDQIFEKLISGMYLGEIVRRVLCKIAEEASLFGDTVPPKLKVPFILRTPDMS

AMHHDTSSDLKVVRDILKNVLEISNTSLKVRKFVVELCNIVATRGARLAAAGIVGVLKKV
GKDAFKDGEKQRTVVALDGGLYEHYNEYSKCMENTLRELLGEEVSESIVIEHSNDGSGIG
AALLAASHSQYLGVDES
>Par|CAB4278350.1
MGKVAVGLAVGVAVAACAVATVVVGRRIRSRRKWRRVVGVLKELEQSCETTVGRLRQVVD
AMAVEMHAGLASDGGSKLKMLLTFVDKLPNGSEKGTYYALDLGGTNFRVLRVKLEGTRSS
TLEHDVYRQAIPQDLMTGSSEDLFDFIASSLKEFVEREGDISELSLDRRRELGFTFSFPV
KQMSVSSGTLLKWTKGFSIEDMVGREIAECLENAMTRKGLNMRVAALVNDSVGSLALGHY
HDTDTVAAVIIGTGTNACYLERTDAIIKSQGLLTTSGGMVVNMEWGNFWSSHLPRTLYDI
ELDADSPNPNDQGFEKLISGMYLGDVVRRVIVRMSQESDIFDPISSKLSMPFILRTPLIA
AMHEDDSPDLEEVRRILRDNLEIPDVPLKVRKLIVKVCDVVTRRAARLAAAGIVGILKKI
GRDGSGGIAGGRSRSDQKMRRTVVAMEGGLYTGYTMFRDYLHEALTEILGEDIAQHVIIK
VTEDGSAMGAALLAASNSSYPVDSVQLL
>Par|CAB4283983.1
MRKEVVVAAALTATATVVVAAALLRQWKRKKQRQWRETQKILRKFARDCATPVPKLWLVS
NALVSDIKACLASSGTITTLNMLVSYVAPLPSGDEEGLYYGVNLRGTNFLILCARLGGKN
KPISDLYREEIPIPSNLMAATSKELFDFVAVELGKFISEHPDGQADSAEGNKLGCIVSCP
VDQAVVSHGTAIKWKSFSADSKVGKKLVSDFNQALEEHGVNLRVYAMVDDTVGNLAGGRY
YNKESVAAVTLAMGTDAAYVEPADAALQWHGPSPKLGEMVISTQWGDFSSPHLPITIFDT
CLDAESSNPGRRRFEKLISGMYLGEIVRRVLLKMAQETALFGDHVPSKLMTPYQLSSPDM
AAMHQDASDDREIVGEKLTKVFGITSTSPRVREVVSEVCDITAERGARLAGAGILGIIKK
LGRIENKKSVVTVEGGLYEHYRVFRNYLNSSVWEMLGNDLSDNVVIEHSHGGSGTGALFL
AASQMDMQNDEPDPDNDNDDPDSDNNDHDHDPDLDADT
>Par|CAB4288362.1
MGKVAVGAAVVCAAAVCAAAALVVRHRMKSSGRWARAMAILREFEDKCGTPIGKLRQVAD
AMTVEMHAGLASEGGSKLKMLISYVDNLPTGDEQGLFYALDLGGTNFRVIRVQLGGKEKR
VVKQEFDEVSIPPNLMTGTSEALFDFIAEALAKFVATEGEGFHPAPGRQRELGFTFSFPV
WQTSIASGTLIKWTKGFNIEDAVEQDVVGELTKSVEKIGLDMRVTALVNDTIGTLAGGRY
HNQDVIAAVILGTGTNAAYVERAHAIPKWHGLLPKSGEMVINMEWGNFRSSHLPLTEYDQ
ALDAESLNPGDQIFEKIISGMYLGDIVRRVLCKMAEEASFFGDVVPPKLKVPFILRTPDM
SAMHHDTSPDLRVVGSKLKDILEISNTSLKMRKVVVALCDIVATRGARLSAAGIMGVLKK
LGRDTVKEGEKQKSVVALDGGLYEHYTEFRTSMESTLKELLGDEVAEHISVEHSNDGSGI
GAALLAASHSQYLEVEES
>Pav|XP_021800069.1
MGKKAVVIITAAAVCAAAAALVVRHRMRSSGRWARATAIIKELEEKCGTPIGKLRQVADA
MAVEMHAGLASEGGSKLKMIISYVDNLPTGTEKGLFYALDLGGTNFRVMRVQLGGKGRGI
ISQEFTEVSIPENLMVGTSYALFDYIAAELAKFVAKEGQDYQLPPGRQRELGFTFSFPVL
QSSINSGTLIKWTKGFSIDDAVGQDVVAELSKAIEKQGLDMRVSALVNDTVGTLAGGRYV
NGDVVAAVILGTGTNAAYVERAHAIPKWHGLLPKSGDMVINMEWGNFKSAHLPLTEYDHS
LDTESLNPGDQIFEKLISGMYLGEIVRRVLCKIAEEASLFGDTVPPKLKVPFILRTPDMS
AMHHDTSSDLKVVRDILKNVLEISNTSLKVRKVVVELCNIVATRGARLAAAGIVGVLKKV
GKDALKDGEKQRTVVALDGGLYEHYNEYSKCMENTLRELLGEEVSESVVIEHSNDGSGIG
AALLAASHSQYLGVDES
>Pav|XP_021807184.1
MGKVAVGAAVVCAAAVCAAAALVVRHRMKSSGRWARAMAILREFEDKCGTPIGKLRQVAD
AMTVEMHAGLASEGGSKLKMLISYVDNLPTGDEQGLFYALDLGGTNFRVLRVQLGGKEKR
VVKQEFDEVSIPPNLMTGTSEALFDFIAETLAKFVATEGEGFHPAPGRQRELGFTFSFPV
WQTSIASGTLIKWTKGFNIEDAVEQDVVGELTKSVEKIGLDMRVTALVNDTIGTLAGGRY
HNQDVIAAVILGTGTNAAYVERAHAIPKWHGLLPKSGEMVINMEWGNFRSSHLPLTEYDQ
ALDAESLNPGDQIFEKIISGMYLGDIVRRVLCKMAEEASFFGDVVPPKLKVPFILRTPDM
SAMHHDTSSDLRVVGSKLKDILEISNTSLKMRKVVVALCDIVATRAARLSAAGIMGVLKK
LGRDTVKEGEKQKSAVALDGGLYEHYTEFRTSMESTLKELLGDEVAEHISVEHSNDGSGI
GAALLAASHSQYLEVEES
>Pav|XP_021810618.1

MGKVAVGLAVGVAVAACAVATVVVGRRIRSRRKWRRVVGVLKELEQSCETTVGRLRQVVD
AMAVEMHAGLASDGGSKLKMLLTFVDKLPNGSEKGTYYALDLGGTNFRVLRVKLEGTRSS
TLEHDVYRQPIPQDLMTGSSEDLFDFIASSLKEFVEREGDISELSLDRRRELGFTFSFPV
KQMSVSSGTLLKWTKGFSIEDMVGREIAGCLEKAMTRKGLNMRVAALVNDSVGSLALGHY
HDTDTVAAVIIGTGTNACYLERTDAIIKSQGLLTTSGGMVVNMEWGNFWSSHLPRTVYDI
ELDADSPNPNDQGFEKLISGMYLGDVVRRVIVRMSQESDIFDPISSKLSMPFILRTPLIA
AMHEDDSPDLEEVRRILRDNLEIPDVPLKVRKLIVKVCDVVTRRAARLAAAGIVGILKKI
GRDGSGGIAGGRSRSDQKMRRTVVAMEGGLYTGYTMFRDYLHEALTEILGEDIAQHVIIK
VTEDGSAMGAALLAASHSSYPVDSVQLL
>Pav|XP_021826638.1
MSVAATSPAVGSFYLRRRSPSTAMSMPRFRMAVRSAAVSVTPLLTKLHNDSATPLPLLRH
VADAMAADMRAGLAVEGGSDLKMILSYVDSLPSGNEEGLFYALDLGGTNFRVLRVQLGGK
QERVIATEFDQVSIPKELMFGTSEQLFDFIAAGLAKFAQKEGNKFHLPTGTKREIGFTFS
FPVKQTSIDSGILIKWTKGFSVSGTAGKDVVACLNEAMERQGLDMRVSALVNDTVGTLAG
ARYWDDDVMVAVILGTGTNACYVERADAIPKLQGQVSSSGKTIINTEWGAFSDGLPLTEF
DREMDAASINPGEQIFEKTISGMYLGEIVRRVLVKMAEADALFGESVPEKLSTPFVLRTP
DICAMQQDISDDLQDVGSILYNVAGVESNLSARKIVVDVCDTIVKRGGRLAGAGIVGILQ
KMEEDSKDLIFGKRTVVAMDGGLYENYPQYRRYLQEAVTELLGTEISKNVVIEHSKDGSG
IGAALLAAANSQYT
>Pav|XP_021829525.1
MRKEVLVAAALTATATVVVAAALLRQWKREKQRQWRETQKILRKFARDCATPVPKLWLVS
NALVSDVKACLASSGTITTLNMLVSYVAPLPSGDEEGFYYGVNLRGTNFLILCARLGGKN
KPISDLHREEIPIPSNLMAATSKELFDFVAVELGKFISEHPDGQADSAEENKLGCIVSCP
VDQAVVSHGTAIKWKSFAADSKVGKKLVSDFNQALEEHGVNLRVYAMVDDTVGNLAGGRY
YTKESVAAVTLAMGTDAAYVEPADAALQWHGPSPKLGEMVISTQWGDFSSPHLPITIFDT
CLDAESSNPGRRRFEKLISGMYLGEIVRRVLLKMAQETALFGDHVPSKLMTPYQLSSPDM
AAMHQDASDDREIVGEKLRKVFEITSTSPRVREVVSEVCDIIAERGARLAGAGILGIIKK
LGRIENKKSVVTVEGGLYEHYRVFRNYLNSSVWEMLGNDLSDNVVIEHSHGGSGTGALFL
AASQMDMQNDDPDPDNDNDDPDSDNNDHDPDPDADT
>Pdu|XP_034201006.1
MSVAATPPAVGPFYLRRRSPSTAMSMPRFRMAVRSAAVSVTPLLTKLHNDSATPLPLLCH
VADAMAADMRAGLAVDGGSDLKMILSYVDSLPSGNEEGLFYALDLGGTNFRVLRVQLGGK
QERVIATEFDQVSIPKELMFGTSEQLFDFIASGLAKFAQKEGNKFHLPTGTKREIGFTFS
FPVKQTSIDSGILIKWTKGFSVSGTAGKDVVACLNEAMERRGLDMRVSALVNDAVGTLAG
ARYWDDDVMVAVILGTGTNACYVERADAIPKLQGQVSSSGRTIINTEWGAFSDGLPLTEF
DREMDAASINPGEQIFEKTISGMYLGEIVRRVLVKMAEADALFGESVPEKLSTPFALRTP
DICAMQQDISDDLQDVGSILYNVSGVESNLSARKIVVHVCDTIVKRGGRLAGAGIVGILK
KMEEDSKGLIFGKRTVVAMDGGLYENYPQYRRYLQEAVTELLGTEISKNVVIEHSKDGSG
IGAALLAAANSQYA
>Pdu|XP_034207972.1
MGKKAVVIITAAAVCAAAAALVVRHRMRSSGRWARATAIIKELEEKCGTPIGKLRQVADA
MAVEMHAGLASEGGSKLKMIISYVDNLPTGTEKGLFYALDLGGTNFRVLRVQLGGKGRGI
ISQEFTEVSIPENLMVGTSDALFDYIAAELAKFVAKEGQDYQLPPGRQRELGFTFSFPVL
QSSINSGTLIKWTKGFSIDDAVGQDVVAELSKAIEKQGLDMRVSALVNDTVGTLAGGRYV
NGDVVAAVILGTGTNAAYVERAHAIPKWHGLLPKSGDMVINMEWGNFKSAHLPLTEYDHS
LDTESLNPGDQIFEKLISGMYLGEIVRRVLCKIAEEASLFGDTVPPKLKVPFILRTPDMS
AMHHDTSSDLKVVRDILKNVLEISNTSLKVRKIVVELCNIVATRGARLAAAGIVGVLKKV
GKDAFKDGEKQRTVVALDGGLYEHYNEYSKCMENTLRELLGEEVSESIVIEHSNDGSGIG
AALLAASHSQYLGVDES
>Pdu|XP_034211665.1
MGKLAVGLAVGVAVAACAVATVVVGRRIRSRRKWRRVVGVLKELEQSCETTVGRLRQVVD
AMAVEMHAGLASDGGSKLKMLLTFVDKLPNGSEKGTYYALDLGGTNFRVLRVKLEGTRSS
TLEHDVYRQAIPQDLMTGSSEDLFDFIASSLKEFVEREGDISELSLDRRGELGFTFSFPV
KQMSVSSGTLLKWTKGFSIEDMVGREIAGCLENAMTRKGLNMRVAALVNDSVGSLALGHY

HDTDTVAAVIIGTGTNACYLERTDAIIKSQGLLTTSGGMVVNMEWGNFWSSHLPRTLYDI
ELDADSPNPNDQGFEKLISGMYLGDVVRRVIVRMSQESDIFDPISSKLSMPFILRTPLIA
AMHEDDSPDLEEVRRILRDNLEILDVPLKVRKLIVKVCDVVTRRAARLAAAGIVGILKKI
GRDGSGGIAGGRSRSDQKMRRTVVAMEGGLYTGYTMFRDYLHEALTEILGEDIAQHVIIK
VTEDGSAMGAALLAASHSSYPVDSVQLL
>Pdu|XP_034223332.1
MGKVAVGAAVVCAAAVCAAAALVVRHRMKCSGRWARAMAILREFEDKCGTPIGKLRQVAD
AMTVEMHAGLASEGGSKLKMLISYVDNLPTGDEQGLFYALDLGGTNFRVIRVQLGGKEKR
VVKQEFDEVSIPPNLMTGTSEALFDFIAEALAKFVATEGEGFHPAPGRQRELGFTFSFPV
WQTSIASGTLIKWTKGFNIEDAVEQDVVGELTKSVEKIGLDMRVTALVNDTIGTLAGGRY
HNQDVIAAVILGTGTNAAYVERAHAIPKWHGLLPKSGEMVINMEWGNFRSSHLPLTEYDQ
ALDAESLNPGDQIFEKIISGMYLGDIVRRVLRKMAEEASFFGDVVPPKLKVPFILRTPDM
SAMHHDTSPDLRVVGSKLKDILEISNTSLKMRKVVVALCDIVATRGARLSAAGIMGVLKK
LGRDTVKEGEKQKSVVALDGGLYEHYTEFRTSMESTLKELLGDEVAEHISVEHSNDGSGI
GAALLAASHSQYLEVEES
>Pdu|XP_034223333.1
MGKVAVGAAVVCAAAVCAAAALVVRHRMKCSGRWARAMAILREFEDKCGTPIGKLRQVAD
AMTVEMHAGLASEGGSKLKMLISYVDNLPTGDEQGLFYALDLGGTNFRVIRVQLGGKEKR
VVKQEFDEVSIPPNLMTGTSEVEQDVVGELTKSVEKIGLDMRVTALVNDTIGTLAGGRYH
NQDVIAAVILGTGTNAAYVERAHAIPKWHGLLPKSGEMVINMEWGNFRSSHLPLTEYDQA
LDAESLNPGDQIFEKIISGMYLGDIVRRVLRKMAEEASFFGDVVPPKLKVPFILRTPDMS
AMHHDTSPDLRVVGSKLKDILEISNTSLKMRKVVVALCDIVATRGARLSAAGIMGVLKKL
GRDTVKEGEKQKSVVALDGGLYEHYTEFRTSMESTLKELLGDEVAEHISVEHSNDGSGIG
AALLAASHSQYLEVEES
>Ppe|XP_007203872.1
MGKVAVGAAVVCAAAVCAAAALVVRHRMKCSGRWARAMAILREFEDKCGTPIGKLRQVAD
AMTVEMHAGLASEGGSKLKMLISYVDNLPTGDEQGLFYALDLGGTNFRVIRVQLGGKEKR
VVKQEFDEVSIPPNLMTGTSEALFDFIAEALAKFVATEGEGFHPAPGRQRELGFTFSFPV
WQTSIASGTLIKWTKGFNIEDAVEQDVVGELTKSVEKIGLDMRVTALVNDTIGTLAGGRY
HNQDVIAAVILGTGTNAAYVERAHAIPKWHGLLPKSGEMVINMEWGNFRSSHLPLTEYDQ
ALDAESLNPGDQIFEKIISGMYLGDIVRRVLCKMAEEASFFGDVVPPKLKVPFILRTPDM
SAMHHDTSPDLRVVGSKLKDILEISNTSLKMRKVVVALCDIVATRGARLSAAGIMGVLKK
LGRDTVKEGEKQKSVVALDGGLYEHYTEFRTSMESTLKELLGDEVAEHISVEHSNDGSGI
GAALLAASHSQYLEVEES
>Ppe|XP_007211826.1
MGKVAVGLAVGVAVAACAVATVLVGRRIRSRRKWRRVVGVLKELEQSCETTVGRLRQVVD
AMAVEMHAGLASDGGSKLKMLLTFVDKLPNGSEKGTYYALDLGGTNFRVLRVKLEGTRSS
TLEHDVYRQAIPQDLMTGSSEDLFDFIASSLKEFVEREGDISELSLDRRGELGFTFSFPV
KQMSVSSGTLLKWTKGFSIEDMVGREIAGCLENAMTRKGLNMRVAALVNDSVGSLALGHY
HDTDTVAAVIIGTGTNACYLERTDAIIKSQGLLTTSGGMVVNMEWGNFWSSHLPRTLYDI
ELDADSPNPNDQGFEKLISGMYLGDVVRRVIVRMSQESDIFDPISSKLSMPFILRTPLIA
AMHEDDSPDLEEVRRILRDNLEILDVPLKVRKLIVKVCDVVTRRAARLAAAGIVGILKKI
GRDGSGGIAGGRSRSDQKMRRTVVAMEGGLYTGYTMFRDYLHEALTEILGEDIAQHVIIK
VTEDGSAMGAALLAASHSSYPVDSVQLL
>Ppe|XP_007215785.2
MGKKAVVIITAAAVCTAAAALVVRHRIRSSGRWARATAIIKELEEKCGTPIGKLRQVADA
MAVEMHAGLASEGGSKLKMIISYVDNLPTGTEKGLFYALDLGGTNFRVLRVQLGGKGRGI
ISQEFTEVSIPENLMVGTSDALFDYIAAELAKFVAKEGQDYQLPPGRQRELGFTFSFPVL
QSSINSGTLIKWTKGFSIDDAVGQDVVAELSKAIEKQGLDMRVSALVNDTVGTLAGGRYV
NGDVVAAVILGTGTNAAYVERAHAIPKWHGLLPKSGDMVINMEWGNFKSAHLPLTEYDHS
LDTESLNPGDQIFEKLISGMYLGEIVRRVLCKIAEEASLFGDTVPPKLKVPFILRTPDMS
AMHHDTSSDLKVVRDILKNVLEISNTSLKVRKIVVELCNIVATRGARLAAAGIVGVLKKV
GKDAFKDGEKQRTVVALDGGLYEHYNEYSKCMENTLRELLGEEVSESIVIEHSNDGSGIG
AALLAASHSQYLGVDES

>Ppe|XP_007222247.1
MSVAATPPAVGPFYLRRRSPSTAMSMPRFRMAVRSAAVSVTPLLTKLHNDSATPLPLLRH
VADAMAADMRAGLAVDGGSDLKMILSYVDSLPSGNEEGLFYALDLGGTNFRVLRVQLGGK
QERVIATEFDQQLFDFIASGLAKFAQKEGNKFHLPTGTKREIGFTFSFPVKQTSIDSGIL
IKWTKGFSVSGTAGKDVVACLNEAMERRGLDMRVSALINDAVGTLAGARYWDDDVMVAVI
LGTGTNACYVERADAIPKLQGQVSSSGRTIINTEWGAFSDGLPLTEFDREMDAASINPGE
QIFEKTISGMYLGEIVRRVLVKMAEADALFGESVPEKLSTPFALRTPDICAMQQDISDDL
QDVGSILYNVAGVESNLSARKIVVDVCDTVVKRGGRLAGAGIVGILQKMEEDSKGLIFGK
RTVVAMDGGLYENYPQYRRYLQEAVTELLGTEISKNVVIEHSKDGSGIGAALLAAANSQY
A
>Ppe|XP_007222248.1
MSVAATPPAVGPFYLRRRSPSTAMSMPRFRMAVRSAAVSVTPLLTKLHNDSATPLPLLRH
VADAMAADMRAGLAVDGGSDLKMILSYVDSLPSGNEEGLFYALDLGGTNFRVLRVQLGGK
QERVIATEFDQVSIPKELMFGTSEQLFDFIASGLAKFAQKEGNKFHLPTGTKREIGFTFS
FPVKQTSIDSGILIKWTKGFSVSGTAGKDVVACLNEAMERRGLDMRVSALINDAVGTLAG
ARYWDDDVMVAVILGTGTNACYVERADAIPKLQGQVSSSGRTIINTEWGAFSDGLPLTEF
DREMDAASINPGEQIFEKTISGMYLGEIVRRVLVKMAEADALFGESVPEKLSTPFALRTP
DICAMQQDISDDLQDVGSILYNVAGVESNLSARKIVVDVCDTVVKRGGRLAGAGIVGILQ
KMEEDSKGLIFGKRTVVAMDGGLYENYPQYRRYLQEAVTELLGTEISKNVVIEHSKDGSG
IGAALLAAANSQYA
>Ppe|XP_020420850.1
MRKEVVVAAALTATATVVVAAALLRQWKRKKQRQWRETQKILRKFARDCATPVPKLWLVS
NALDSDMKAFLASSGTITTLNMLVSYVASLPSGDEEGFYYGVNLRGTNFLILCARLGGKN
KPISDLFREEIPIPSNLMAATSKELFDFVAVELGKFISEHPDGQADSAEENKLGCIVSCP
VDQAVVSHQTAIKWKSFSADSKVGKKLVSDFNQALEEHGVNLRVYAIQVDDTVGNLAGGR
YYNKESVAAVTLAMGTDAAYVEPADAALQWHGPSPKLGEMVISTQWGDFSSPHLPITIFD
TCLDAESSNPGRRRFEKLISGMYLGEIVRRVLLKMAQETALFGDHVPSKLMTPYQLSSPD
MAAMHQDASDDREIVGEKLTKVFGITSTSPRVREVVSEVCDIIAERGARLAGAGILGIIK
KLGRIENKKSVVTVEGGLYEHYRVFRNYLNSSVWEMLGNDLSDNVVIEHSHGGSGTGALF
LAASQMDMQNDDPDLDNDNVDPDSDNNDPDHDPDPDADT
>Sly|NP_001233957.1
MKKVTVGVAVVGAAAVCAVAVLIVNHRMRKSSKWGRAMAILREFEEKCKTQDAKLKQVAD
AMTVEMHAGLASEGGSKLKMLITYVDNLPTGDEAGVFYALDLGGTNFRVLRVQLGGKDGG
IIHQEFAEASIPPSLMVGTSDELFDYIAAELAKFVAAEEEKFHQPPGKQRELGFTFSFPV
MQTSINSGNIMRWTKGFSIDDAVGQDVVGELTKAMKRKGVDMRVSALVNDTVGTLAGGKY
TQKDVAVAVILGTGTNAAYVERVQAIPKWHGPVPKSGEMVINMEWGNFRSSHLPLTEYDH
ALDNESLNPGEQIFEKMTSGMYLGEILRRVLLRVAEEAGVFGDEVPPKLKEPFVLRTPDM
SAMHHDTSSDLKVVGEKLKDILEISNTSLKTRKLVVELCNIVATRGARLAAAGVLGILKK
MGRDTPKQGGSERTVIAMDGGLYEHYTEYRMCLENSLKDLLGEELATSIVFVHSNDGSGI
GAALLAASHSMYLEDQDA
>Sly|NP_001234406.2
MKKATVGAVVVGTAAAVAVAALVMRHRMGKSSKWARARAILKEFEEKCATPDAKLKQVAD
AMTVEMHAGLASEGGSKLKMLISYVDNLPTGDEAGVFYALDLGGTNFRVLRVQLGGKDGG
IMHQEFAEASIPPNLMVGTSEALFDYIAAELAKFVDEEGEKFHPPPGKQRELGFTFSFPI
MQTSINSGTLIRWTKGFSIDDTVGKDVVAELTKAMQKREIDMRVSALVNDTVGTLAGGRF
TDKDVSIAVILGTGTNAAYVERAQAIPKWHGPLPNSGEMVINMEWGNFRSSHLPLTQYDN
AMDTDSLNPGEQIFEKICSGMYLGEILRRVLLRMAKEAGIFGEEVPPKLKNSFILRTPEM
SAMHHDTSSDLRVVGDKLKDILEISNTSLKTRRLVVELCNIVATRGARLAAAGILGIIKK
MGKDTPRESGPEKIVVAMDGGLYEHYTEYSKCLENTLVELLGKEMATSIVFKHANDGSGI
GAALLAASNSVYVEDK
>Sly|NP_001234710.1
MGKLVVGATVVCTAAVVCGVTVLLMKHRVKNSGEWGKVEALLKDFEEKCATPVEKLKQVA
DAMTVEMQAGLASEGGSKLKMLISYVDNLPTGDEKGLFYALDLGGTNFRVMRVQLGGKEK
RIVKHEVKEVSIPQNVMTGSSSEVLFDFIATALAEFVATEGDDFHLPPGRQRELGFTFSF

PVKQLSIASGTLIKWTKGFSIEDLVGQDVVGELAKAMERAGLDVRVAALVNDTVGTLAGG
RYNNPDVIAAVILGTGTNAAYVERAHAIPKWHGLLPKSGEMVINMEWGNFRSSHLPVTEY
DQNLDIESLNPGEQIYEKIISGMYLGEILRRVLCRMAEEASLFGDYVPSKLKVPFVLRTP
DMAAMHHDESADLKVVGNKLKDILEVPNSTLKMRKIVVELCDIITSRGARLSAAGIVGIL
KKLGRDTFKDGEKQRSVIAVDGALFEHYTKFRNCLKETMKELLGDAADSTVIELSNDGSG
VGAALLAASHSQYTDLEES
>Sly|NP_001234717.1
MSVTVSSPAVRSFHVSRSPHKTISRPRVIISAVRSTDSLGVAPILTKLQKDCATPLPVLR
HVADAMADDMRAGLAVDGGSDLKMILSYVDTLPTGNEKGLFYALDLGGTNFRVLRVQLGG
KEERVVATEFEQVSIPQELMFATSEELFDFIASALGKFAQKEGGNFELQQGRTREIGFTF
SFPVKQTSIRTGILIKWTKGFAVSGTAGKDVVACLNEAMERRGMDMQVSALVNDTVGTLA
GARYWDDDAMVAVILGTGTNACYVERVDAIPKLAKRMSKSPITIVNTEWGAFSNGLPLTE
FDREMDAESINPGEQIFEKTISGMYLGEIVRRVLVKMAKVGGLFGSSYVPEKLVTPFVLR
TPDICAMQQDTSIDLEAVESVLYDVAGVKSDLSARKTVVDICDTIAKRGGRLAGAGIVGI
LQKMEEDSKGLIFGKRTVVAMDGGLYEHYPQYRGYLQEAVTELLGSEISKNVVIEHSKDG
SGIGAALLAAANSKYEHDD
>Sly|XP_004231963.1
MKKDVVVLAAATTISTIVAAVLLVRQWKRRSEQRWRHAQRILRKFARECATPIPKLWQIA
DDLVAQMQSGLNSTQSTLQMLPSCLPSLPNGDEKGLFYGINLRGTNFIIVQARLGGRNAP
MSRIGGRCEPISDLYRQEISIPPNIIEASSQELFDWITVELGKFISLHSEGLQGGEKNLG
FTVSPTIAEVAASRETAITWKDSLLGDAAGNKLLNEINVAMEKHSVDKRVFSLVDDTIGV
LAGGRYYSKESVAAVTLGMGTNAAYIESAQSVVKWPDQTPKPEEIAINTHWGNFRSSHLP
ITEFDTSLDAESSYPGSQIFEKLISGTYLGETVRRVLLKMAQESALFGDIVPPKLAIPYL
LRSPDMAAMHQDTSEDYEIIDEKLGEIFEITNSTTMARELVAEICDVVAERGARLVGAGI
VGIVKKLDRLSNRISIITVEGGVYEHYRVFRNYLHSSVWEMLGNEFSDNVIIEHSHGGSG
ASSIYIAASQP
>Sly|XP_004251354.1
MGRLGVGLAVGCAMAACIVAAAMVGKRVNKRSKWLKMVKVLEELEESCDTTVFRLKQVVD
AMAVEMHAGLASEGGSKLKMLLTYVDKLPNGREEGTYYALDLGGTNFRVLRVHLGDQRSA
ILGQDIERQPIPQHLMTSTSEDLFDFIASSLKDFIEKEGNDLEQTSPRRRGLGFTFSFPV
KQSSVSSGILMKWTKGFAIEDTIGRDVSECLQLALSRKGLDVRVAALINDTVGTLALGHY
NDEDTVAAVIIGTGTNACYLERADAIIKCQGLLTTSGSMVVNMEWGNFWSSHLPRTSYDI
DLDVASPNPNDQGFEKMISGMYLGDIVRRVLLRMSKQSDDFGPSSSKLAVPFVLRTPLMA
AMHEDDSPDLSEVAKILEEVLELPDVPVKVRKLVVKVCDVITRRAARLAAAGIVGILKKI
GRDGSGGIASGKFRSNRPSRLRRTVVAIEGGLYTSYTTFREYLNEAMAEILGEEVSPYVI
LRVMEDGSGTGAALVAAANSLPEAADTVQLQ
>Sly|XP_010313255.1
MGRLGVGLAVGCAMAACIVAAAMVGKRVNKRSKWLKMVKVLEELEESCDTTVFRLKQVVD
AMAVEMHAGLASEGGSKLKMLLTYVDKLPNGREEGTYYALDLGGTNFRVLRVHLGDQRSA
ILGQDIERQPIPQHLMTSTSEDLFDFIASSLKDFIEKEGNDLEQTSPRRRGLGFTFSFPV
KQSSVSSGILMKWTKGFAIEDTIGRDVSECLQLALSRKGLDVRVAALINDTVGTLALGHY
NDEDTVAAVIIGTGTNACYLERADAIIKCQGLLTTSGSMVVNMEWGNFWSSHLPRTSYDI
DLDVASPNPNDQGFEKMISGMYLGDIVRRVLLRMSKQSDDFGPSSSKLAVPFVLRTPLMA
AMHEDDSPDLSEVAKILEEVLELPDVPVKVRKLVVKVCDVITRRAARLAAAGIVGILKKI
GRDGSGGIASGKFRSNRPSRLRRTVVAIEGGLYTSYTTFREYLNEAMAEILGEEVSPYVI
LRVMEDGSGTGAALVAAANSLPEAADTVQLQ
>Sly|XP_010322313.1
MKKATVGAVVVGTAAAVAVAALVMRHRMGKSSKWARARAILKEFEEKCATPDAKLKQVAD
AMTVEMHAGLASEGGSKLKMLISYVDNLPTGDEAGVFYALDLGGTNFRVLRVQLGGKDGG
IMHQEFAEASIPPNLMVGTSEALFDYIAAELAKFVDEEGEKFHPPPGKQRELGFTFSFPI
MQTSINSGTLIRWTKGFSIDDTVGKDVVAELTKAMQKREIDMRVSALVNDTVGTLAGGRF
TDKDVSIAVILGTGTNAAYVERAQAIPKWHGPLPNSGEMVINMEWGNFRSSHLPLTQYDN
AMDTDSLNPGEQIFEKICSGMYLGEILRRVLLRMAKEAGIFGEEVPPKLKNSFILRTPEM
SAMHHDTSSDLRVVGDKLKDILEISNTSLKTRRLVVELCNIVATRGARLAAAGILGIIKK

MGKDTPRESGPEKIVVAMDGGLYEHYTEYSKCLENTLVELLGKEMATSIVFKHANDGSGI
GAALLAASNSVYVEDK
>Sly|XP_019066821.1
MRVQLGGKEKRIVKHEVKEVSIPQNVMTGSSSEVLFDFIATALAEFVATEGDDFHLPPGR
QRELGFTFSFPVKQLSIASGTLIKWTKGFSIEDVVGQDVVGELAKAMERAGLDVRVAALV
NDTVGTLAGGRYNNPDVIAAVILGTGTNAAYVERAHAIPKWHGLLPKSGEMVINMEWGNF
RSSHLPVTEYDQNLDIESLNPGEQIYEKIISGMYLGEILRRVLCRMAEEASLFGDYVPSK
LKVPFVLRTPDMAAMHHDESADLKVVGNKLKDILEVPNSTLKMRKIVVELCDIITSRGAR
LSAAGIVGILKKLGRDTFKDGEKQRSVIAVDGALFEHYTKFRNCLKETMKELLGDAADST
VIELSNDGSGVGAALLAASHSQYTDLEES
>Sly|XP_025885482.1
MKKDVVVLAAATTISTIVAAVLLVRQWKRRSEQRWRHAQRILRKFARECATPIPKLWQIA
DDLVAQMQSGLNSTQSTLQMLPSCLPSLPNGDEKGLFYGINLRGTNFIIVQARLGGRNAP
MSRIGGRCEPISDLYRQEISIPPNIIEASSQELFDWITVELGKFISLHSEGLQGGEKNLG
FTVSPTIAEVAASRETAITWKDSLLGDAAGNKLLNEINVAMEKHSVDKRVFSLVDDTIGV
LAGGRYYSKESVAAVTLGMGTNAAYIESAQSVVKWPDQTPKPEEIAINTHWGNFRSSHLP
ITEFDTSLDAESSYPGSQIFEKLISGTYLGETVRRVLLKMAQESALFGDIVPPKLAIPYL
LRSPDMAAMHQDTSEDYEIIDEKLGEIFEEGVKHVICLIKVLLCRRLHTSRFVYRFLLAT
LAN
>Sly|XP_025885483.1
MKKDVVVLAAATTISTIVAAVLLVRQWKRRSEQRWRHAQRILRKFARECATPIPKLWQIA
DDLVAQMQSGLNSTQSTLQMLPSCLPSLPNGDEKGLFYGINLRGTNFIIVQARLGGRNAP
MSRIGGRCEPISDLYRQEISIPPNIIEASSQELFDWITVELGKFISLHSEGLQGGEKNLG
FTVSPTIAEVAASRETAITWKDSLLGDAAGNKLLNEINVAMEKHSVDKRVFSLVDDTIGV
LAGGRYYSKESVAAVTLGMGTNAAYIESAQSVVKWPDQTPKPEEIAINTHWGNFRSSHLP
ITEFDTSLDAESSYPGSQIFEKLISGTYLGETVRRVLLKMAQESALFGDIVPPKLAIPYL
LRSPDMAAMHQDTSEDYEIIDEKLGEIFEEGVKHVICLIKVLLYN
>Sly|XP_025885484.1
MKKDVVVLAAATTISTIVAAVLLVRQWKRRSEQRWRHAQRILRKFARECATPIPKLWQIA
DDLVAQMQSGLNSTQSTLQMLPSCLPSLPNGDEKGLFYGINLRGTNFIIVQARLGGRNAP
MSRIGGRCEPISDLYRQEISIPPNIIEASSQELFDWITVELGKFISLHSEGLQGGEKNLG
FTVSPTIAEVAASRETAITWKDSLLGDAAGNKLLNEINVAMEKHSVDKRVFSLVDDTIGV
LAGGRYYSKESVAAVTLGMGTNAAYIESAQSVVKWPDQTPKPEEIAINTHWGNFRSSHLP
ITEFDTSLDAESSYPGSQIFEKLISGTYLGETVRRVLLKMAQESALFGDIVPPKLAIPYL
LRSPDMAAMHQDTSEDYEIIDEKLGEIFERRRGLSM
>Sly|XP_025885485.1
MKKDVVVLAAATTISTIVAAVLLVRQWKRRSEQRWRHAQRILRKFARECATPIPKLWQIA
DDLVAQMQSGLNSTQSTLQMLPSCLPSLPNGDEKGLFYGINLRGTNFIIVQARLGGRNAP
MSRIGGRCEPISDLYRQEISIPPNIIEASSQELFDWITVELGKFISLHSEGLQGGEKNLG
FTVSPTIAEVAASRETAITWKDSLLGDAAGNKLLNEINVAMEKHSVDKRVFSLVDDTIGV
LAGGRYYSKESVAAVTLGMGTNAAYIESAQSVVKWPDQTPKPEEIAINTHWGNFRSSHLP
ITEFDTSLDAESSYPGSQIFEKLISGTYLGETVRRVLLKMAQESALFGDIVPPKLAIPYL
LRSPDMAAMHQDTSEDYEIIDEKLGEIFERRRGLSM
>Sbi|XP_002440104.1
MGKAVVVGTAVVACAAVGVALALAHRRRKRDAELLGSAEAERKRRAAAVIEDVESSLATP
TALLRSIADAMVTEMERGLRGDIHSQLKMLISYVDNLPTGDEHGLFYALDLGGTNFRVLR
VQLGGREKRVVKQQYEEVSIPPHLMVGTSMELFDFIAAALAKFVDTEGEDFHLPEGRLRE
LGFTFSFPVNQTSISSGTLIKWTKGFSINGTVGEDVVSELSRAMERQGLDMKVTALVNDT
VGTLAGGRYMDNDVVAAVILGTGTNAAYVEHANAIPKWTGLLPKSGNMVINTEWGSFKSD
KLPLSEYDKAMDFESLNPGEQIYEKMISGMYLGEIVRRILLKLAHDASLFGDVVPSKLEQ
PFILRTPDMSAMHHDSSHDLKTLGAKLKDIVGVADTSLEVRYITRHICDLVAERGARLAA
AGIYSILKKIGRDKVPSSGGKMPRTVVALDGGLYEHYKKFSSCVEATLTDLLGEEASSSV
VAKLANDGSGIGAALLAASHSQYGESH
>Sbi|XP_002440735.1

MVAAAAERVVAELREACAAPAARLNEVAAAMEAEMRAGLREEGGSKIKMIISYVDNLPTG
NEEGVFYALDLGGTNFRVLRVHLAGKDKRVAKRESKEVSIPPHLMSGNASELFGFIASAL
AKYIASEEGHSNVFDDDKQRELGFTFSFPVRQTSIASGTLIKWTKAFSIDDAVGEDVVAE
LQTAMEKQGVDMRVAALINDTVGTLAAGRYNDEDVVIGVILGTGSNAAYVEEASAIPKLE
GELPKSGNMVINTEWGNFDSSCLPITEYDEALDEESLNPGEQIFEKLISGMYLGEIVRRV
LLKIASQSSIFGNVSHTLLKTRFILRTPDISAMHHDDTPDLRIVAQKLADNLKIMDTSLD
TRKMVVEICDIVTSRSARLAAAGIVGILRKIGRAVPGDERRSVVAIDGGLFEHYAEFRKC
LESTLVELLGEEASKSLVVKLTKDGSGLGAALIAAAQSQ
>Sbi|XP_002458467.1
MGKGAVVGTAVVVGAAAAAAVGVAVVSLSRRRRRRREAEDERKRKAAAVIEEVEQRFSTP
TALLRGIADAMVEEMERGLRAEPHAPLKMLISYVDNLPTGDEQGLFYALDLGGTNFRVIR
VQLGGRDKRVVKQQYEEVSIPPHLMVGTSTELFDFIAAELEKFVRTEGEDFHLPNGKQRE
LGFTFSFPVHQTSISSGTLIKWTKGFSINGTVGEDVVAELSRAMERQGLDMKVAALVNDT
VGTLAGGRYADNDVVTAVILGTGTNAAYVEHANAIPKWTGLLPRSGNMVINMEWGNFRSD
KLPMSEYDKSLDFESLNPGEQIYEKMISGMYLGEIVRRILLKLAHDASLFGDVVPPKLEQ
LFILRTPDMSAMHHDTSHDLKHLGAKLKDILGVPDTSLEARYITLHVCDLVAERGARLAA
AGIYGILKKLGKDKLLSDFSQQRTVVAIDGGLYEHYKKFSACLEATLTDLLGEEVASSVV
VKLANDGSGIGAALLAASHSQYAEVA
>Sbi|XP_002459072.1
MGRVGLGVAAGCAAATCAIAAALVARRASARARWRRAVALLREFEEGCATPTPRLRQVVD
AMVVEMHAGLASDGGSKLKMLLTFVDALPAGNEQGTYYSIDLGGTNFRVLRVEVGAVSVV
TSREVKLPIPEELTKGTIEELFNFVAMTLKEFVETEDVKDEQRALGFTFSFPVRQTSVSS
GSLIRWTKGFLIEDAVGKDVAQCLNEALARSGLNVRVTALVNDTVGTLALGHYYDEDTVA
AVIIGAGTNACYIERTDAIIKCQGLLTNSGGMVVNMEWGNFWSSHLPRTPYDISLDDETQ
NRNDQGFEKMISGIYLGEIARLVLHRMALESDVFGDAADHLSTPFTLSTPLLAAIRKDDS
PDLSEVRRILQEHLKIMDTPLKTRRLVVKVCDIVTRRAARLAAAGIVGILKKLGRDGSGV
ASSGRTRGQLRRTVVAIEGGLYEGYPVFREYLDEALVEILGEEVAQTVALRVTEDGSGAG
AALLAAVHSSNRQQGSI
>Sbi|XP_021312505.1
MRKPAALVASTVVFAAAAVAAVMARQRLREARRWARAAAMLRDLQERCAAPAERLRQVAD
AMAAEMRAGLASNDSEGESGSTVLLKMLVTYVDSLPSGGEKGLFYALDLGGTNFRVLRIQ
FGGKEQRIVKQESKGVSIPQHLMSRGSNELFDFIAAALAKFVASEGEDFHLPEGMQRQLG
FTFSFPVNQNSVASGTLIKWTKGFAIDEMVGKDVVAELNKAIERQGIDMKITALVNDTVG
TLAAGRYVDNDTVAAVILGTGTNAAYIEHMNSIPKWCGPPPESDDMVINMEWGNFRSSHL
PLTEFDVALDAESLNPGEQIYEKLISGMYMGEIVRRVLLKMAQDASLFADNVPPKLEIPY
ILRTYHVLMMHQDTSPDLRTVGINLKEIFGIENTCCKTRKLVVDVCEAVATRGARLAAAG
IHGILKKLGRDIANPDKQKTVIAVDGGVYKYYTFFAQCMESTLRDLLGEEVASSVVIKLA
EDGSGTGAALLAASYSQRFGAMDD
>Vvi|NP_001267834.1
MSVSAVGSFWLSPSPGKLPRPRMVVRSSAVSVAPILTHLQKQCATPLPVLRHVADAMAAD
MRAGLAVDGGSDLKMILSYVDTFPTGNEKGLFYALDLGGTNFRVLRVQLGGKDERVIATE
FEQVTIPQELMFGTSEELFDFIACGLANFAKKEGGKFHLPSGRKREIGFTFSFPVKQTSI
DSGILMKWTKGFAVSGTAGRDVVACLNEAMERQGLDMQVSALVNDTVGTLAGARYWDDDV
MVAVILGTGTNACYVERTDVIPKLQGQMSSSGRTIISTEWGAFSNGLPLTEFDRDMDAAS
INPGEQIFEKTISGMYLGEIVRRVLLKMAEAGALFGEYVSEKLSTPFILRTPDICAMQQD
NSDHLDAVGAILYDVAGVKSDLSVRKMVVEVCDTIVKRGGRLAGAGIVGILQKMEEDSKD
LIFGKRTVVAMDGGLYENYPQYRRYLKEAVTELLGLEMSKNVVIEHSKDGSGIGAALLAA
SNSKY
>Vvi|XP_002283608.1
MGKVAVGAAVVCAATVCAAAVLIARHRMRSSGRWARAMAILREFEEKCGTPIAKLRQVAD
AMTVEMHAGLASEGGSKLKMIISYVDNLPTGDEKGLFYALDLGGTNFRVLRVQLGGKDKR
VVKQEFEEVSIPPHLMVGSSDALFDYIAAALAKFVATEGEGLHVSPGRQRELGFTFSFPV
RQSSISSGSLIKWTKGFSIEDAVGQDVVGELTKAMERIGLDMRVSALVNDTIGTLAGGRY
YDQDVVAAVILGTGTNAAYVERAQSIPKWHGLLPKSGDMVINMEWGNFRSSHLPLTEYDQ

VLDAESLNPGEQIFEKIISGMYLGDIVRRVLCRMAEEADLFGDTVPPKLKIPFILRTPDM
SAMHHDTSPDLRVVGSKLKDILDIPNTSLKTRKVVIELCDIVATRGARLSAAGILGILKK
LGRDTMKEGDKQNSVIALDGGLYEHYTEFRTCLESTLKELLGAEVSDNIVIKHSNDGSGI
GAALLAASHSQYLEEDS
>Vvi|XP_003635485.1
MRKAVVVAAVTTAAMAAAVAVLWSRWKQESERQWRKTRRILRKFARDCATPVSKLWLVAD
ALVSDMNEALTSQETTTLNMPISYVASLPSGDEEGLYYGLNLRGSNFLILRARLRGKNES
ISELHREEVSIPSNVMGGTSQELFDYIALELAKFISEHEVTTDDTPDRQKALGFIVSYPV
DQAAASSGAAIKWRSFSVTDTAGKALASNFNRALEKHGVNLRVLQLVEDAIGHLAGGRYY
NRDTVAAITLGMATTAAYVEPTQAVPEYQSSSNKSSEMVISMDWGNFNSCHLPITEFDTF
LDAESSNPGSRIFEKLTSGMYLGEIVRRVLLKMAQETALFGEDVPPKLRTPYLLRSPDMA
AMHQDTSEDHEVVHEKLMEIFRITNSTPLAREVVAEVCDIVAERGARLAGAGIVGITKKL
GRIENKRSAVTVEGGLYEHYRVFRNYMHSSVWEMLGNELSDNVVIEHSHGGSGAGAIFLA
ASQTNEPNS
>Vvi|XP_019076347.1
MGKVGFGLAVSCAAATCAIAAILVGKRVRSRRKWRRVVGVLRDLEEACETSVGRLRQVVD
AMAVEMHAGLASEGGSKLKMLLTFVDKLPNGSERGKYYALDLGGTNFRVLQIQLGGKTSS
ILSCDVERQPIPQDLMTSTSEDLFDFIASSLKQFVEREAQNSEFSPVKRRELGFTFSFPV
KQTSVSSGILIKWTKRFSVKDMVGKDVSECLQHAMTRNGLDMRVAVLVNDTVGTLALGHY
NDPDTVAAVIIGTGTNACYLERTDAIIKCQGLLTTSGGMVVNMEWGNFWSSHLPRTSYDI
DLDGDSPNPNDQGFEKMISGMYLGDIVRRVILRMSQESDIFGPVSSRLSMPFILSTPSMA
DMHADESPELMEVARILKEDLQIPDVPLKVRKLVVKVCDVVTRRAARLAAAGIVGILKKI
GRDGSGGVASGRSRGSDGKMRRTVVAMDGSLYTRYTMFKEYLHEALCEILGEEVAQYVIL
KVTEDGSGIGAALLAASHSSHSSSSSSSSSSSSSSVVDSQRLP
>Zma|NP_001105529.2
MVKAVVVGAAVVACAAVGVAVVLERRRRKRDADLLGSAEAERRRRAAAVIEEVESSLATP
TALLRSIADAMVAEMERGLRGDIHAQLKMLISYVDSLPTGDEHGLFYALDLGGTNFRVLR
VQLGGKEKRVVKQQYEEVSIPPHLMVGTSMELFDFIAAALAKFVGTEGEDFQLPEGRQRE
LGFTFSFPVNQTSISSGTLIKWTKGFSINGTVGEDVVSELSRAMERQGLDMKATALVNDT
VGTLAGGRYMDTDVVAAVILGTGTNAAYVEHANAIPKWTGLLPKSGKMVINTEWGSFKSN
KLPLSEYDKAMDFESLNPGEQIYEKMISGMYLGEIVRRILLKLAHEASLFGDVVPPKLEL
PFILRTPDMSAMHHDSSHDLKTLGAKLKDIVGVADTSLEVRYITRHICDLVAERAARLAA
AGVYSILKKIGRDKVPSGGGKMPRTVVALDGGLYEHYKKFSSCVEATLTDLLGEEASSSV
VAKLANDGSGIGAALLAASHSQYQYGASD
>Zma|NP_001123599.1
MVKAVVVGAAVVACAAVGVAAVLVHRRRRRDAALLGSAEAERRRRAAAVIEEVERSLATP
TALLRGIADAMVAEMERGLRGDIHAQLKMLISYVDNLPTGDEHGLFYALDLGGTNFRVLR
VQLGGREKRVVKQQYEEVSIPPHLMVGTSMELFDFIAAALAKFVGTEGEDFQLPEGRQRE
LGFTFSFPVNQTSISSGTLIKWTKGFSINGTVGEDVVSELSRAMERQGLDMKATALVNDT
VGTLAGGRYMDTDVVAAVILGTGTNAAYVEHANAIPKWTGLLPKSGKMVINTEWGSFKSN
KLPLSEYDKAMDFESLNPGEQIYEKMISGMYLGEIVRRILLKLAHDASLFGDVVPTKLEQ
PFILRTPDMSAMHHDSSHDLKTLGSKLKDIVGVADTSLEVRYITRHICDLVAERGARLAA
AGIYSILKKIGRDKVPSSGSKMPRTVIALDGGLYEHYKKFSSCVEATLTDLLGEEASSSV
VAKLANDGSGIGAALLAASHSQYGASD
>Zma|NP_001146056.1
MRKPAALVVSTAVFAAAAVVAVMARQRLREARRWARAGAVLRDLQERCAAPVERLRQVAD
AMVAEMRAGLASNDSEGDSGSSVLLKMLVTYVDSLPSGDEKGLFYALDLGGTNFRVLRIQ
FGGKEQRIVKQESKTVSIPQHLMSRGSNELFDFIAAALAKFIASEGEGFYLPEGMQRQLG
FTFSFPVKQNSVASGTLIKWTKGFAIDEMVGKDVVAELNKAIKRQGVDMEVTVLVNDTVG
TLAAGRYVDNDTVAAVILGTGTNAAYIEHMDSIPKWCGPPPESGNMVINMEWGNFRSTHL
PLTEFDVALDAESLNPDEQIYEKLISGMYMGEIVRRVLLKMAQDASLFSDNVPLELEVPY
ILRTYHVLMMHQDTSPDLRTVGNKLKEVFRIEDTCFKTRKLVVDVCEVVASRAARLAAAG
IHGILKKLGRDVASPDKQKTVIAVDGGVYKHYTFFAQCMESTLRDLLGEGVASSLVIKLA
EDGSGTGAALLSASYSQRLQAVDD

>Zma|NP_001356812.1
MVAAADLEAAERVVAELREACAARLDEVAAAMEAEMRAGLREEGGSKIKMIVSFVDNLPT
GNEEGVFYALDLGGTNFRVLRVQLAGKDRRVAKRDSKEVSIPPHLMSGNASELFGFIASA
LAKYVAAAGEGDGRQRELGFTFSFPVRQTSIASGTLIKWTKAFSVDDAVGEDVVAELQTA
MEKQGVDMRVAALINDTVGTLAAGRYNDEDVVVGVILGTGSNAAYVEEASAIPKLKLEGG
ELPKSGNMVINTEWGNFYSPCLPITEYDQALDEESLNPGEQIFEKLVSGMYLGEIVRRVL
LKIASQSSILGDVSHTNLKTRFSLRTPDISAMHHDGTPDLRVVAEKLADNLRVRDTSLDT
RKMVVEICDIVTGRSARLAAAGIVGILRKIGRAAPGDERRSVVAIDGGLFEHYAEFRKRL
EATLVELLGEEASRLVEVKLTKDGSGLGAALIAAAHSQY
>Zma|XP_008656880.1
MGRVGLGVAAGCAAATCAIAAALVARRASARARWRRAVALLREFEDGCATPPPRLRQVVD
AMVVEMHAGLASDGGSKLKMLLTFVDALPAGNEEGTYYSIDLGGTNFRVLRVEVGAGSVV
TRRKVELPIPEELTKGTIEELFNFVAMTLKEFVETEDGNDEQRALGFTFSFPVRQTSVSS
GSLIRWNKGFLIEDAVGKDVAQCLNEALARNGLNVRVTALVNDTVGTLALGHYHDEDTVA
AVIIGAGTNACYIERTDAIIKCQGLLTNSGGMVVNMEWGSFWSSHLPRTPYDISLDDETQ
NRNDQGFEKMVSGIYLGEIARLVLHRMALESDFFGDAADNLCTPFTLSTPLLAAIRKDDS
PDLSEVRKILQEHLKIPDTPLKTRRLVVKVCDIVTRRAARLAAAGIVGILKKLGRDGSGV
ASSGRTGGQMRRTVVAIEGGLYEGYPVFREYLDEALVEILGEEVARTVALRVTVDGSGAG
AALLAAVHSSNRQQGSI
>Zma|XP_008674565.1
MGRVGLGVAAGCAAATCAIAAALVARRAAARSRWRRAVALLREFEDGCATPPPRLRQVVD
AMVVEMHAGLASDGGSKLKMLLTFVDALPAGNEEGTYYSIDLGGTNFRVLKVEVGDGSVV
TRRKVELPIPEELIKGTIEELFNFVAVTLKEFVEAEDGKDEQRALGFTFSFPVRQTSVSS
GSLIRWTKGFLIEDVVGKDVAQCLNEALARSGLNVRVTALVNDTVGTLALGHYHDEDTVA
AVIIGAGTNACYIERTDAIIKCQGLLTNSGGMVVNMEWGNFWSSHLPRTPYDISLDDETQ
NRNDQGFEKMISGIYLGEIARLVLHRMALESDVFGDAADNLSTPFTLSTPLLAAIRKDDS
PDLSEVRRILQDHLKIPDTPLKTRRLVVKVCDIVTRRAARLAAAGIVGILKKLGRDGSGI
ASSGRTRGQLRRTVVAIEGGLYEGYPEFREYLDEALVEILGEEVARTVTLEVTVDGSGAG
AALLAAVHSSNRQQGSI
>Zma|XP_008675068.1
MAKGGAVVGTAVVVCAAAAAAVGVAVVLSRRRSRRREAEDERKRKAAAVIEEVEQRLSTP
TALLRGIADAMVEEMERGLRAEPHAPLKMLISYVDNLPTGDEHGLFYALDLGGTNFRVIR
VQLGGRDRRVVKQQYEEVSIPPHLMVGTSTELFDFIAAELEKFVRTEGEDFHLPDSKQRE
LGFTFSFPVHQTSISSGTLIKWTKGFCINGTVGEDVVAELSRAMERQGLDMKVAALVNDT
VGTLAGGRYADNDVVAAVILGTGTNAAYVEHANVIPKWTGLLPRSGNMVINMEWGNFRSD
KLPRSEYDKSLDFESLNPGEQIYEKMISGMYLGEIVRRILLKLAHDASLFGDVVPPKLEQ
LFILRTPDMSAMHHDTSHDLKHLGAKLKDILGVPDTSLEARYITLHVCDLVAERGARLAA
AGIYGILKKLGKDKLLGDFSQQRTVVAIDGGLYEHYKKFSACLEATLTDLLGEEVASSVV
VKLANDGSGIGAALLAASHSQYAEAA
>Zma|XP_035817780.1
MGRVGLGVAAGCAAATCAIAAALVARRASARARWRRAVALLREFEDGCATPPPRLRQVVD
AMVVEMHAGLASDGGSKLKMLLTFVDALPAGNEEGTYYSIDLGGTNFRVLRVEVGAGSVV
TRRKVELPIPEELTKGTIEELFNFVAMTLKEFVETEDGNDEQRALGFTFSFPVRQTSVSS
GSLIRWNKGFLIEDAVGKDVAQCLNEALARNGLNVRVTALVNDTVGTLALGHYHDEDTVA
AVIIGAGTNACYIERTDAIIKCQGLLTNSGGMVVNMEWGSFWSSHLPRTPYDISLDDETQ
NRNDQGFEKMVSGIYLGEIARLVLHRMALESDFFGDAADNLCTPFTLSTPLLAAIRKDDS
PDLSEVRKILQEHLKIPDTPLKTRRLVVKVCDIVTRRAARLAAAGIVGILKKLGRDGSGV
ASSGRTGGQMRRTVVAIEGGLYEGYPVFREYLDEALVEILGEEVARTVALRVTVDGSGAG
AALLAAVHSSNRQQGSI
>Zma|XP_035817781.1
MGRVGLGVAAGCAAATCAIAAALVARRASARARWRRAVALLREFEDGCATPPPRLRQVVD
AMVVEMHAGLASDGGSKLKMLLTFVDALPAGNEEGTYYSIDLGGTNFRVLRVEVGAGSVV
TRRKVELPIPEELTKGTIEELFNFVAMTLKEFVETEDGNDEQRALGFTFSFPVRQTSVSS
GSLIRWNKGFLIEDAVGKDVAQCLNEALARNGLNVRVTALVNDTVGTLALGHYHDEDTVA

AVIIGAGTNACYIERTDAIIKCQGLLTNSGGMVVNMEWGSFWSSHLPRTPYDISLDDETQ
NRNDQGFEKMVSGIYLGEIARLVLHRMALESDFFGDAADNLCTPFTLSTPLLAAIRKDDS
PDLSEVRKILQEHLKIPDTPLKTRRLVVKVCDIVTRRAARLAAAGIVGILKKLGRDGSGV
ASSGRTGGQMRRTVVAIEGGLYEGYPVFREYLDEALVEILGEEVARTVALRVTVDGSGAG
AALLAAVHSSNRQQGSI
>Zma|XP_035817782.1
MGRVGLGVAAGCAAATCAIAAALVARRASARARWRRAVALLREFEDGCATPPPRLRQVVD
AMVVEMHAGLASDGGSKLKMLLTFVDALPAGNEEGTYYSIDLGGTNFRVLRVEVGAGSVV
TRRKVELPIPEELTKGTIEELFNFVAMTLKEFVETEDGNDEQRALGFTFSFPVRQTSVSS
GSLIRWNKGFLIEDAVGKDVAQCLNEALARNGLNVRVTALVNDTVGTLALGHYHDEDTVA
AVIIGAGTNACYIERTDAIIKCQGLLTNSGGMVVNMEWGSFWSSHLPRTPYDISLDDETQ
NRNDQGFEKMVSGIYLGEIARLVLHRMALESDFFGDAADNLCTPFTLSTPLLAAIRKDDS
PDLSEVRKILQEHLKIPDTPLKTRRLVVKVCDIVTRRAARLAAAGIVGILKKLGRDGSGV
ASSGRTGGQMRRTVVAIEGGLYEGYPVFREYLDEALVEILGEEVARTVALRVTVDGSGAG
AALLAAVHSSNRQQGSI
>HXK3-Mazzard-F12/1
MSVAATSPAVGSFYLRRRSPSTAMSMPRFRMAVRSAAVSVTPLLTKLHNDSATPLPLLRH
VADAMAADMRAGLAVEGGSDLKMILSYVDSLPSGNEEGLFYALDLGGTNFRVLRVQLGGK
QERVIATEFDQVSIPKELMFGTSEQLFDFIAAGLAKFAQKEGNKFHLPTGTKREIGFTFS
FPVKQTSIDSGILIKWTKGFSVSGTAGKDVVACLNEAMERQGLDMRVSALVNDTVGTLAG
ARYWDDDVMVAVILGTGTNACYVERADAIPKLQGQVSSSGKTIINTEWGAFSDGLPLTEF
DREMDAASINPGEQIFEKTISGMYLGEIVRRVLVKMAEADALFGESVPEKLSTPFVLRTP
DICAMQQDISDDLQDVGSILYNVAGVESNLSARKIVVDVCDTIVKRGGRLAGAGIVGILQ
KMEEDSKDLIFGKRTVVAMDGGLYENYPQYRRYLQEAVTELLGTEISKNVVIEHSKDGSG
IGaallAAANSQYA
>HXK3-Mariana2624
MSVAATSPSVGSFYLRRRSPSTPMSMPRFRMAVRSAAVSVTPLLTKLHNDSATPLPLLRH
VADAMAADMRAGLAVEGGSDLKMILSYVDSLPSGNEEGLFYALDLGGTNFRVLRVQLGGK
QERVIATEFDQVSIPKELMFGTSEQLFDFIASGLAKFAQKEGNKFHLPTGTKREIGFTFS
FPVKQTSIDSGILIKWTKGFSVSGTAGKDVVACLNEAMERRGLDMRVSALVNDAVGTLAG
ARYWDDDVMVAVILGTGTNACYVERAEAIPKLQGQVSSSGRTIINTEWGAFSDGLPLTEF
DREMDAASINPGEQIFEKTISGMYLGEIVRRVLVKMAEADALFGESVPEKLSTPFALRTP
DICAMQQDISDDLQDVGSILYNVAGVESNLSARKIVVDVCDTIVKRGGRLAGAGIVGILQ
KMEEDSKGLIFGKRTVVAMDGGLYANYPQYRRYLQEAVTELLGTEISKNVVIELTKDGSG
IGaallAAANSQYA
